# Supplementary material for: Ambiphilic boron in 1,4,2,5-diazadiborinine
Source: Nat Commun. 2016 Jun 9;7:11871. doi: 10.1038/ncomms11871 (PMC4906229; doi:10.1038/ncomms11871)
Supplement: Supplementary information — Supplementary Figures 1-59, Supplementary Tables 1-8, Supplementary Methods and Supplementary References [file ncomms11871-s1.pdf]

Supplementary Figures

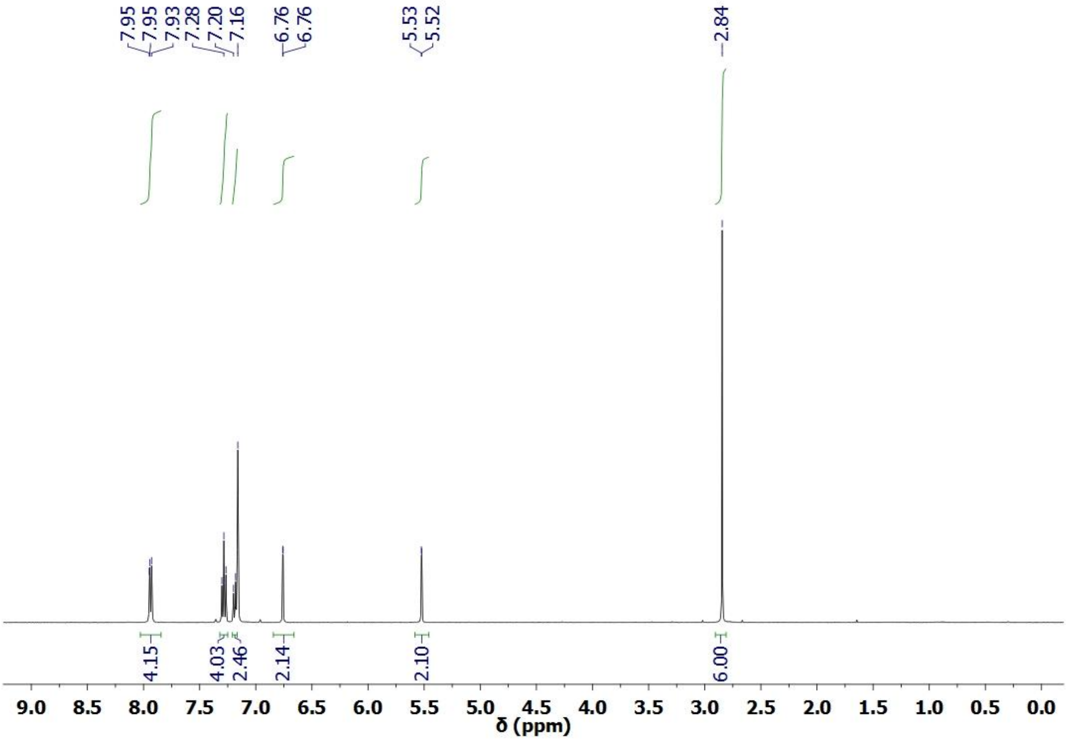

Supplementary Figure 1 | ^{1}H NMR spectrum (C_6D_6) of **1**.

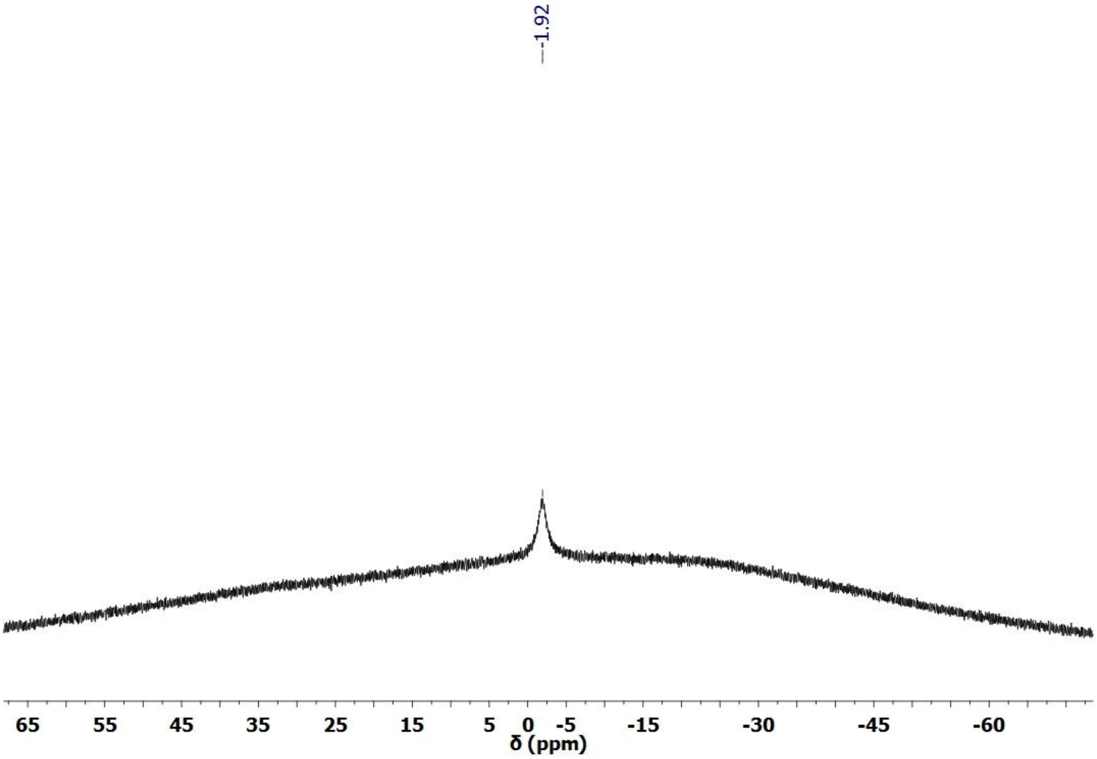

Supplementary Figure 2 | $^{11}B\{^{1}H\}$ NMR spectrum (C_6D_6) of **1**.

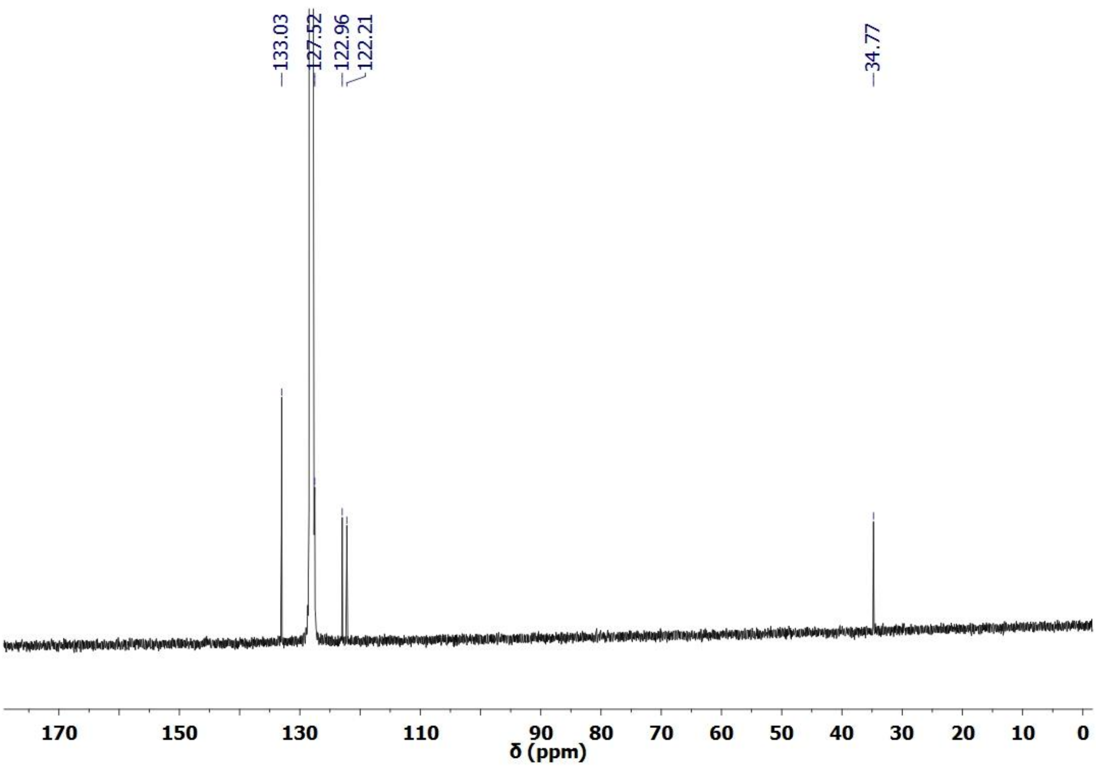

Supplementary Figure 3 | ^{13}C NMR spectrum (C_6D_6) of **1**.

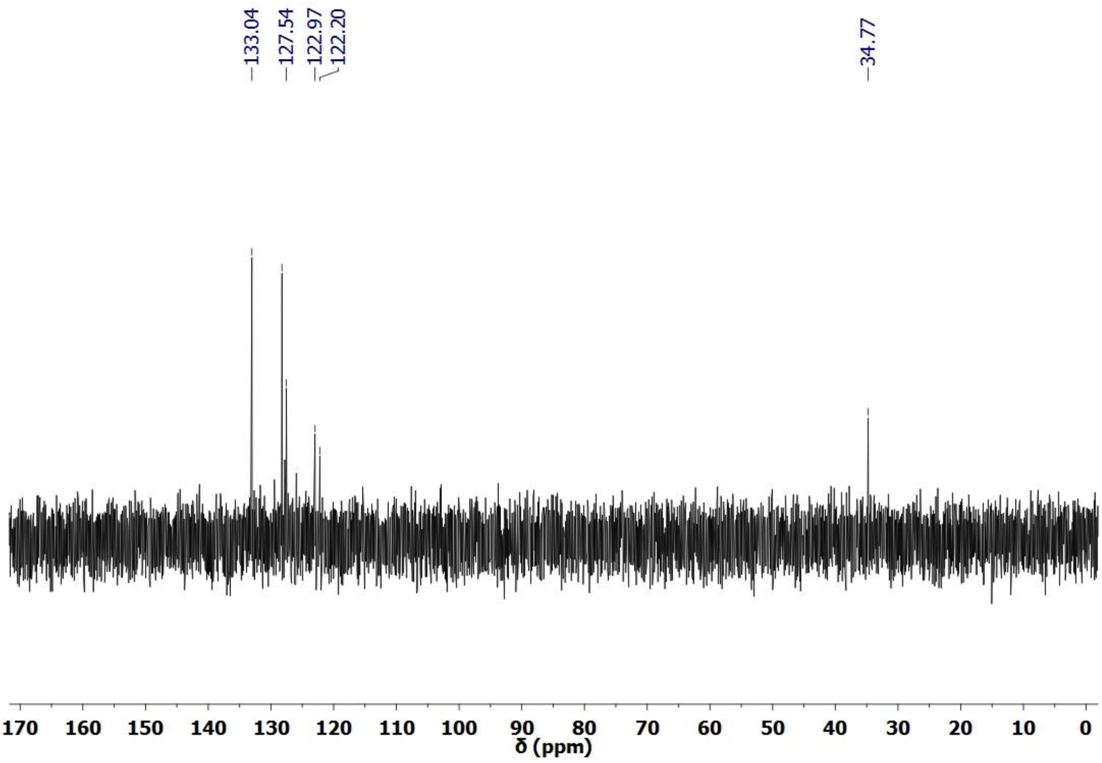

Supplementary Figure 4 | ^{13}C NMR (DEPT135) spectrum (C_6D_6) of **1**.

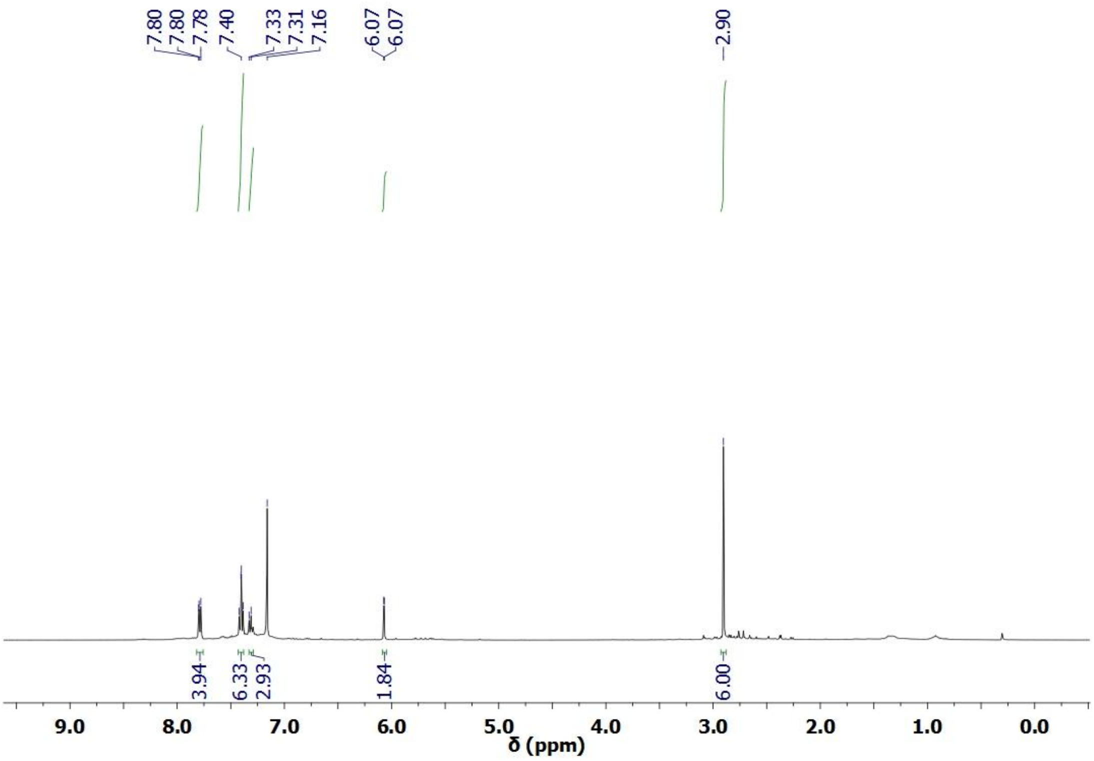

Supplementary Figure 5 | ^{1}H NMR spectrum (C_6D_6) of **2**.

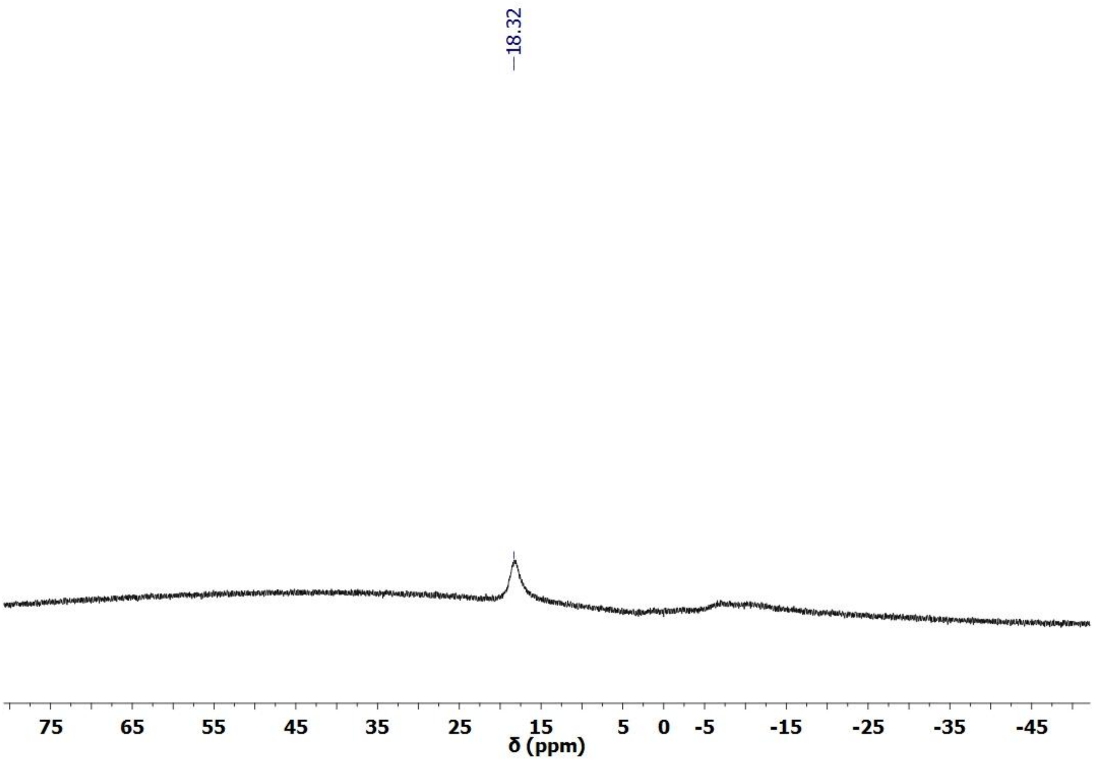

Supplementary Figure 6 | $^{11}B\{^{1}H\}$ NMR spectrum (C_6D_6) of **2**.

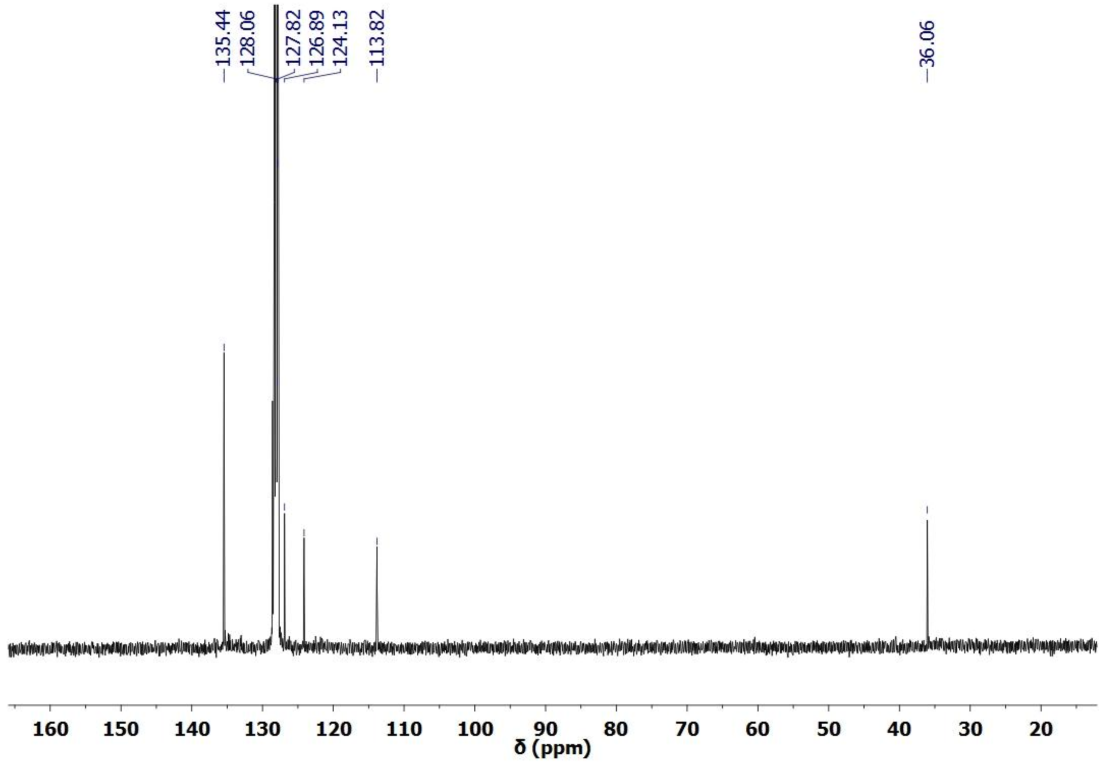

Supplementary Figure 7 | ^{13}C NMR spectrum (C_6D_6) of **2**.

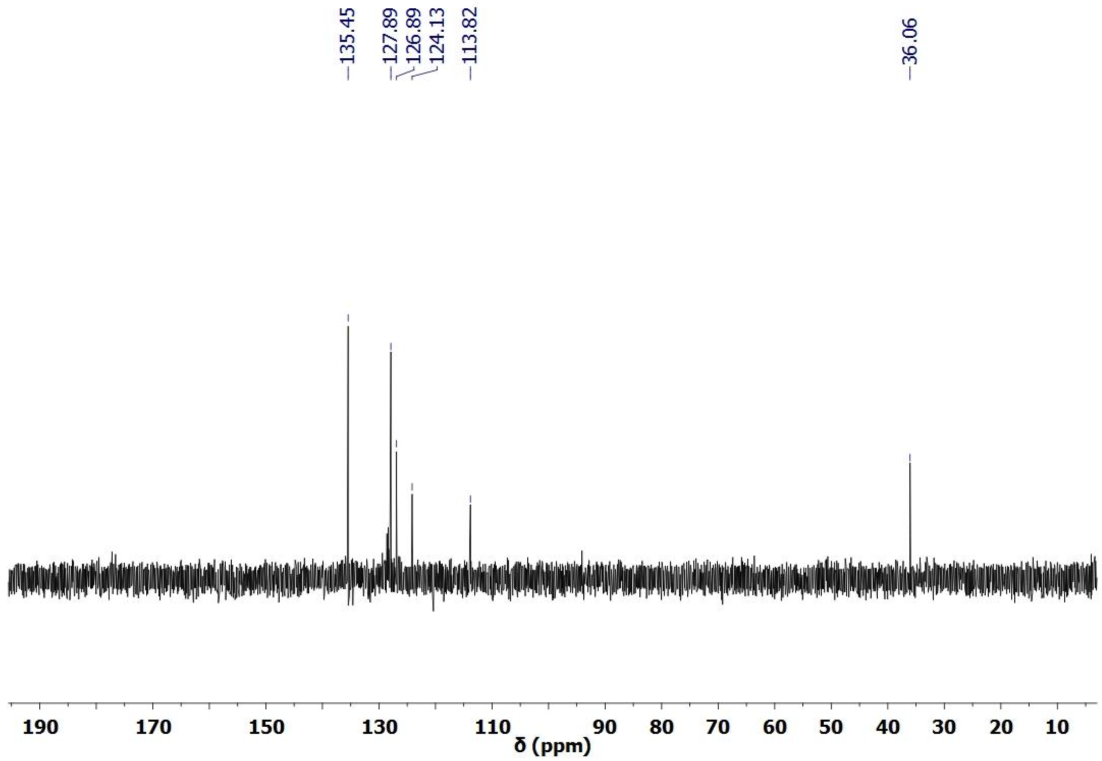

Supplementary Figure 8 | ^{13}C NMR (DEPT135) spectrum (C_6D_6) of **2**.

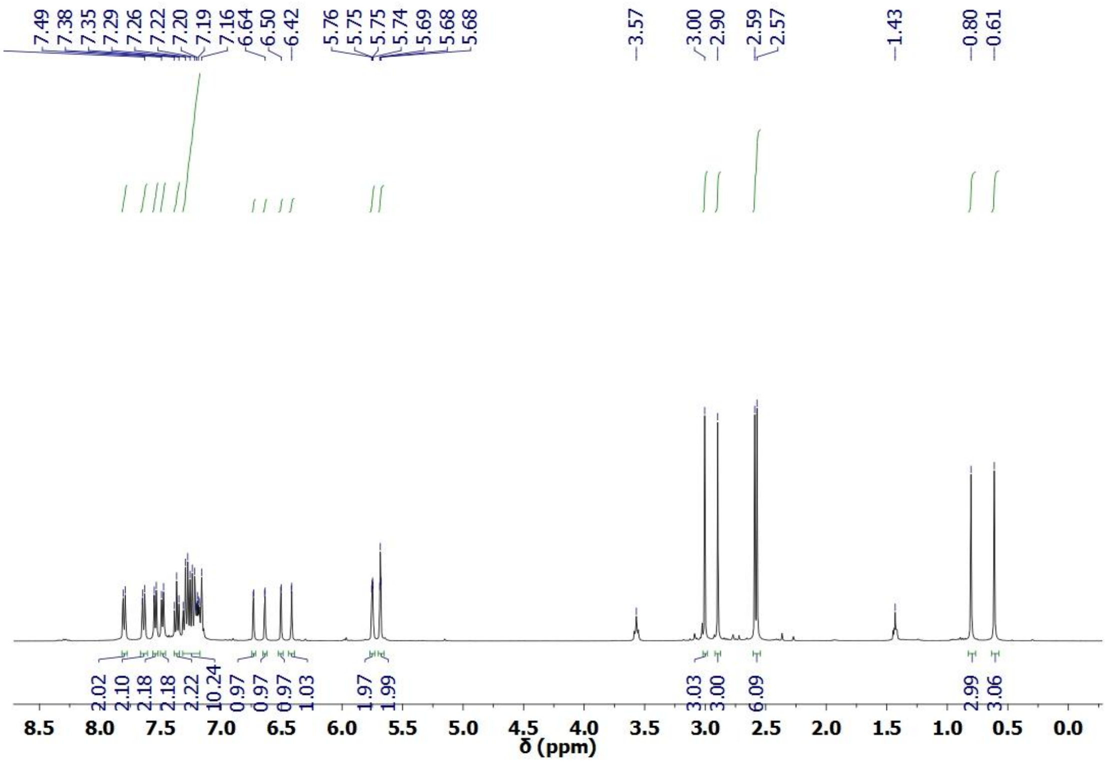

Supplementary Figure 9 | ^{1}H NMR spectrum (C_6D_6) of **3**.

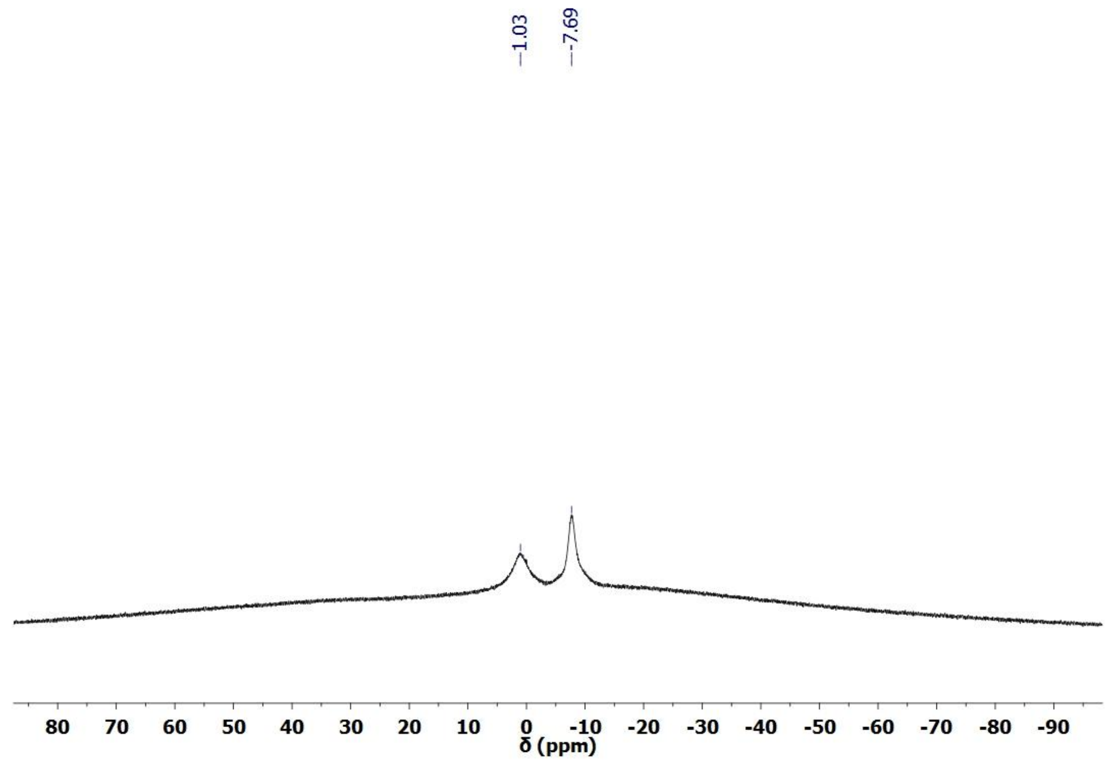

Supplementary Figure 10 | $^{11}B\{^{1}H\}$ NMR spectrum (C_6D_6) of **3**.

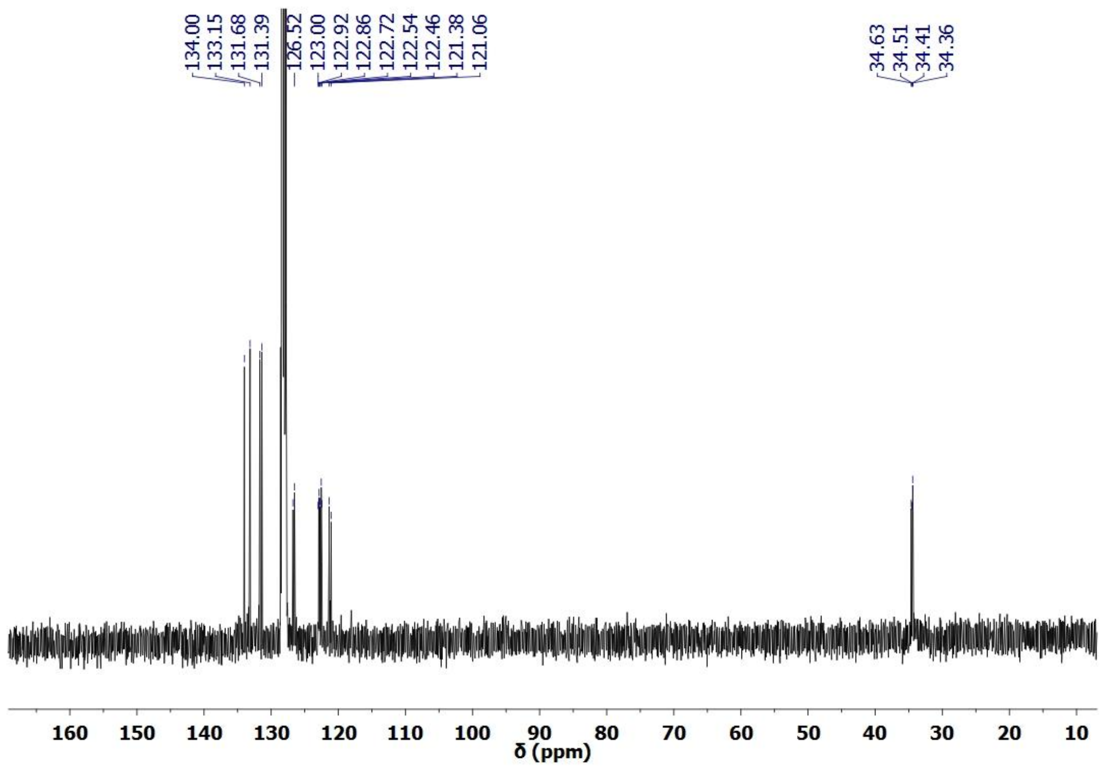

Supplementary Figure 11 | ^{13}C NMR spectrum (C_6D_6) of **3**.

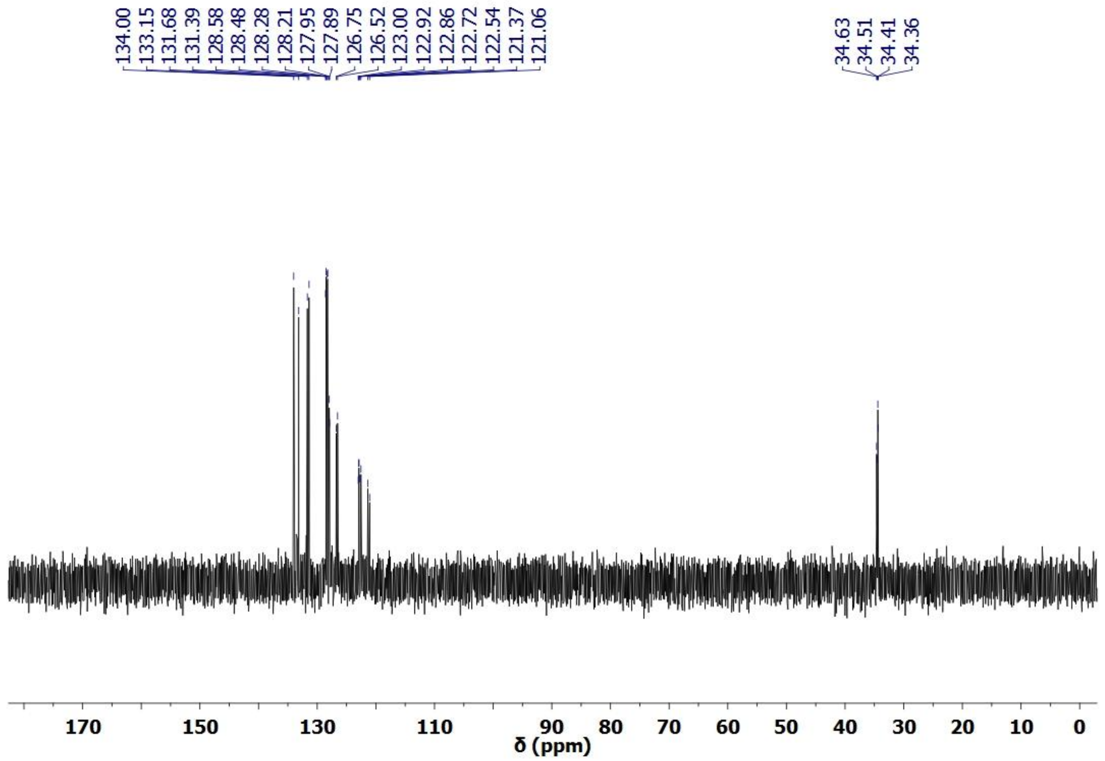

Supplementary Figure 12 | ^{13}C NMR (DEPT135) spectrum (C_6D_6) of **3**.

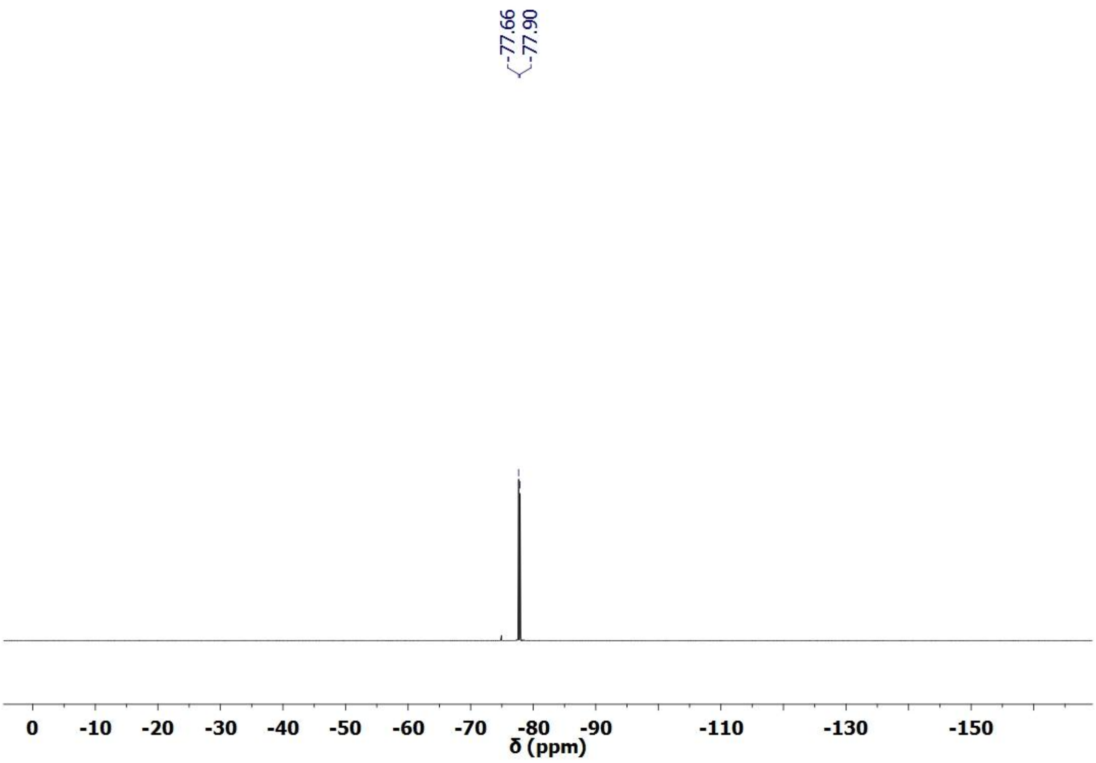

Supplementary Figure 13 | ^{19}F NMR spectrum (C_6D_6) of **3**.

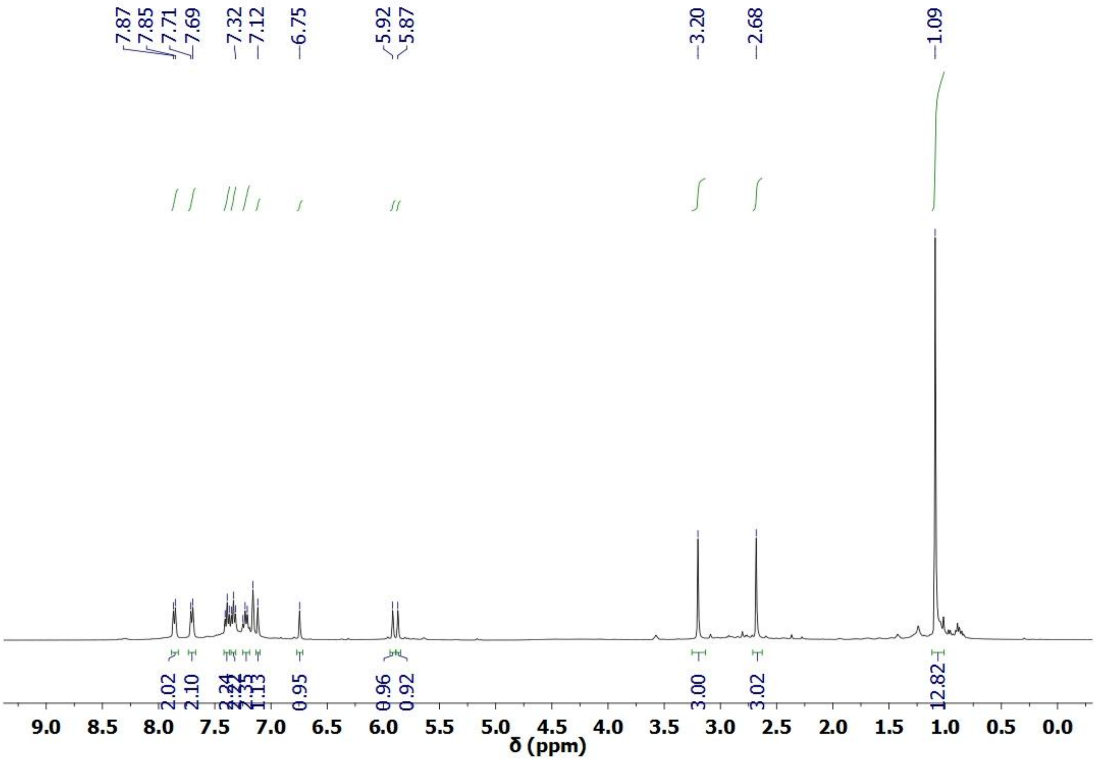

Supplementary Figure 14 | ^{1}H NMR spectrum (C_6D_6) of **4**.

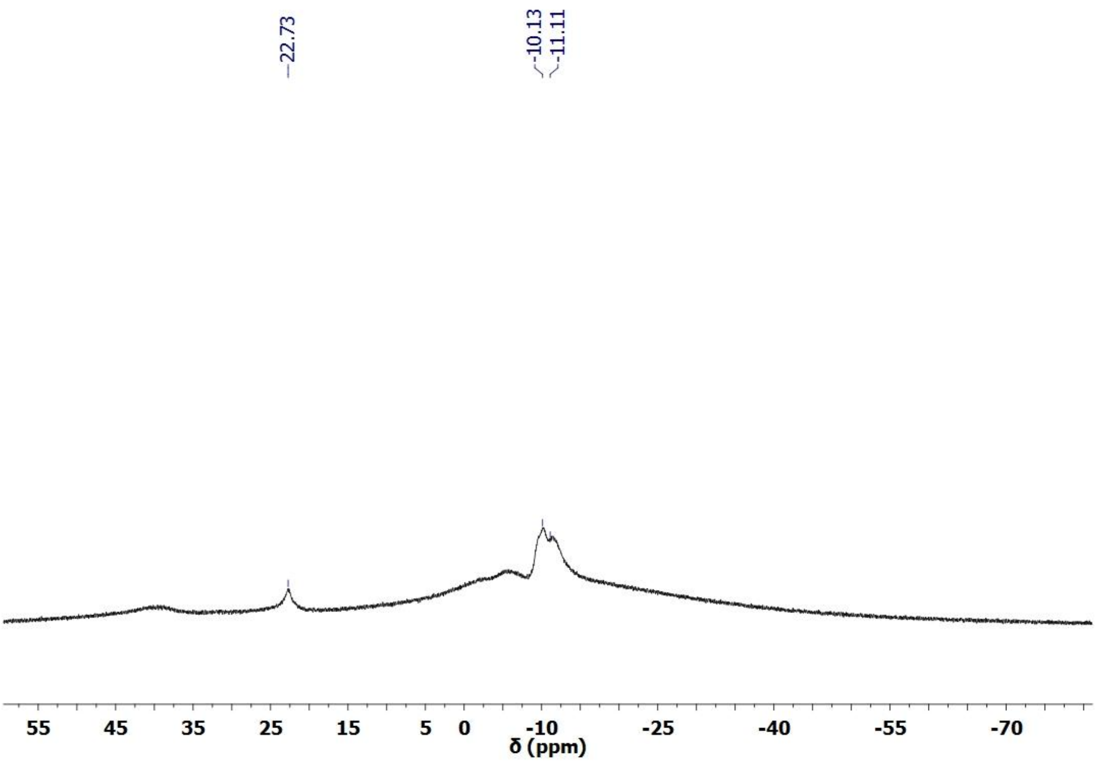

Supplementary Figure 15 | $^{11}B\{^{1}H\}$ NMR spectrum (C_6D_6) of **4**.

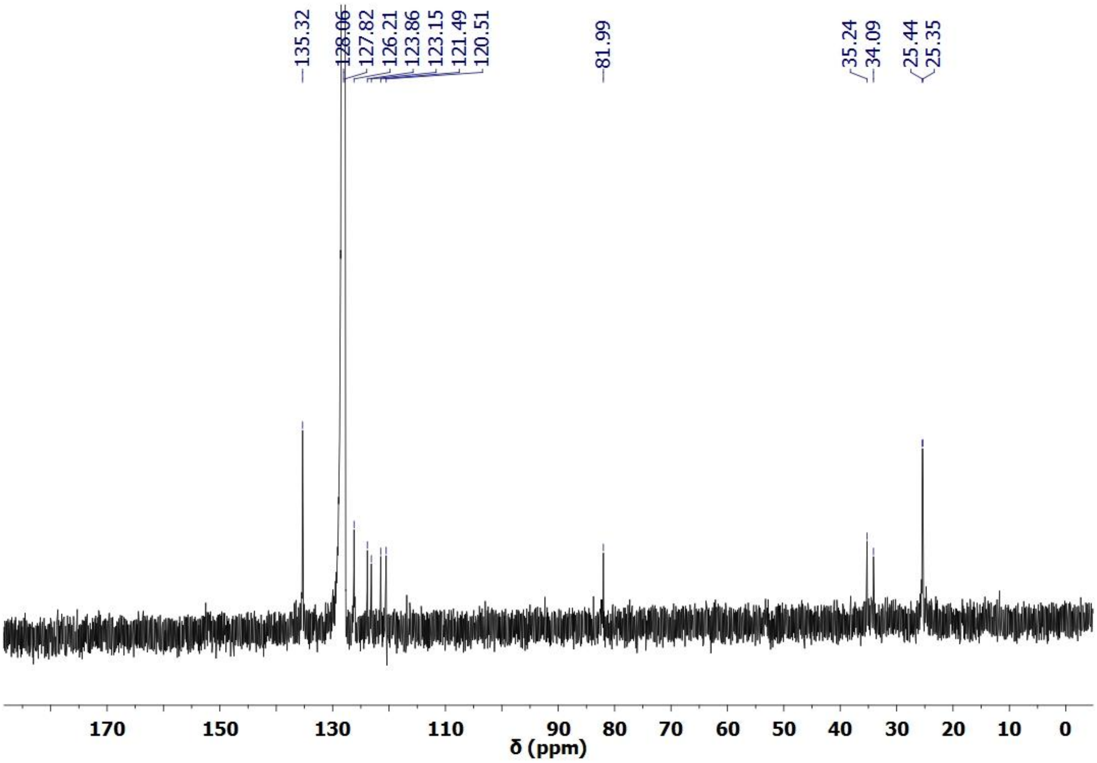

Supplementary Figure 16 | ^{13}C NMR spectrum (C_6D_6) of **4**.

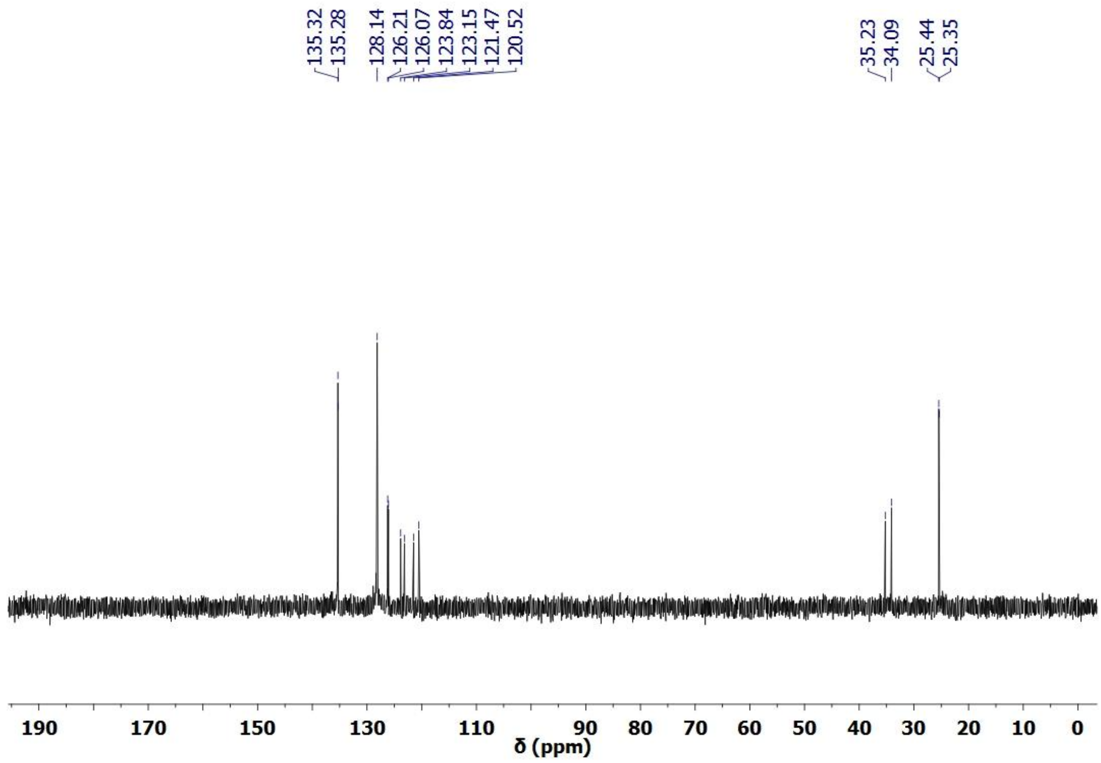

Supplementary Figure 17 | ^{13}C NMR (DEPT135) spectrum (C_6D_6) of **4**.

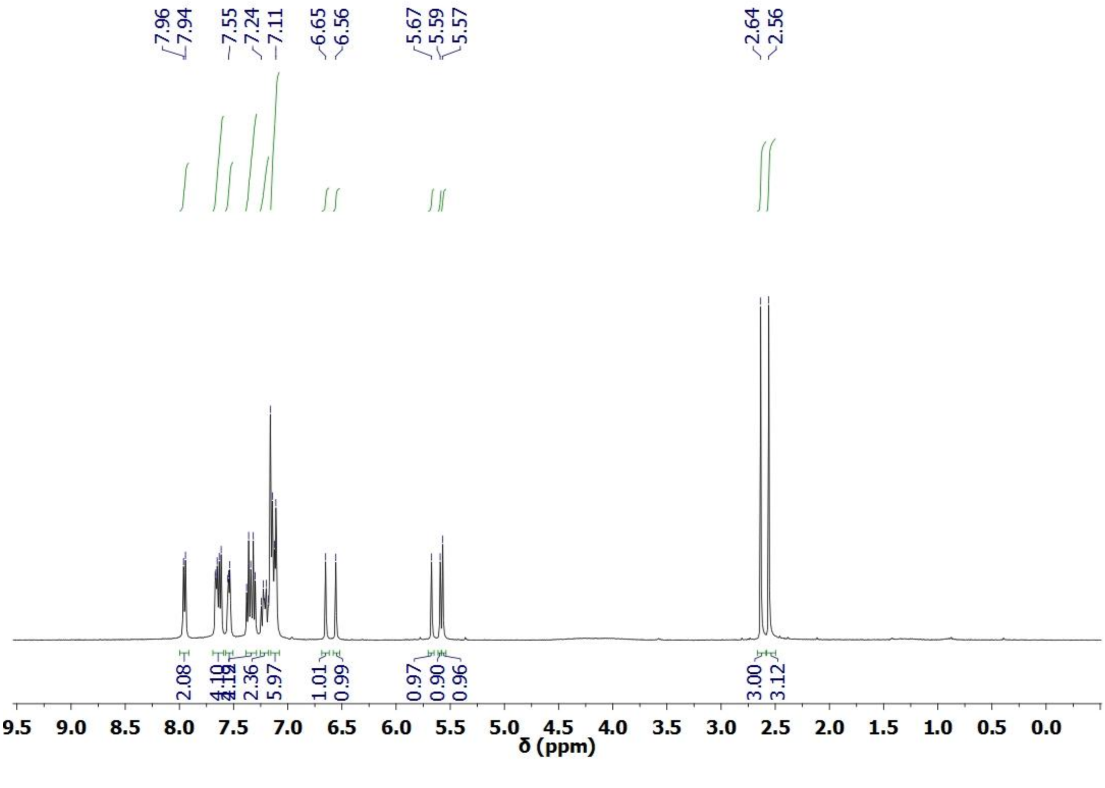

Supplementary Figure 18 | ^{1}H NMR spectrum (C_6D_6) of **5a**.

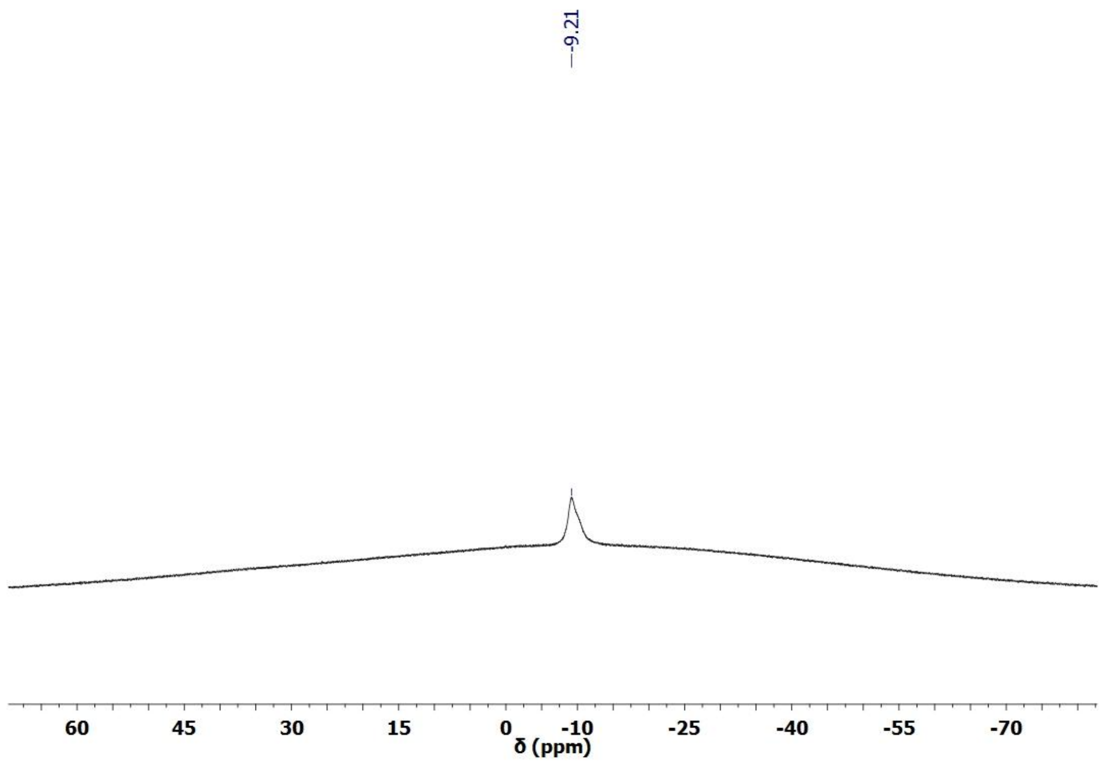

Supplementary Figure 19 | $^{11}B\{^{1}H\}$ NMR spectrum (C_6D_6) of **5a**.

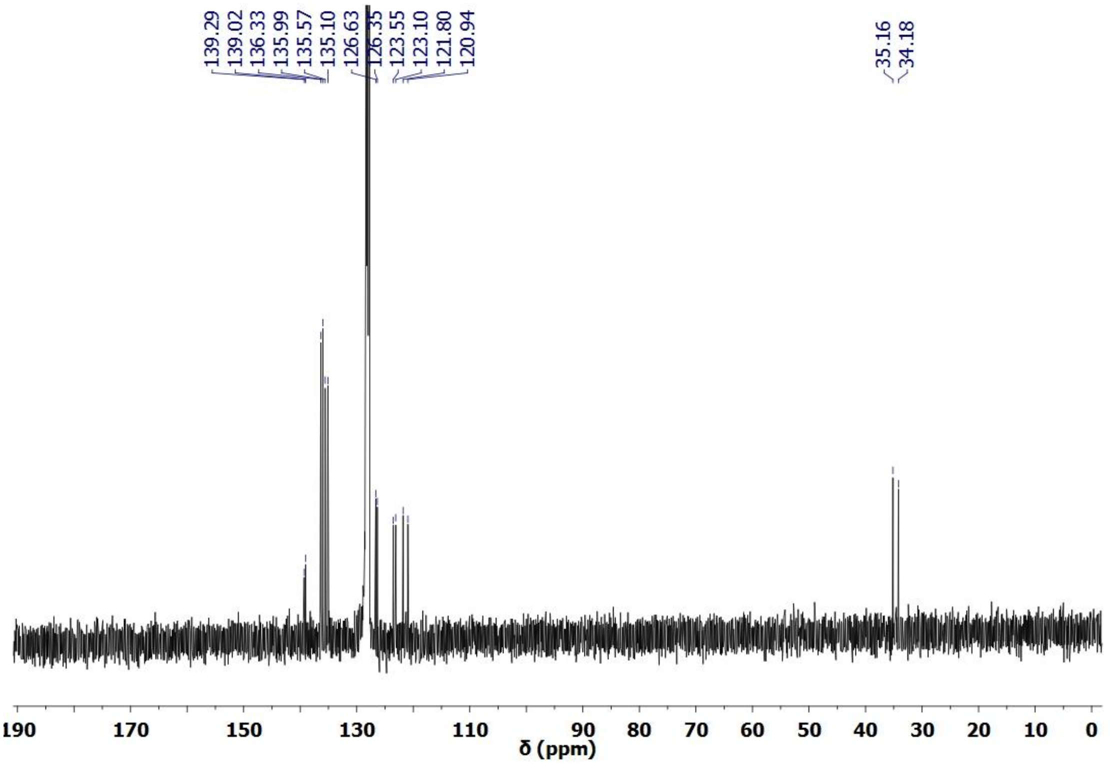

Supplementary Figure 20 | ^{13}C NMR spectrum (C_6D_6) of **5a**.

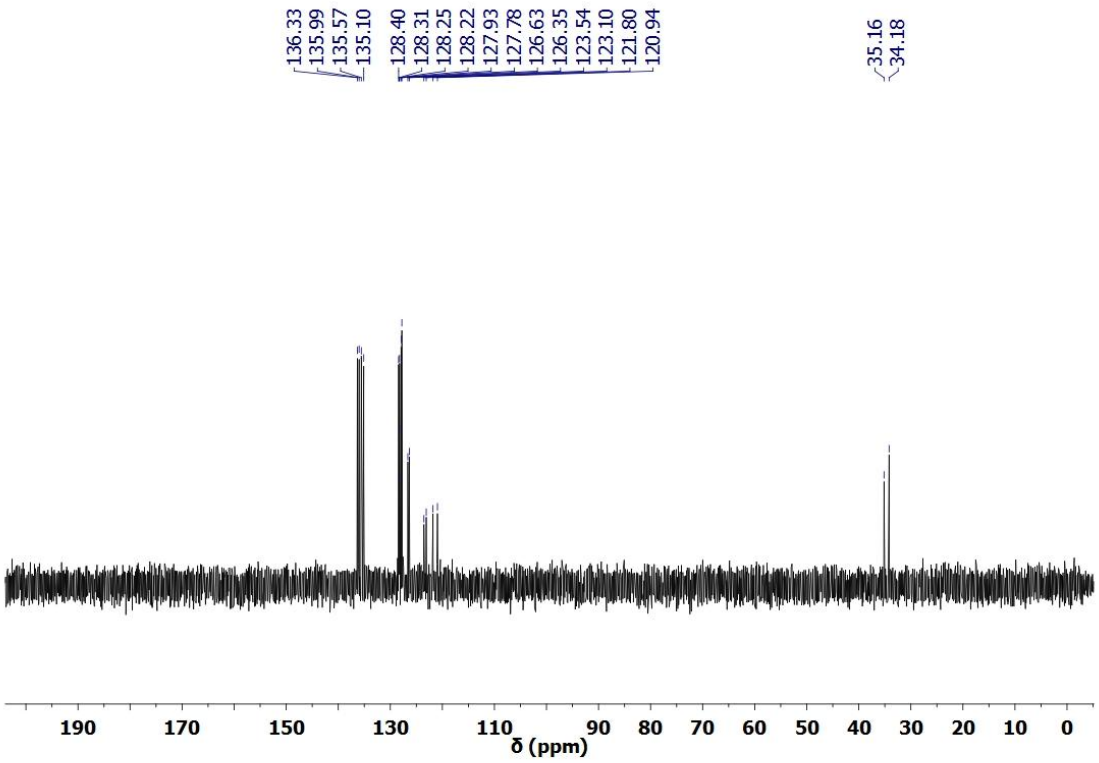

Supplementary Figure 21 | ^{13}C NMR (DEPT135) spectrum (C_6D_6) of **5a**.

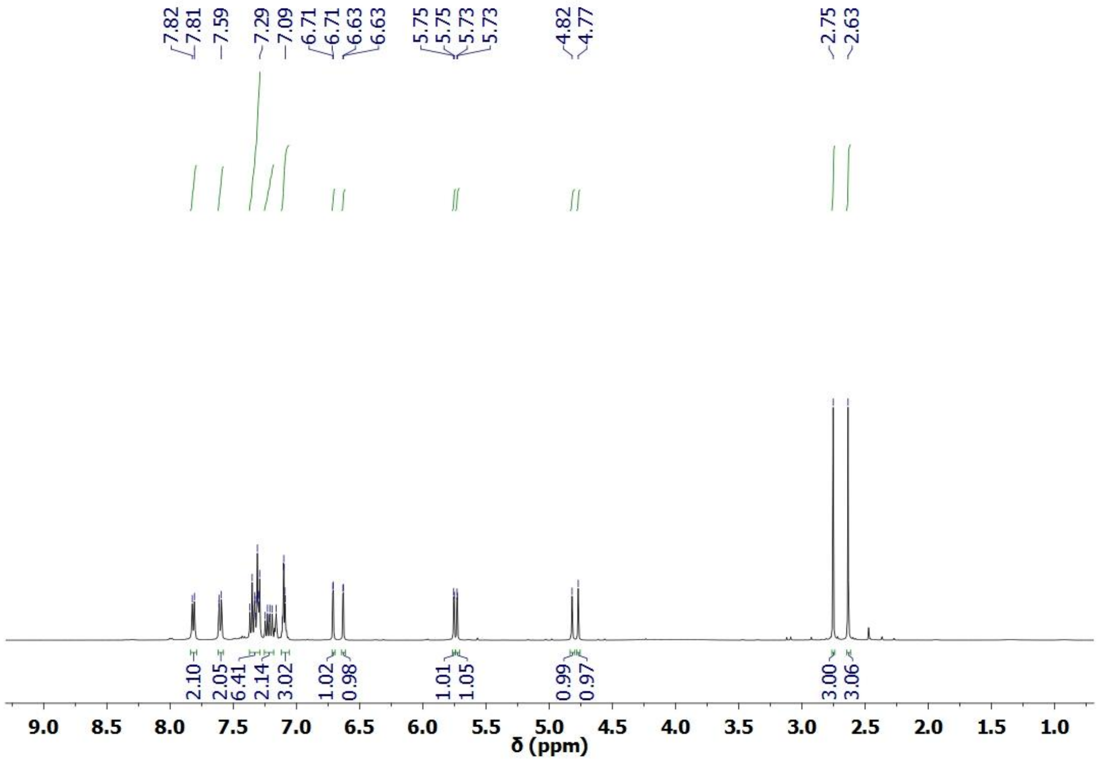

Supplementary Figure 22 | ^{1}H NMR spectrum (C_6D_6) of **5b**.

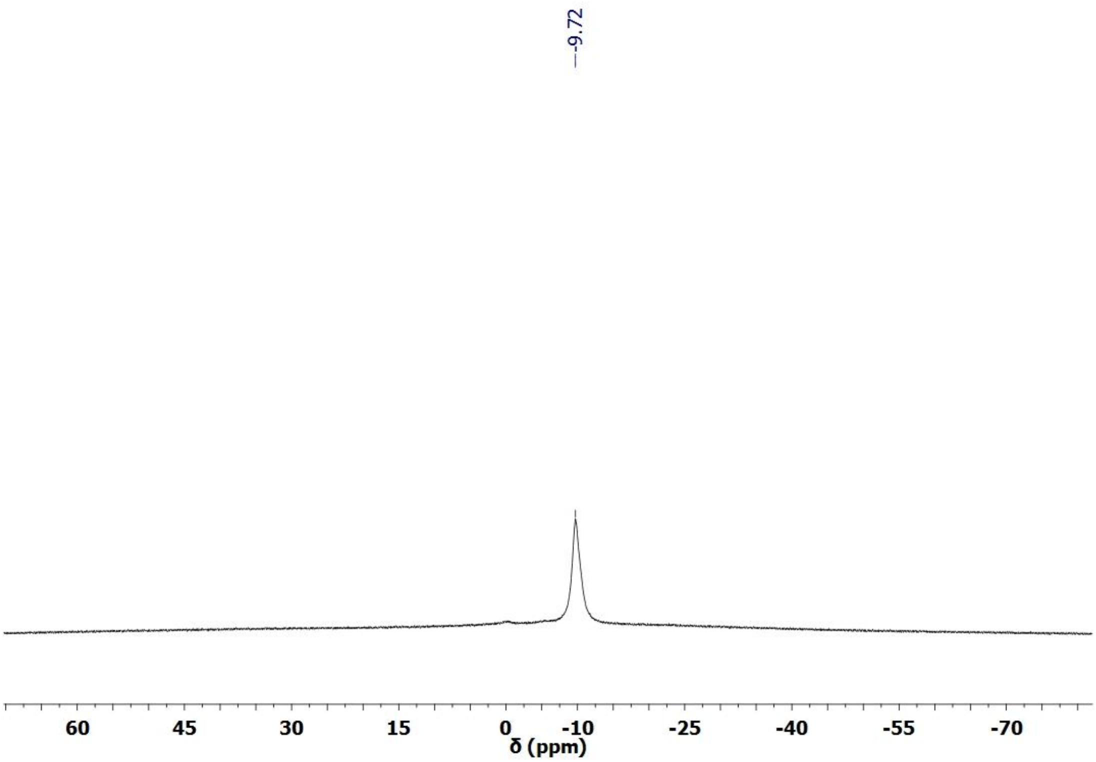

Supplementary Figure 23 | $^{11}B\{^{1}H\}$ NMR spectrum (C_6D_6) of **5b**.

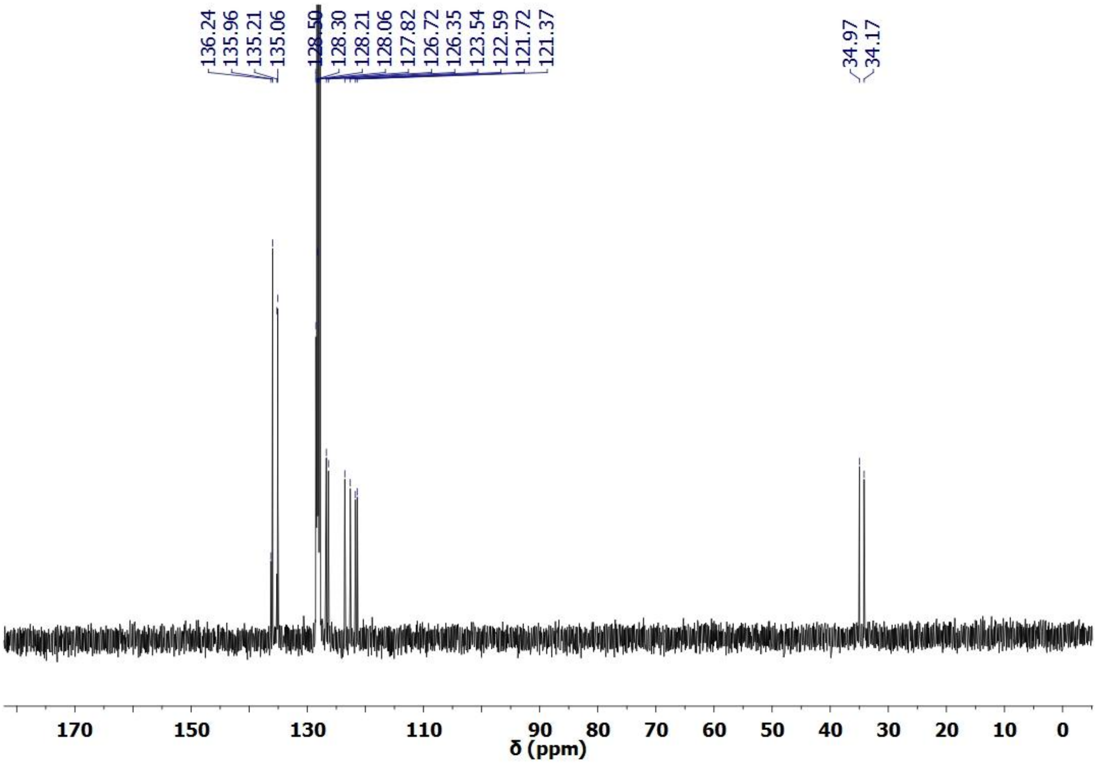

Supplementary Figure 24 | ^{13}C NMR spectrum (C_6D_6) of **5b**.

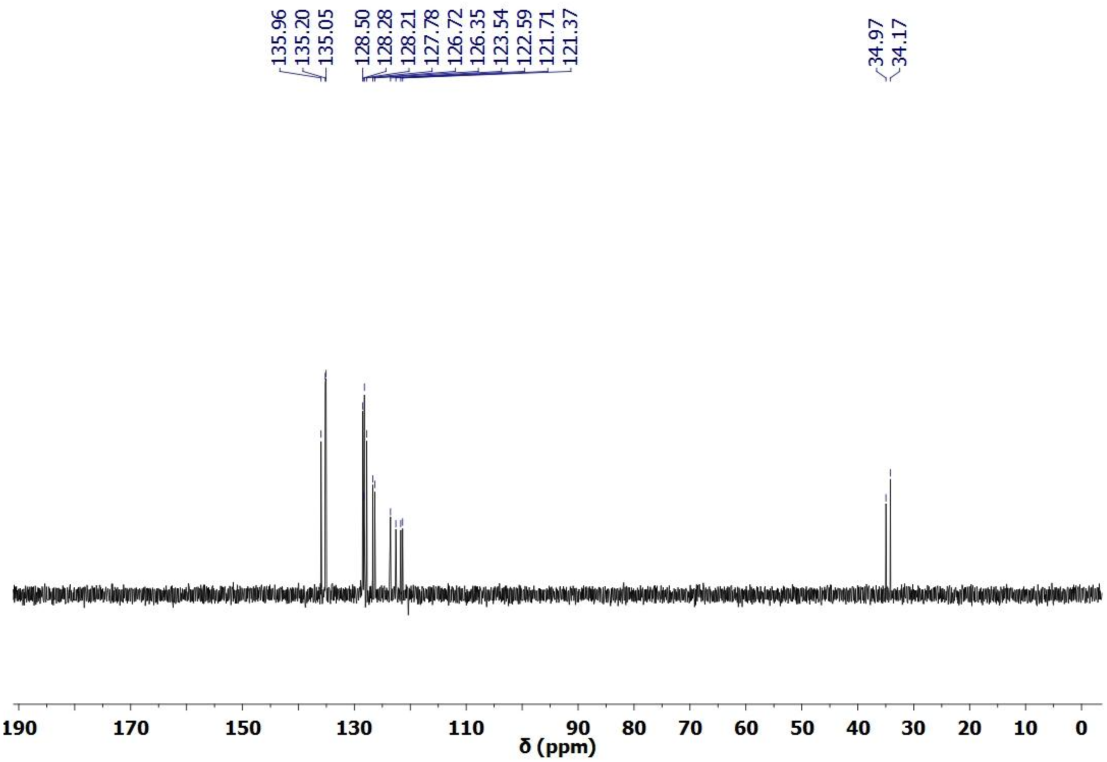

Supplementary Figure 25 | ^{13}C NMR (DEPT135) spectrum (C_6D_6) of **5b**.

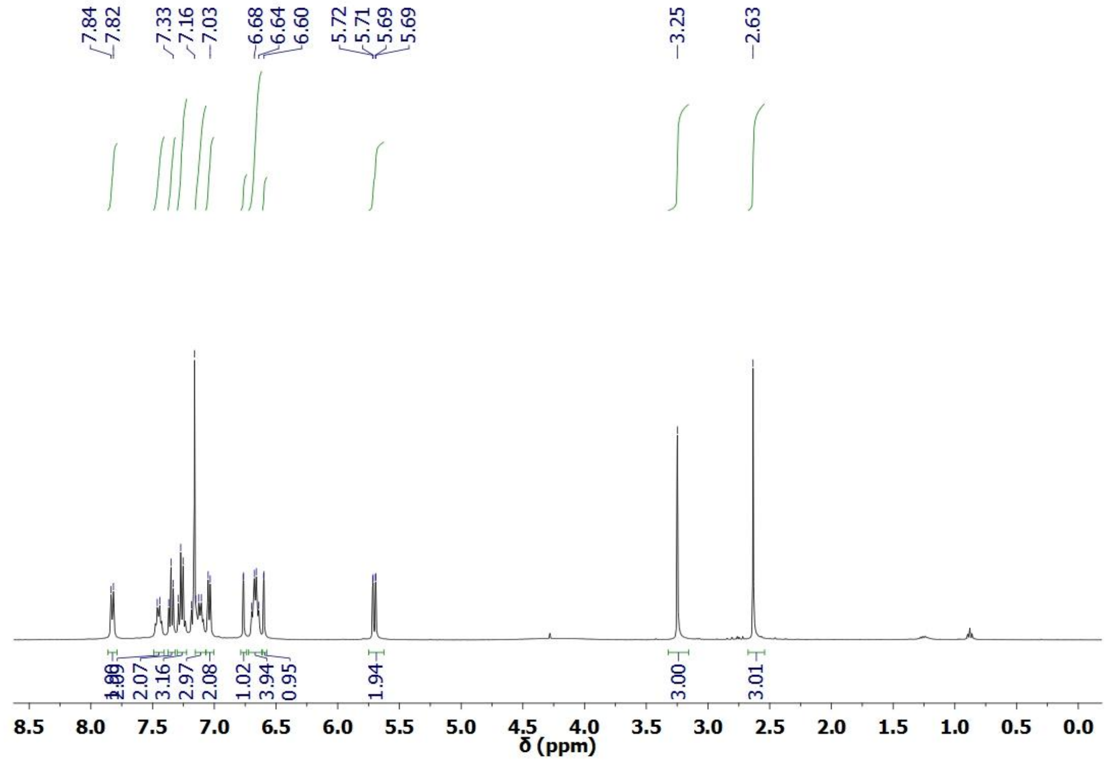

Supplementary Figure 26 | ^{1}H NMR spectrum (C_6D_6) of **6**.

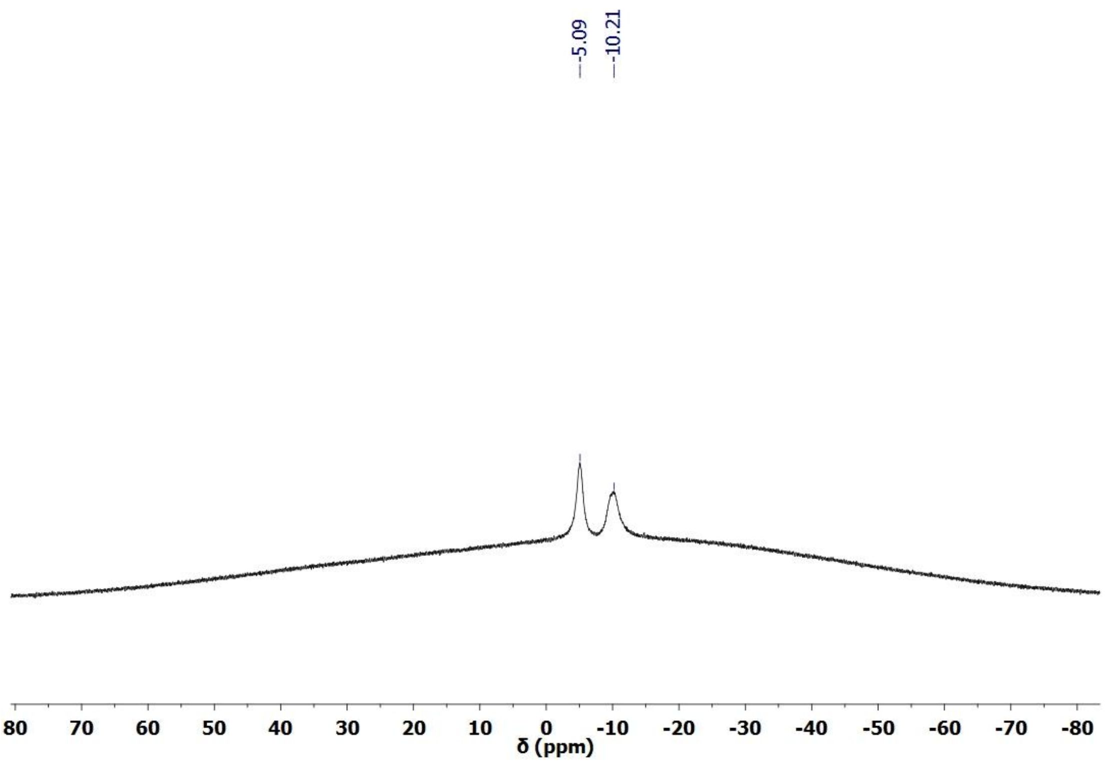

Supplementary Figure 27 | $^{11}B\{^{1}H\}$ NMR spectrum (C_6D_6) of **6**.

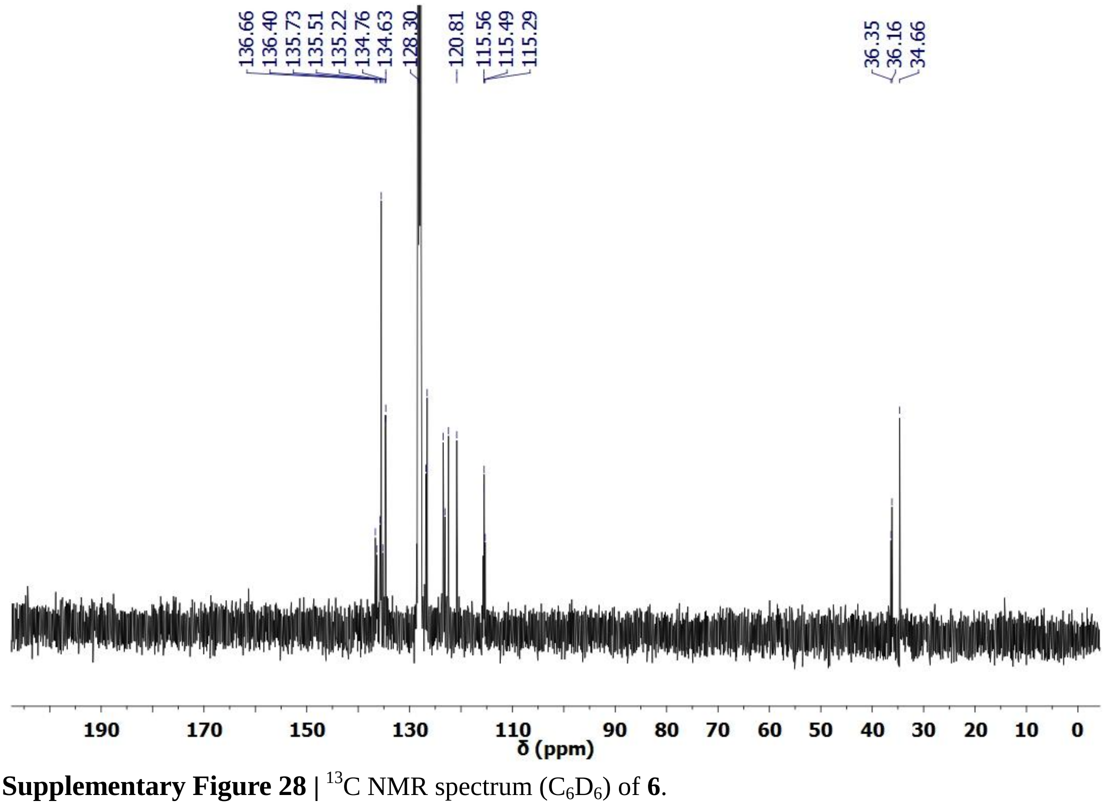

Supplementary Figure 28 | ^{13}C NMR spectrum (C_6D_6) of **6**.

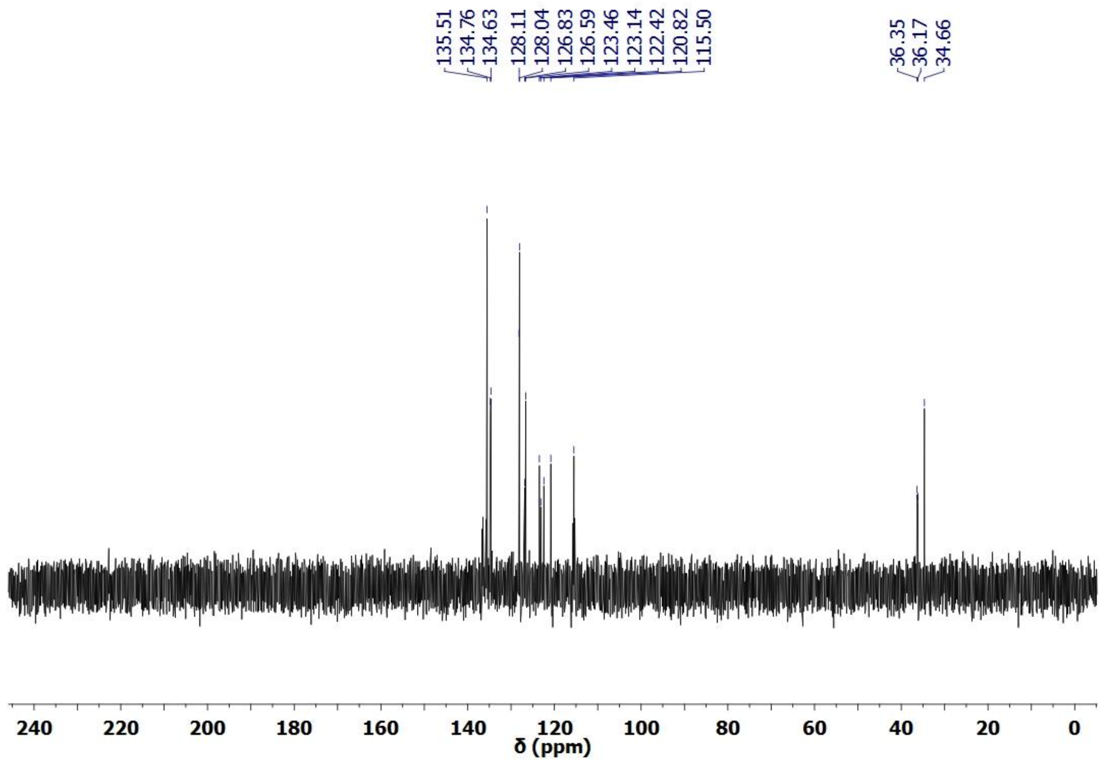

Supplementary Figure 29 | ^{13}C NMR (DEPT135) spectrum (C_6D_6) of **6**.

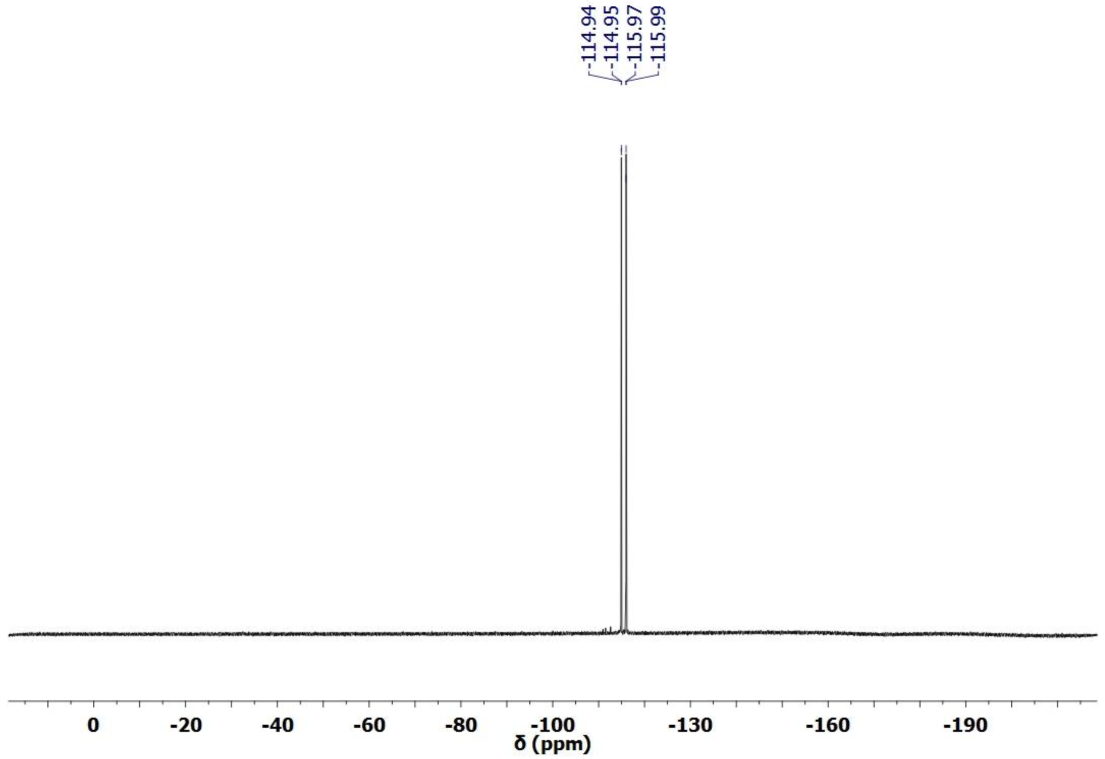

Supplementary Figure 30 | ^{19}F NMR spectrum (C_6D_6) of **6**.

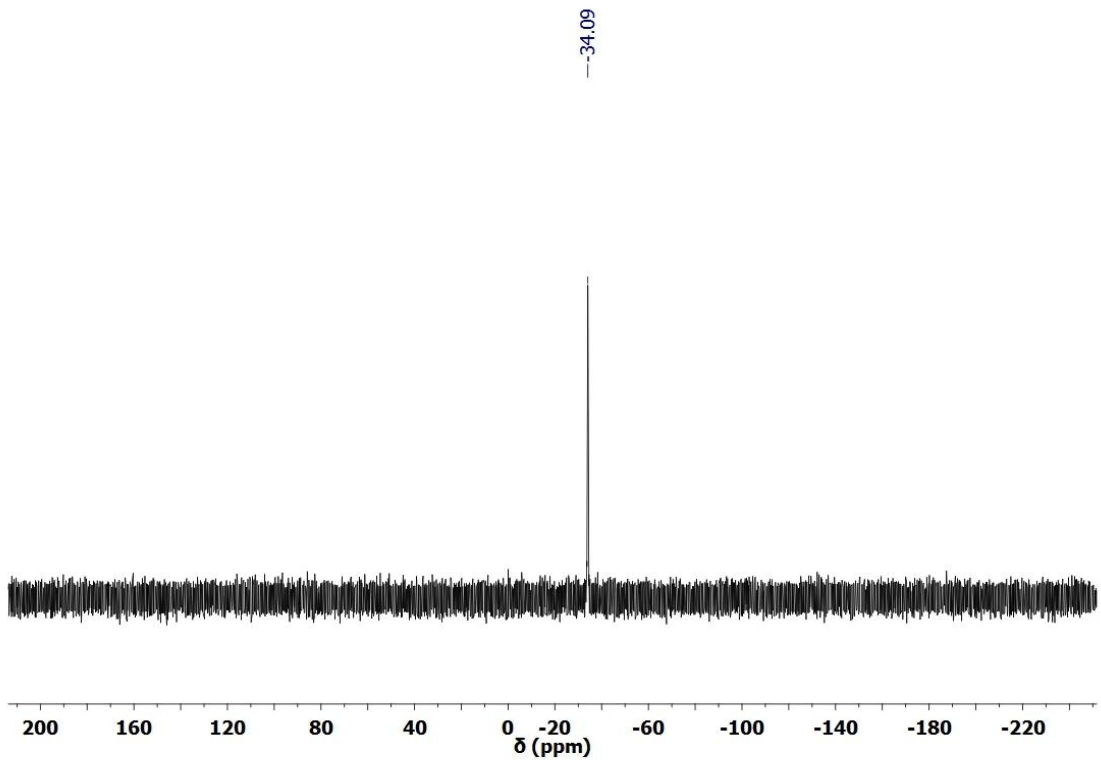

Supplementary Figure 31 | ^{31}P NMR spectrum (C_6D_6) of **6**.

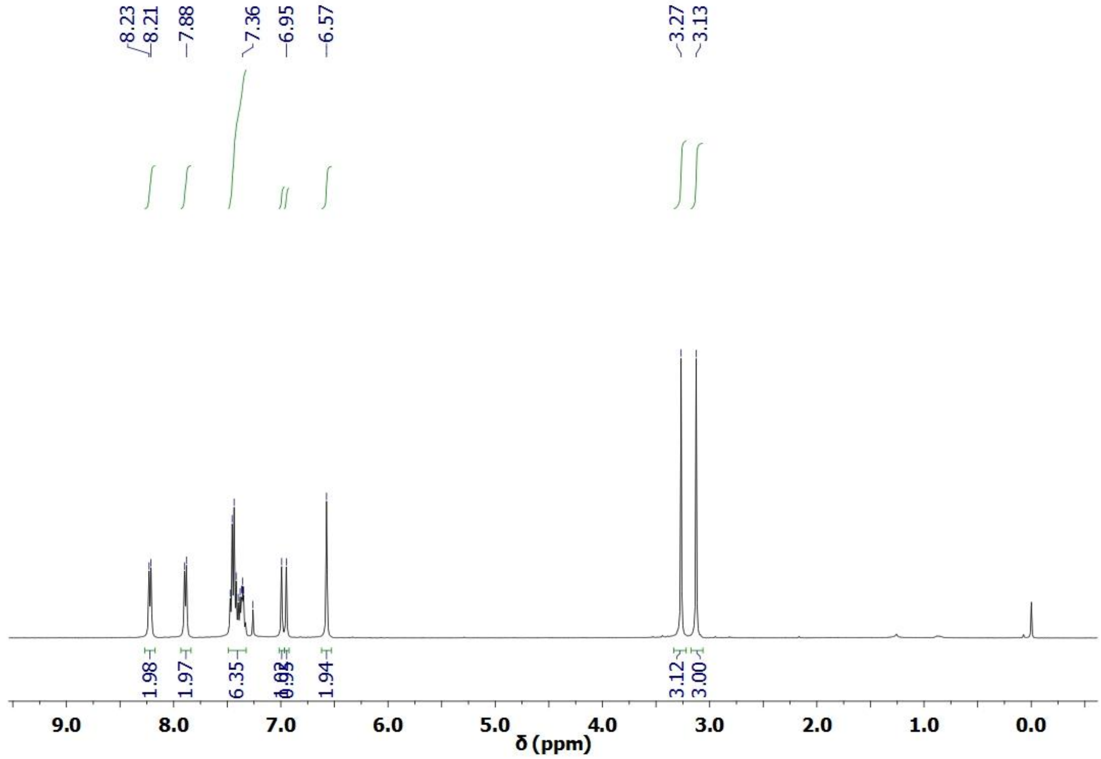

Supplementary Figure 32 | ^{1}H NMR spectrum ($CDCl_3$) of **7**.

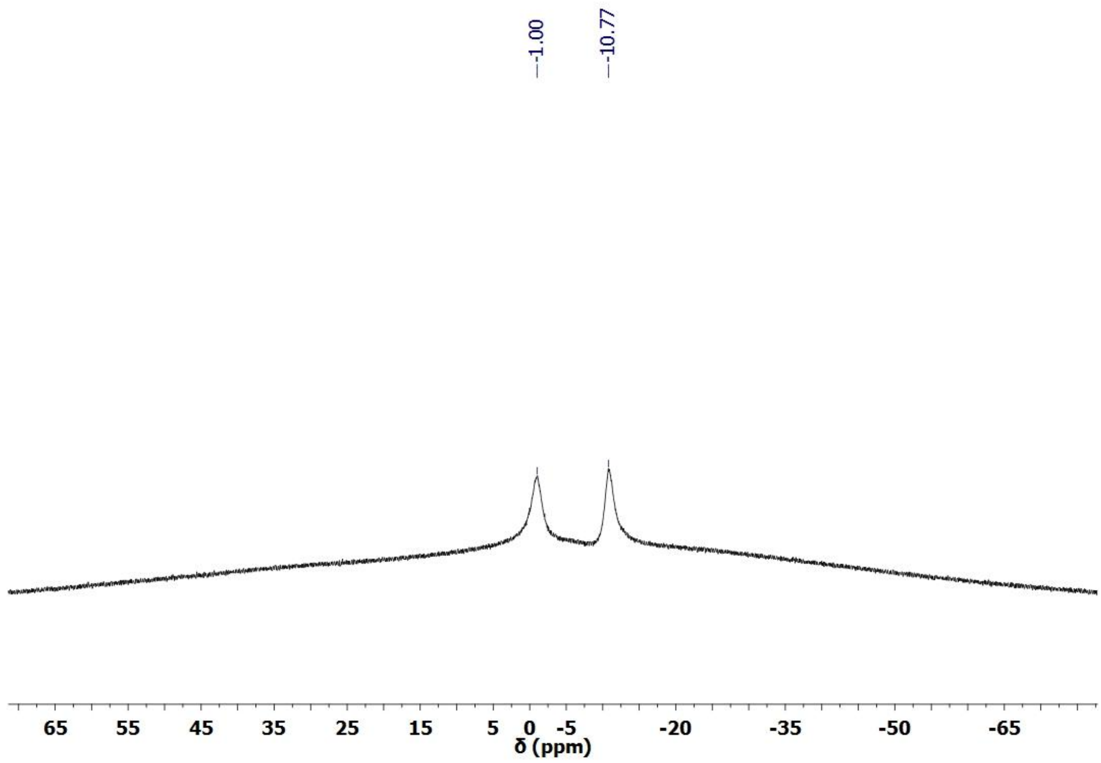

Supplementary Figure 33 | $^{11}B\{^1H\}$ NMR spectrum ($CDCl_3$) of **7**.

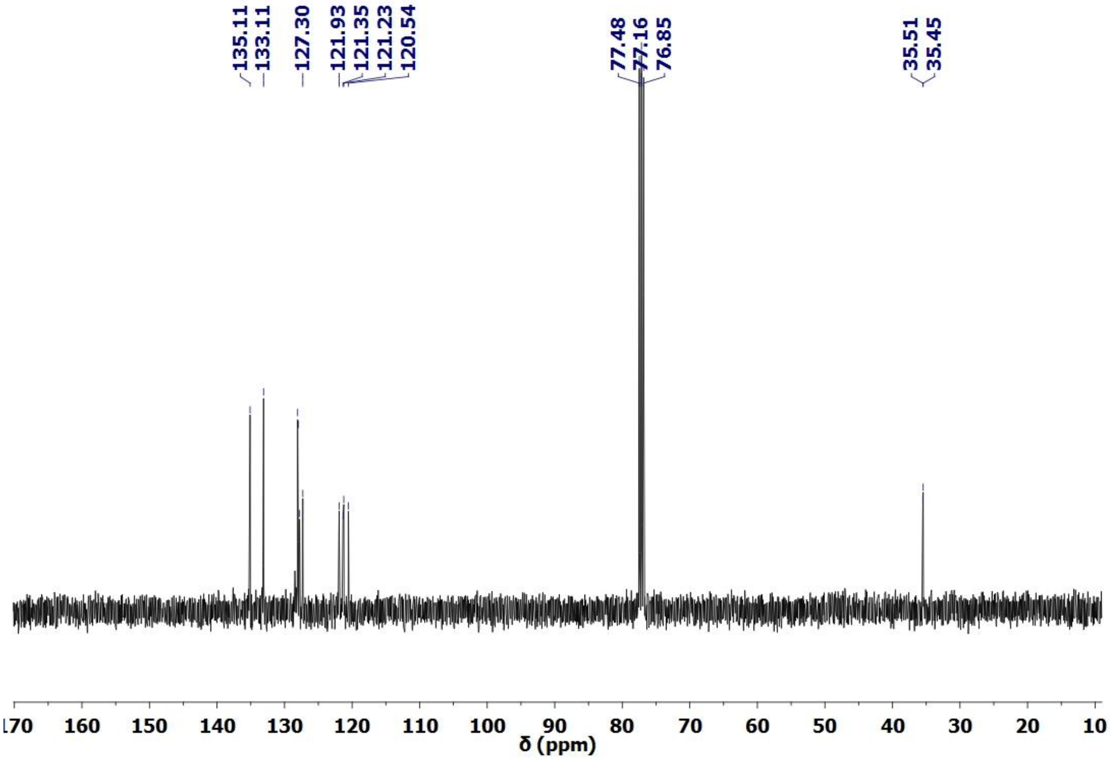

Supplementary Figure 34 | ^{11}C NMR spectrum ($CDCl_3$) of **7**.

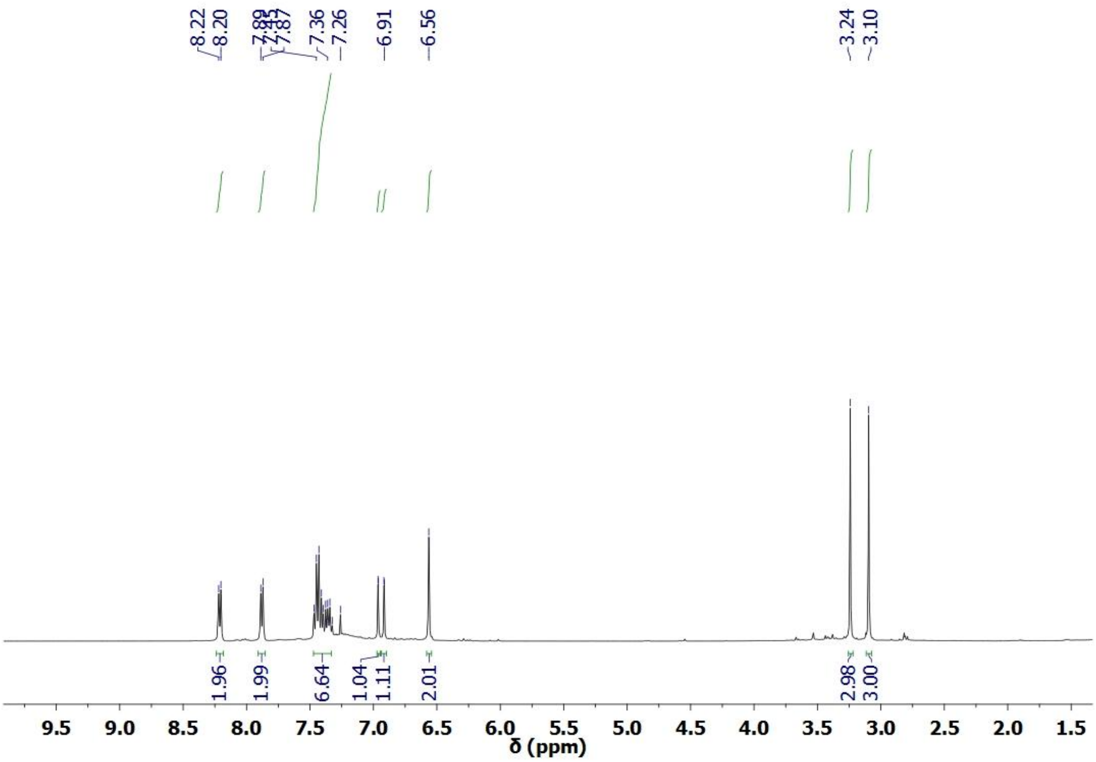

Supplementary Figure 35 | ^{1}H NMR spectrum ($CDCl_3$) of **7-^{13}C**.

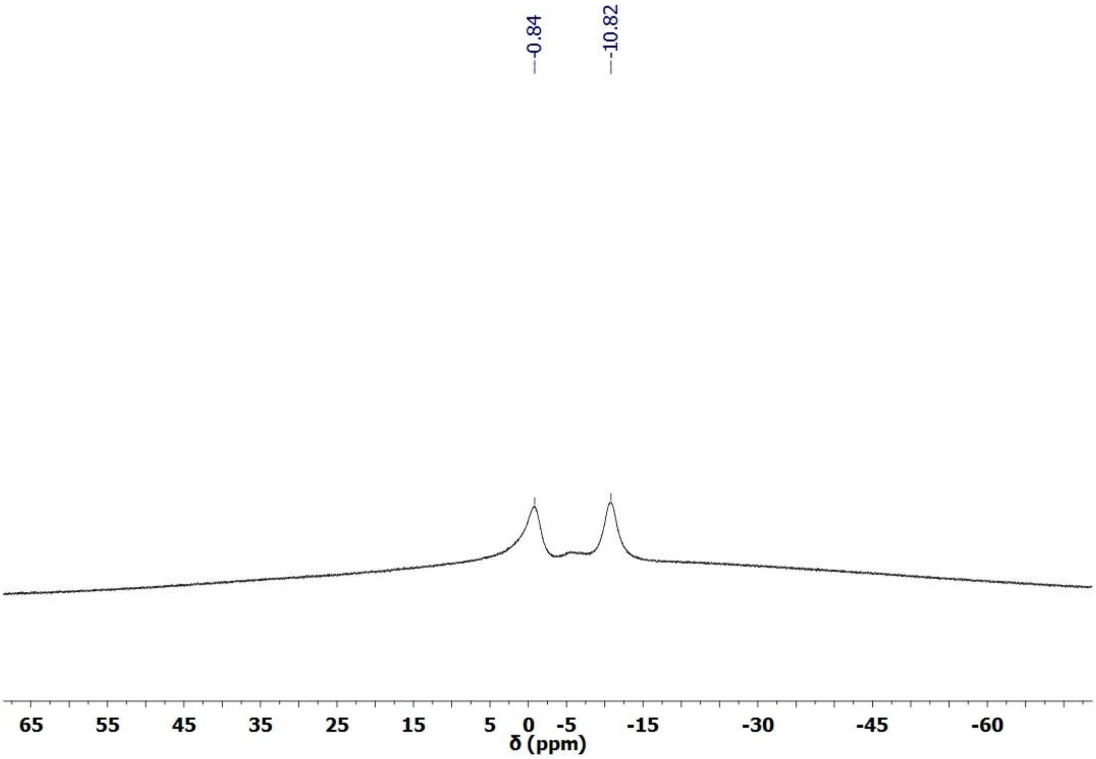

Supplementary Figure 36 | ^{11}B{^{1}H} NMR spectrum ($CDCl_3$) of **7-^{13}C**.

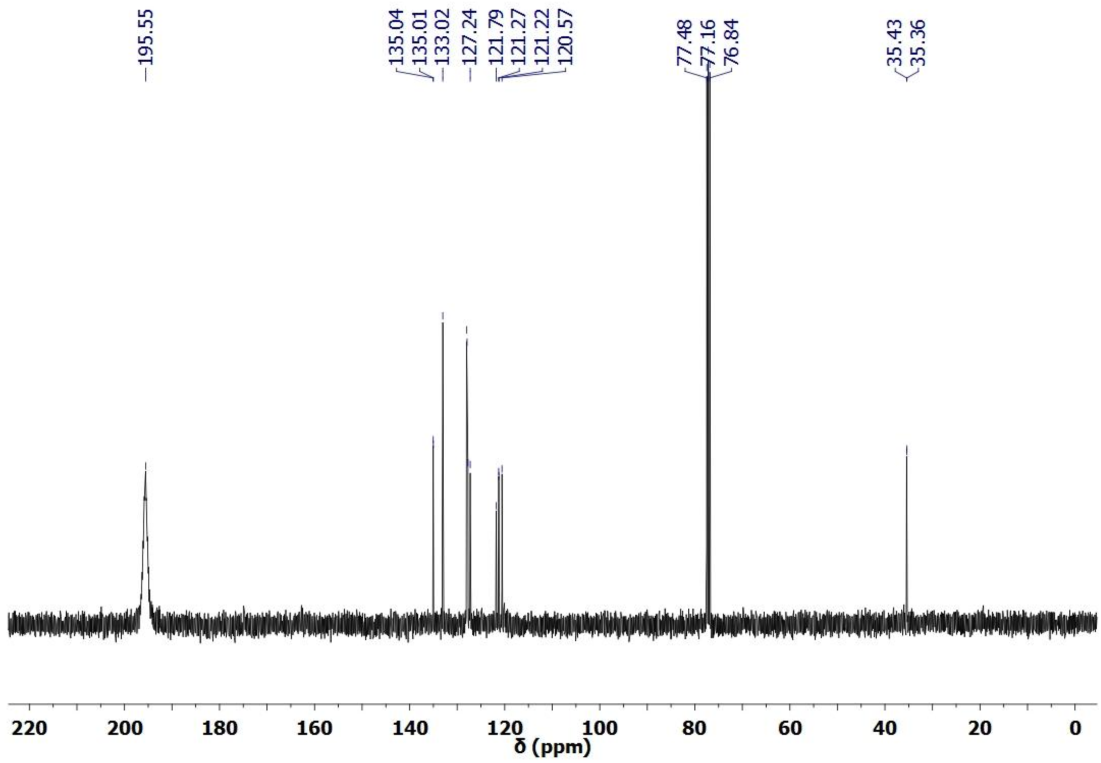

Supplementary Figure 37 | ^{13}C NMR spectrum ($CDCl_3$) of **7-^{13}C**.

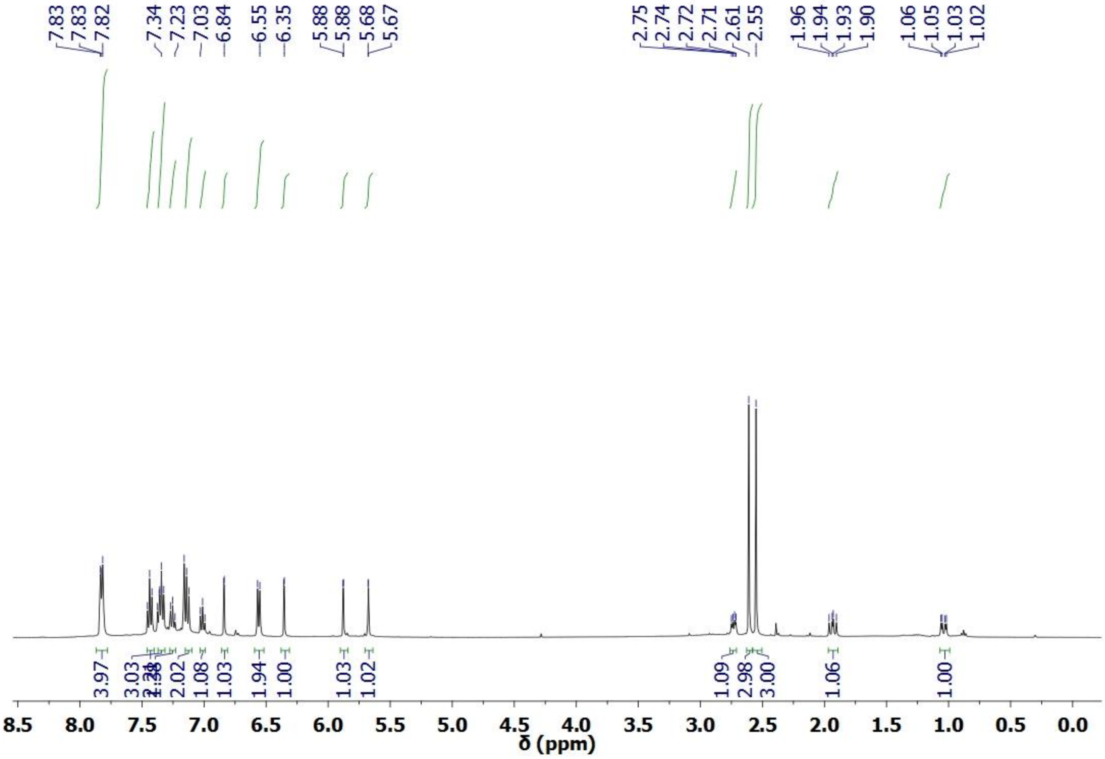

Supplementary Figure 38 | ^{1}H NMR spectrum (C_6D_6) of **8**.

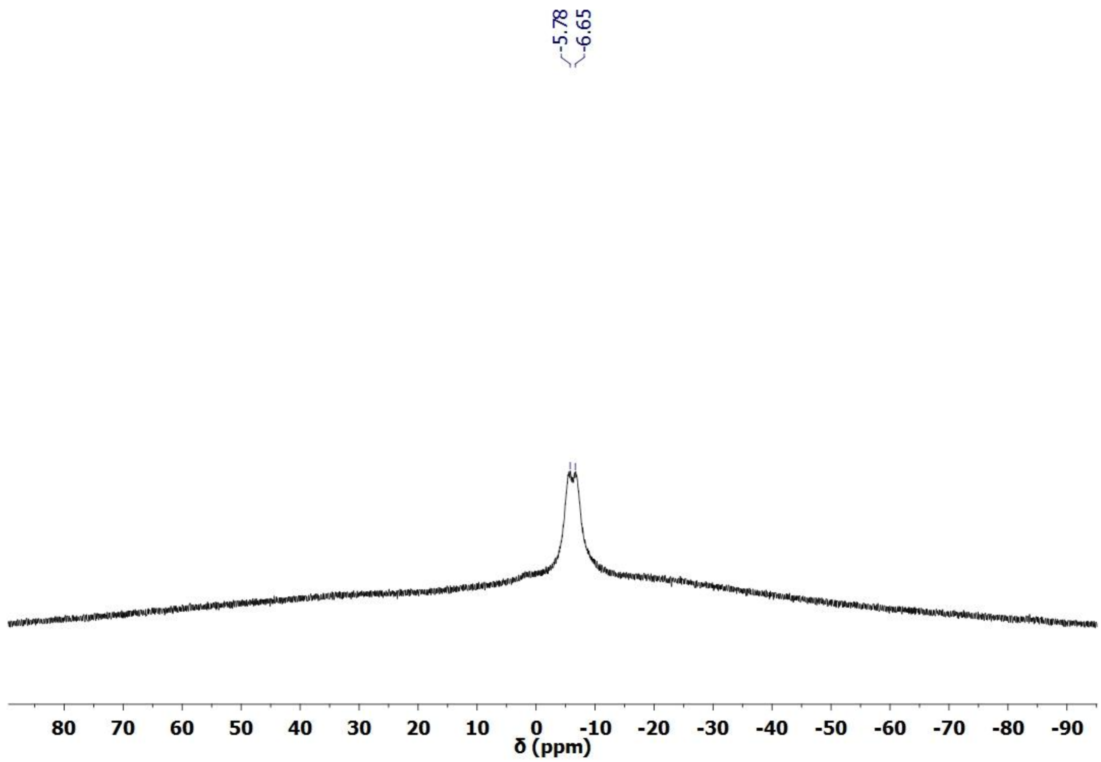

Supplementary Figure 39 | $^{11}B\{^{1}H\}$ NMR spectrum (C_6D_6) of **8**.

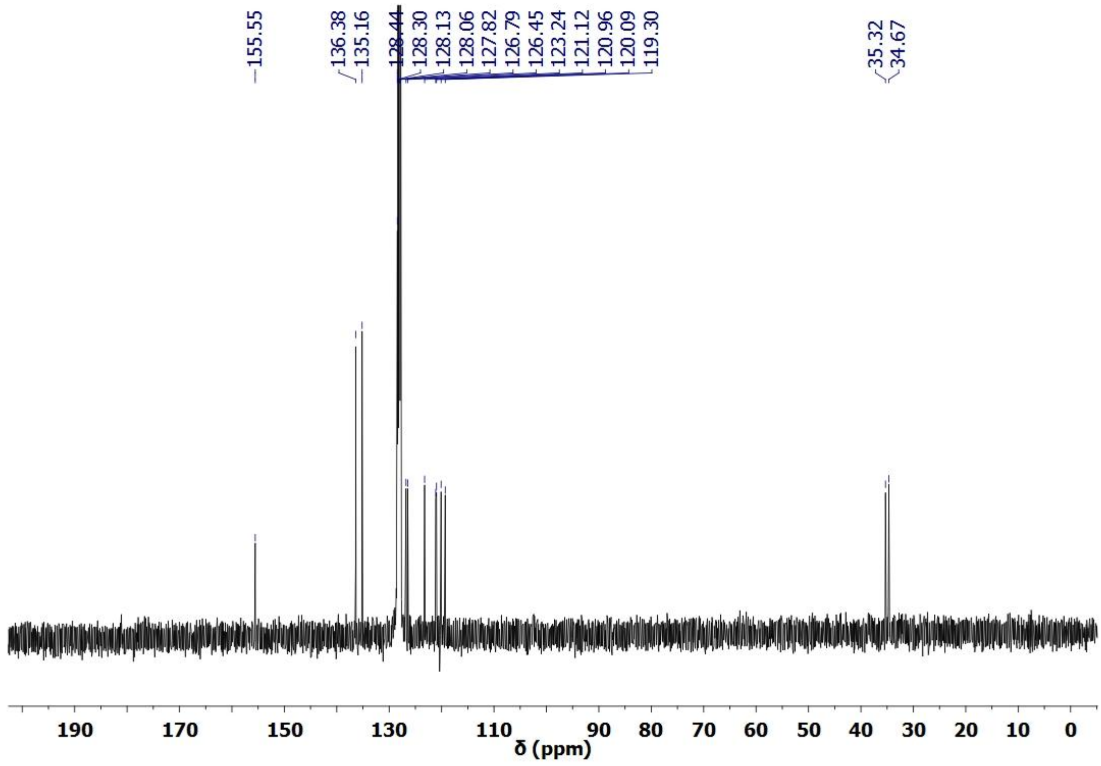

Supplementary Figure 40 | ^{13}C NMR spectrum (C_6D_6) of **8**.

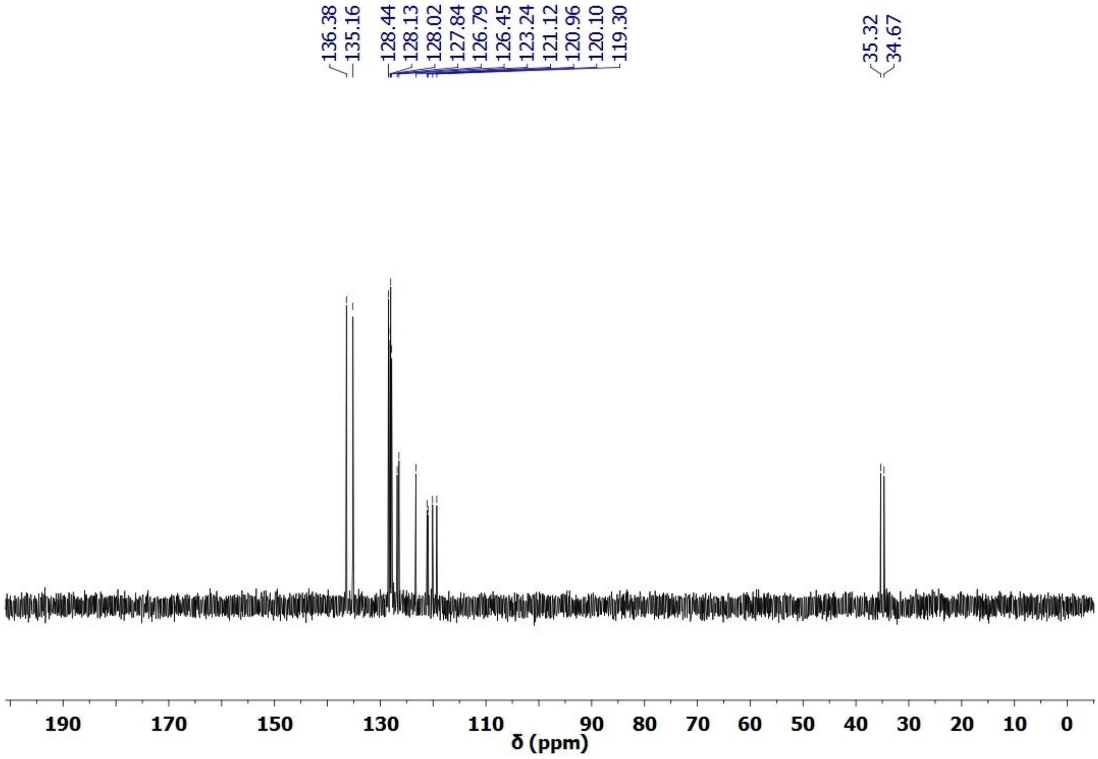

Supplementary Figure 41 | ^{13}C NMR (DEPT135) spectrum (C_6D_6) of **8**.

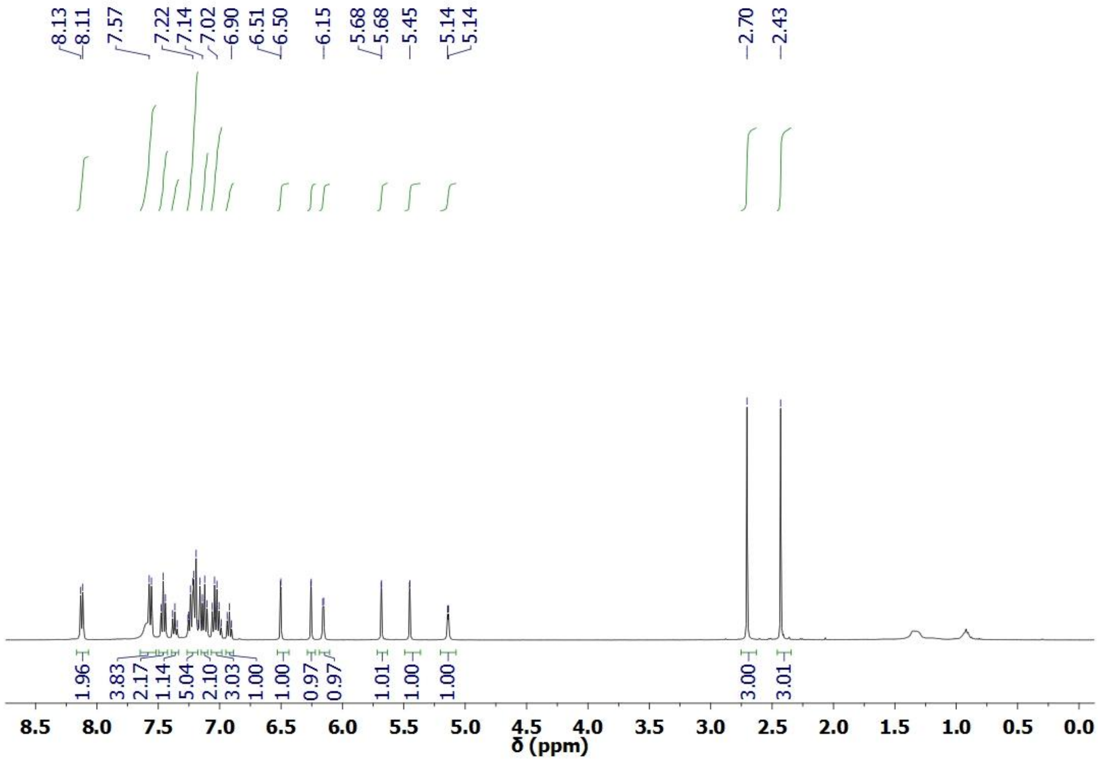

Supplementary Figure 42 | ^{1}H NMR spectrum (C_6D_6) of **9**.

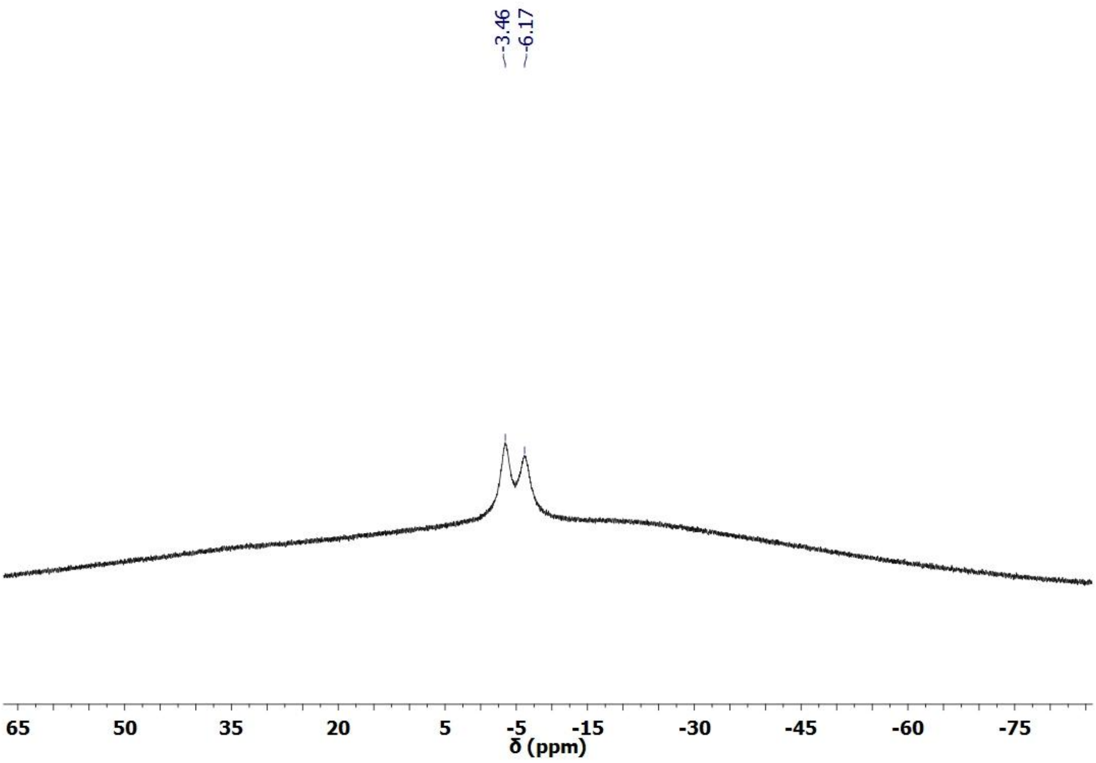

Supplementary Figure 43 | $^{11}B\{^{1}H\}$ NMR spectrum (C_6D_6) of **9**.

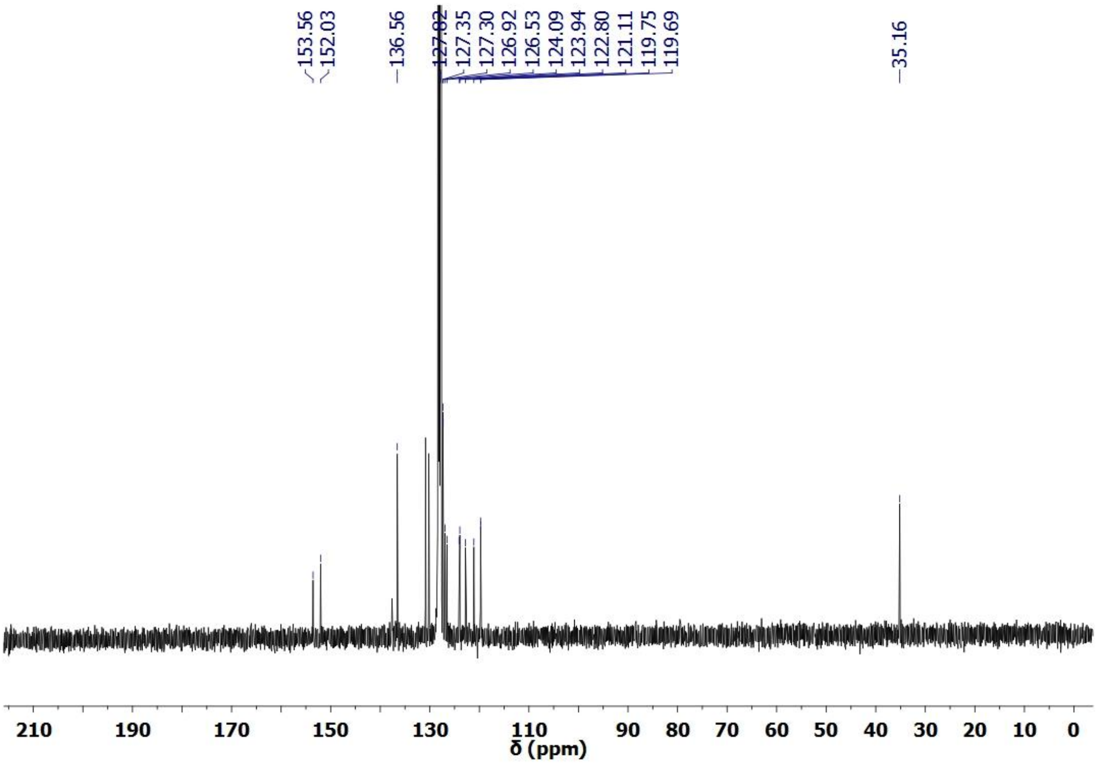

Supplementary Figure 44 | ^{13}C NMR spectrum (C_6D_6) of **9**.

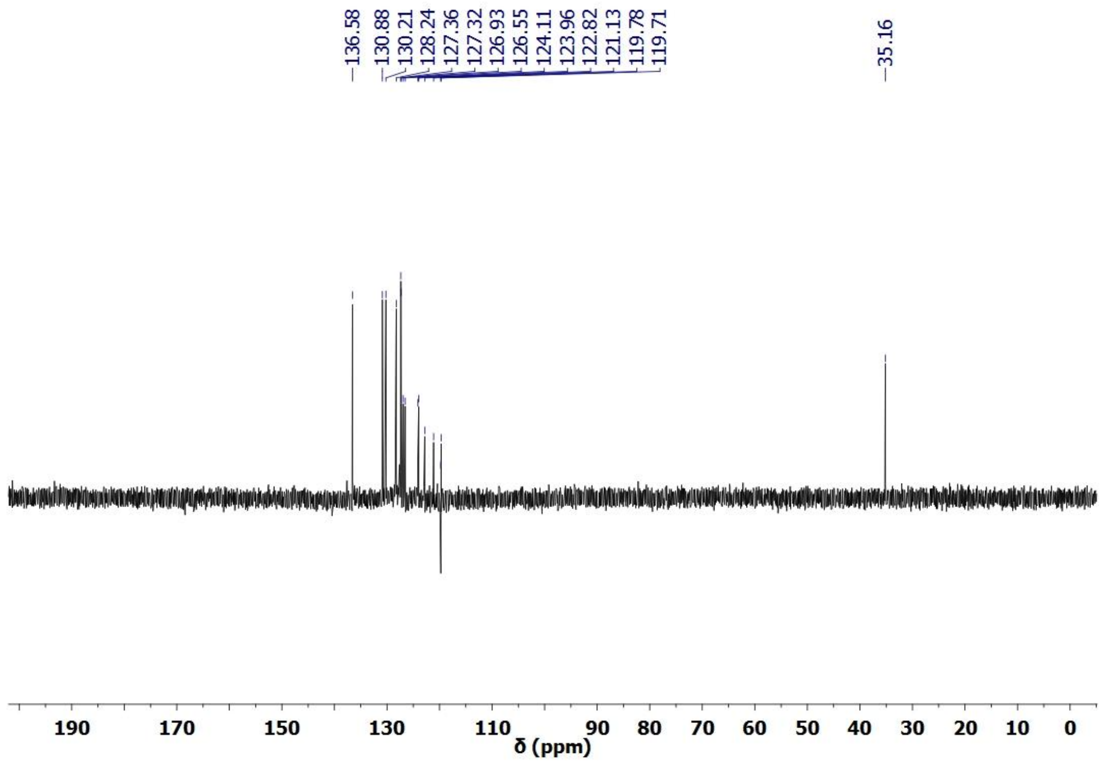

Supplementary Figure 45 | ^{13}C NMR (DEPT135) spectrum (C_6D_6) of **9**.

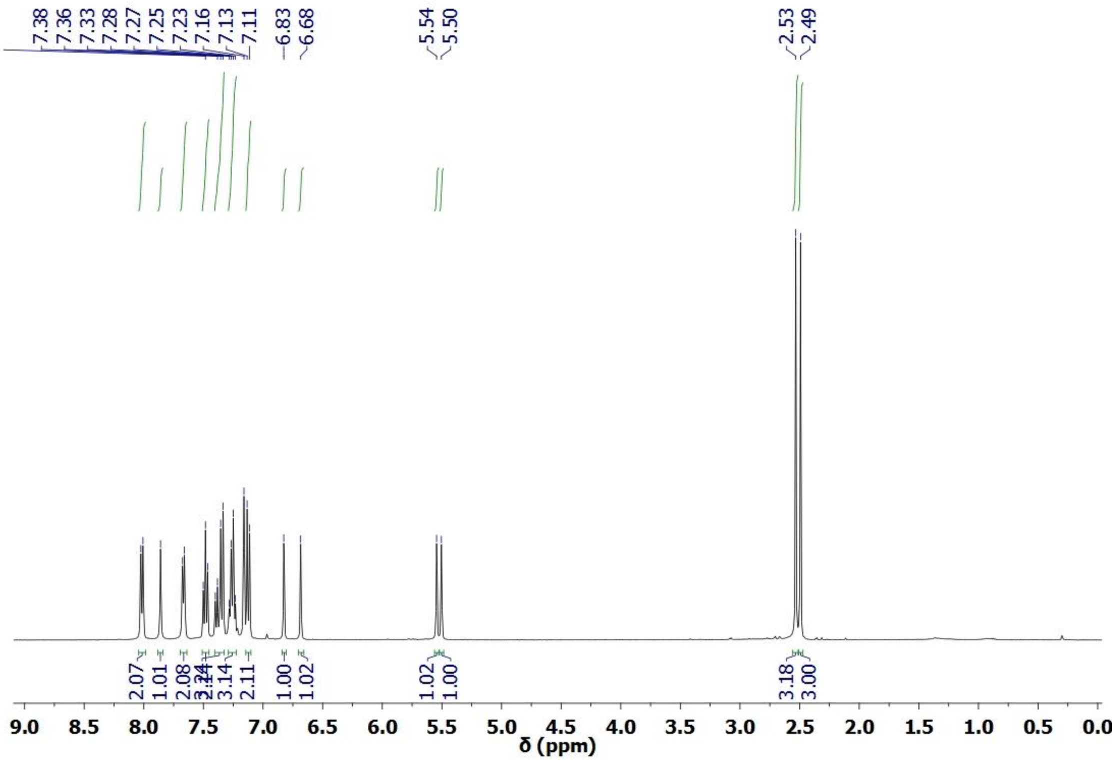

Supplementary Figure 46 | ^{1}H NMR spectrum (C_6D_6) of **10**.

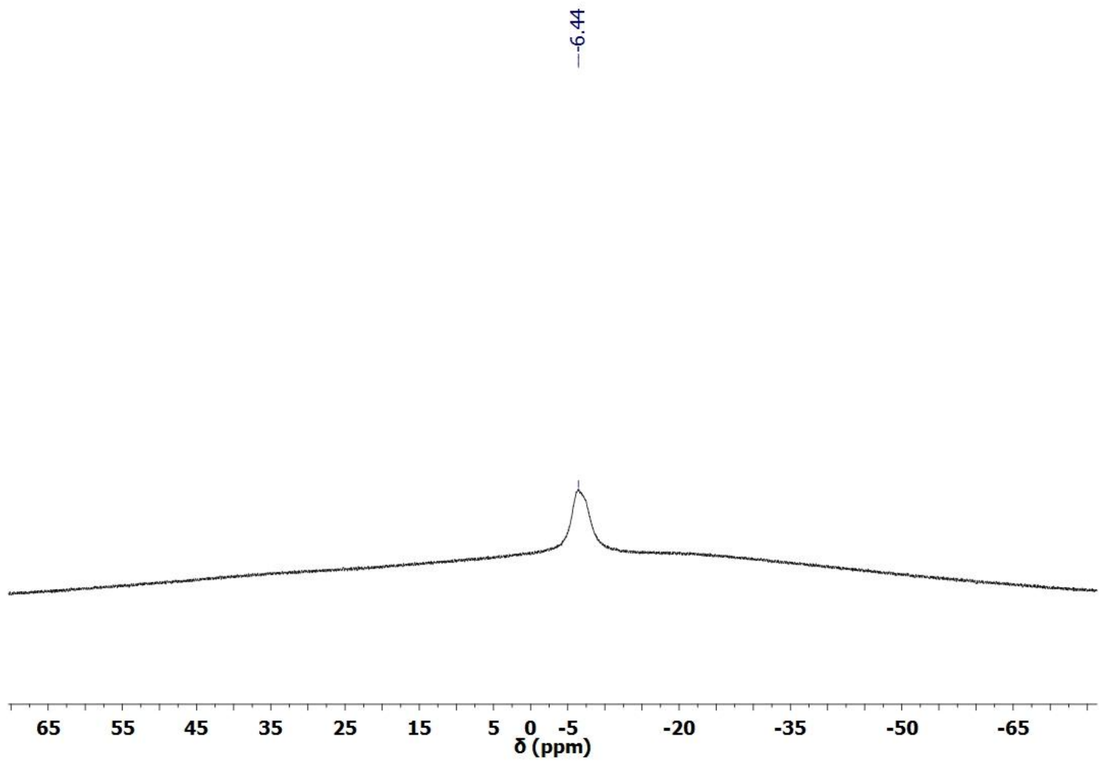

Supplementary Figure 47 | $^{11}B\{^{1}H\}$ NMR spectrum (C_6D_6) of **10**.

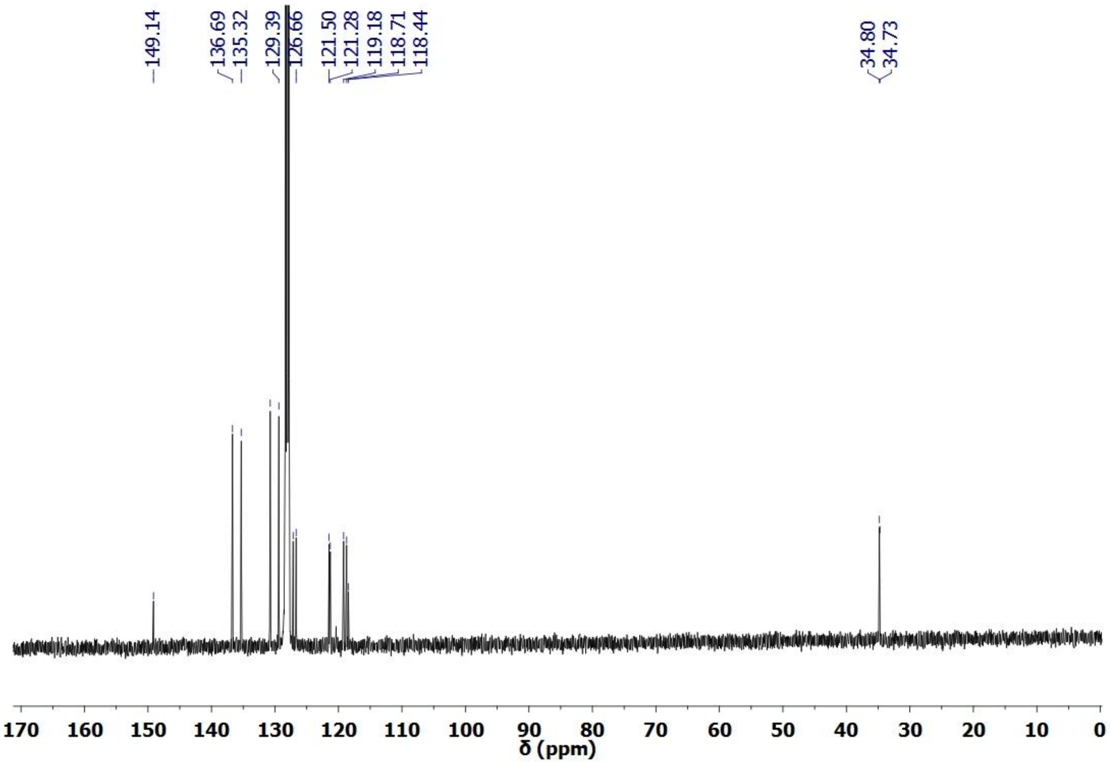

Supplementary Figure 48 | ^{13}C NMR spectrum (C_6D_6) of **10**.

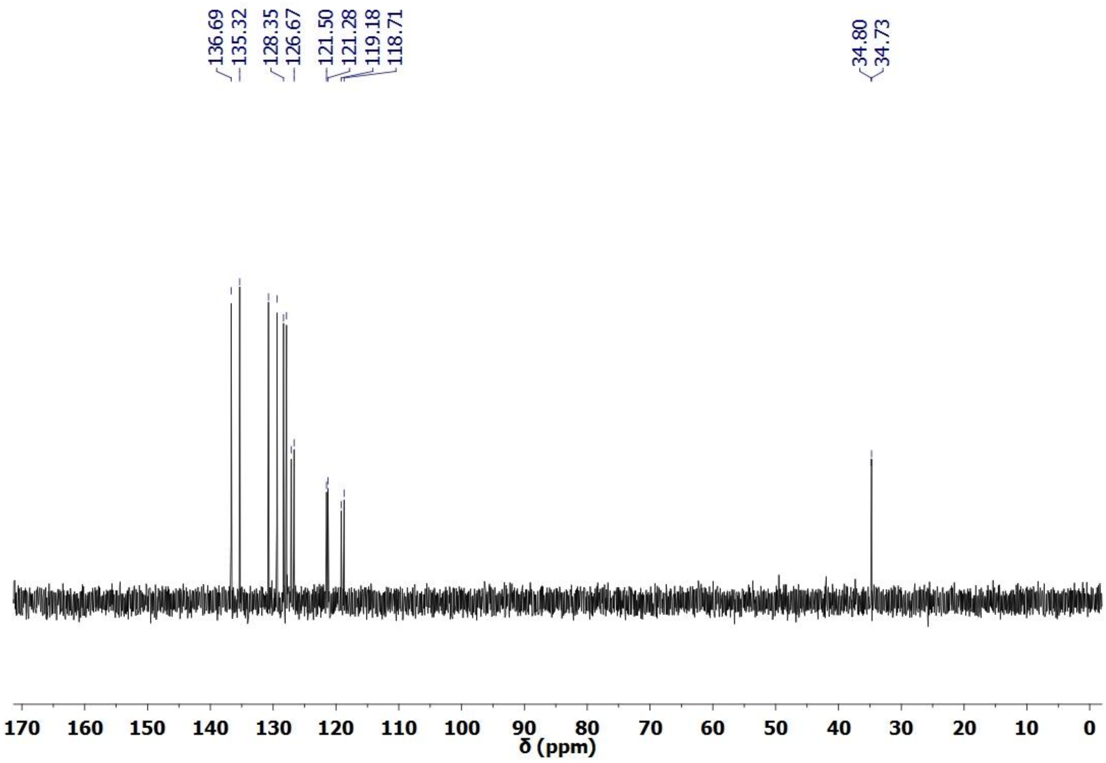

Supplementary Figure 49 | ^{13}C NMR (DEPT135) spectrum (C_6D_6) of **10**.

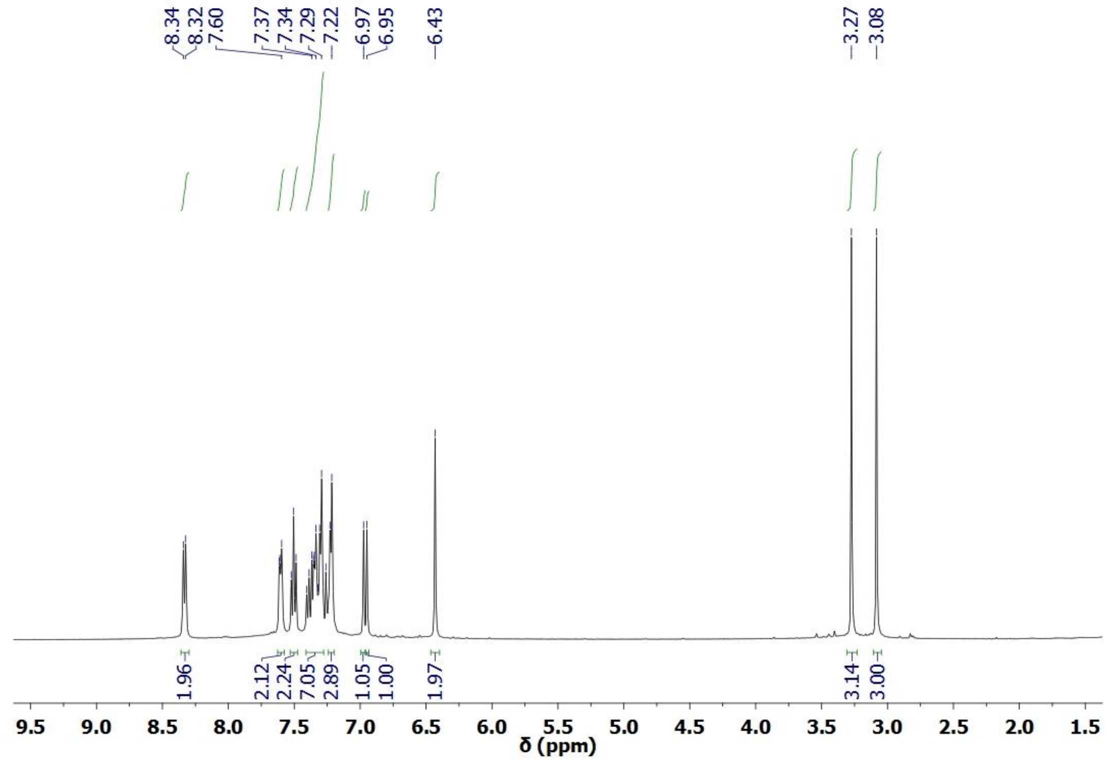

Supplementary Figure 50 | ^{1}H NMR spectrum ($CDCl_3$) of **11a**.

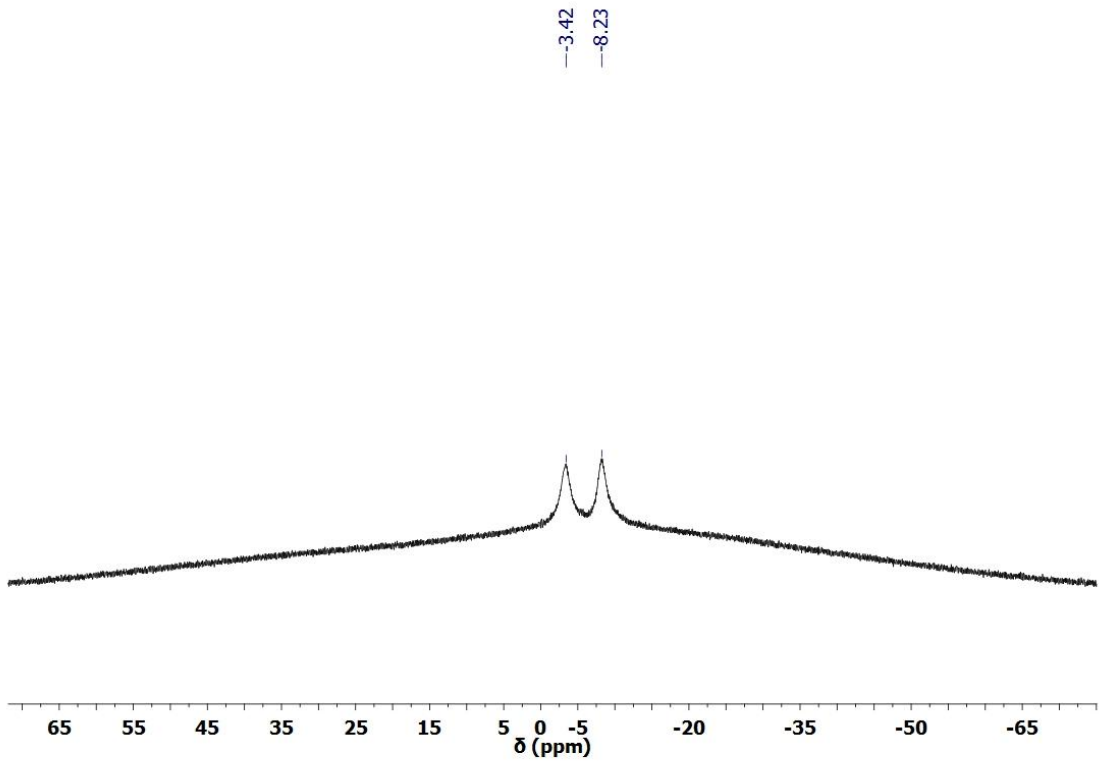

Supplementary Figure 51 | $^{11}B\{^{1}H\}$ NMR spectrum ($CDCl_3$) of **11a**.

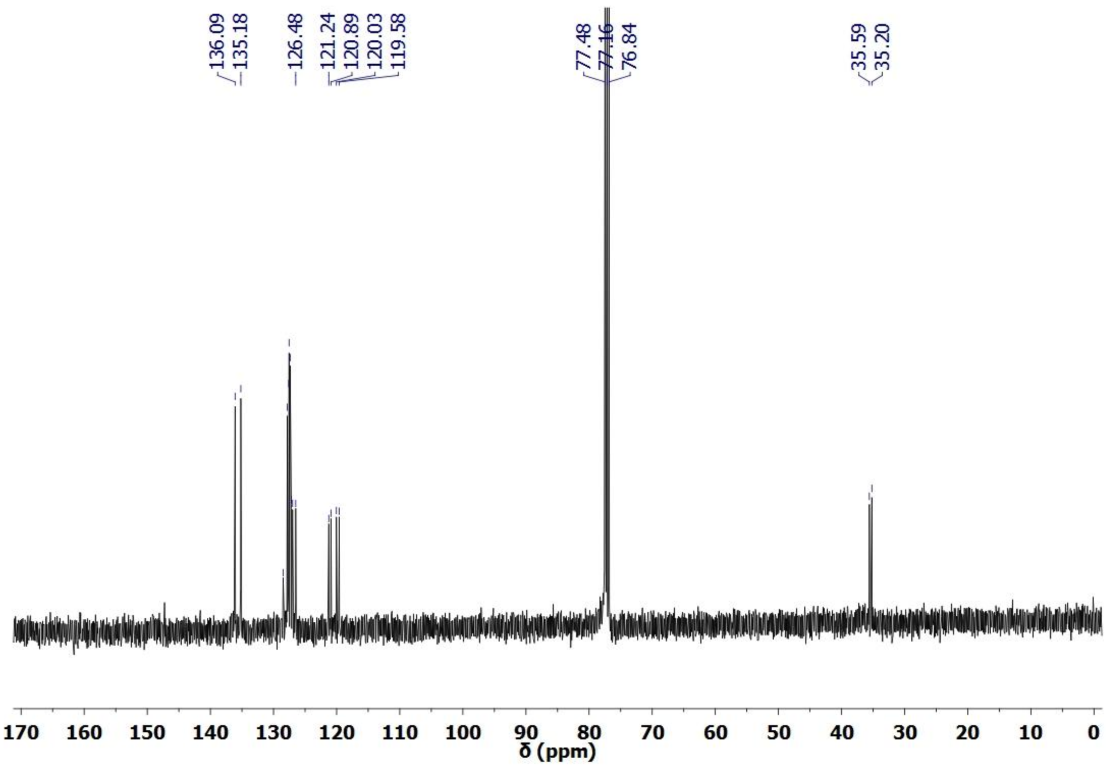

Supplementary Figure 52 | ^{13}C NMR spectrum ($CDCl_3$) of **11a**.

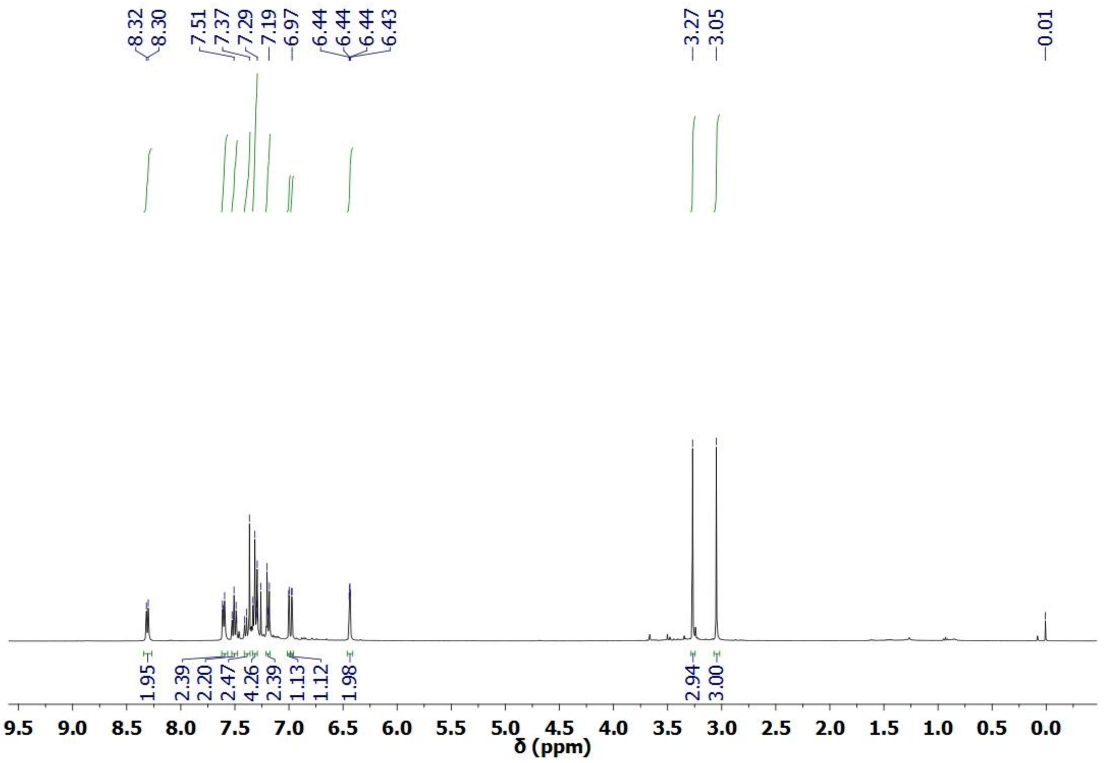

Supplementary Figure 53 | ^{1}H NMR spectrum ($CDCl_3$) of **11b**.

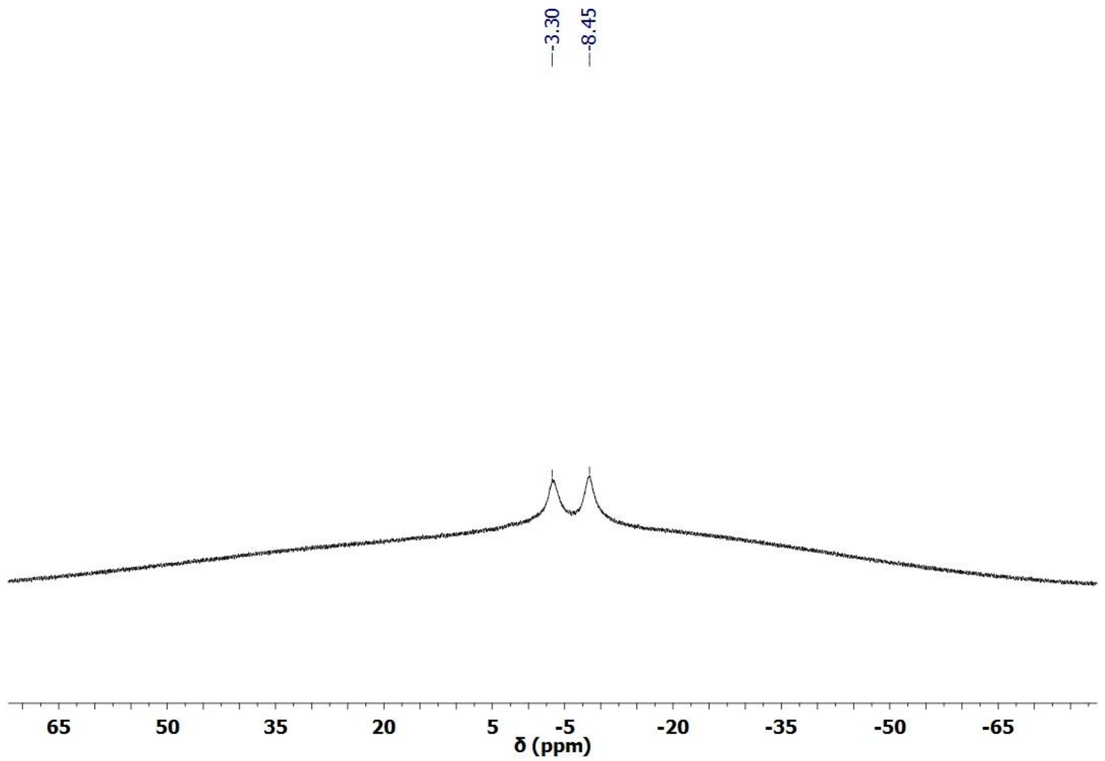

Supplementary Figure 54 | $^{11}B\{^{1}H\}$ NMR spectrum ($CDCl_3$) of **11b**.

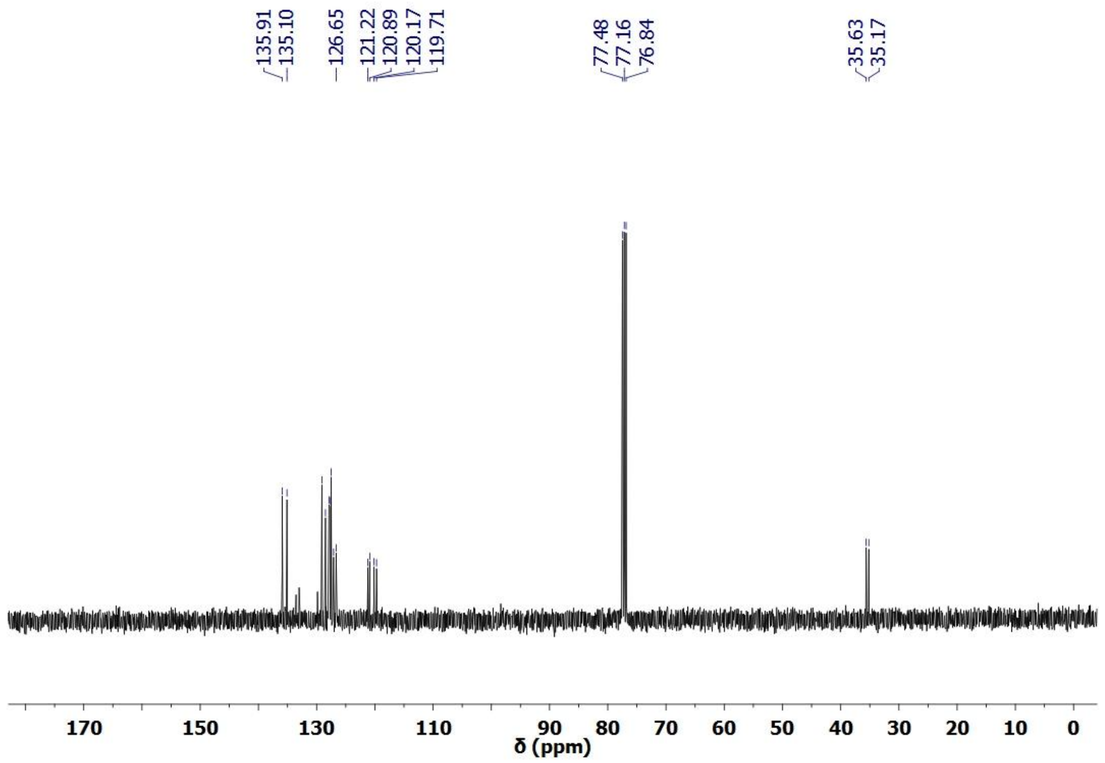

Supplementary Figure 55 | ^{13}C NMR spectrum ($CDCl_3$) of **11b**.

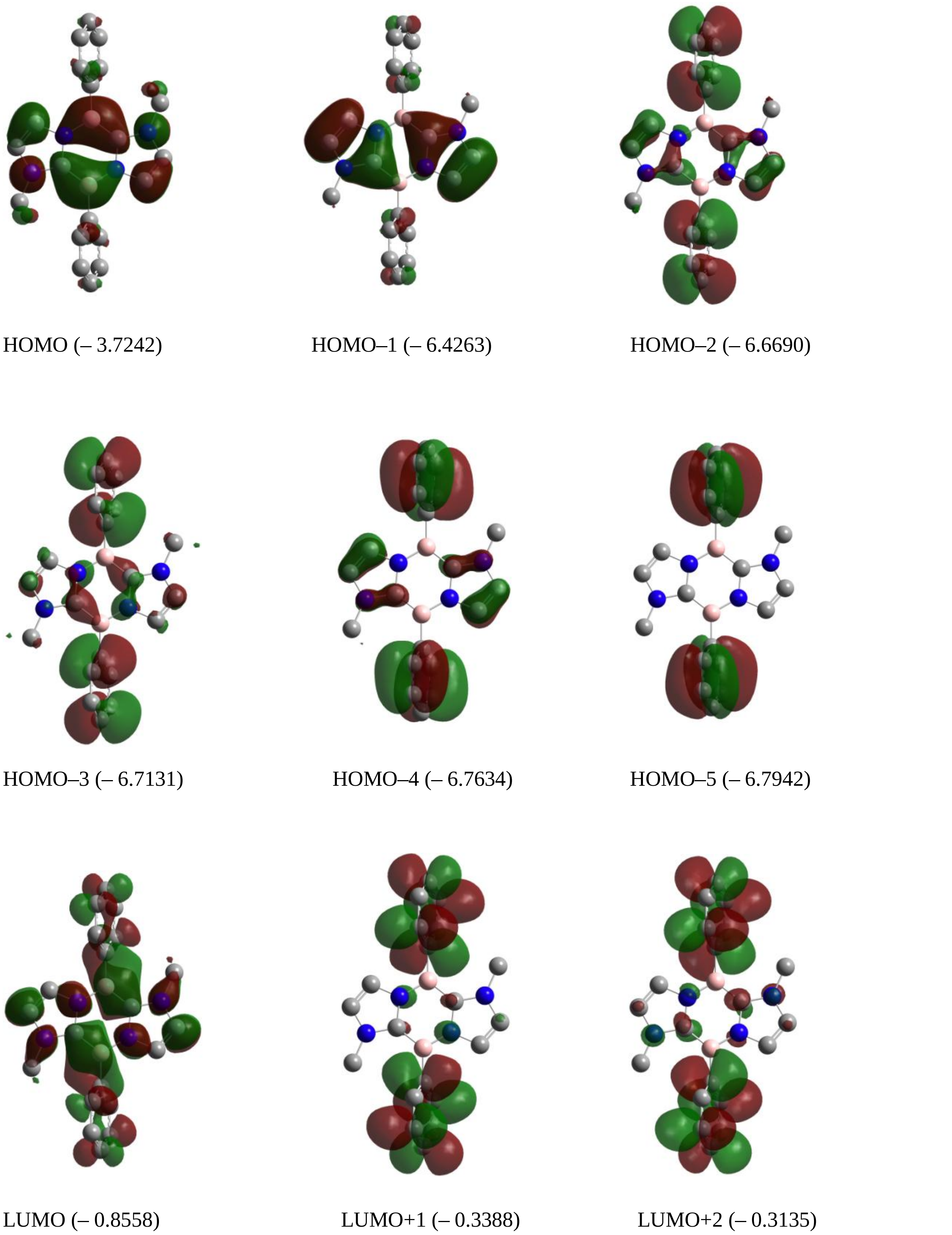

Supplementary Figure 56 | Plots of the frontier orbitals of **2** (Hydrogen atoms are omitted for clarity). The eigenvalues (eV) are provided in the parentheses**.**

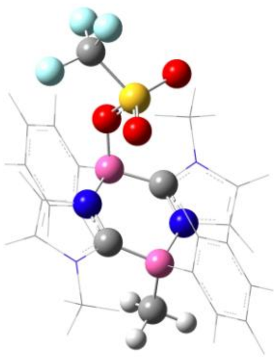

3A (0.0 kcal·mol^{-1})

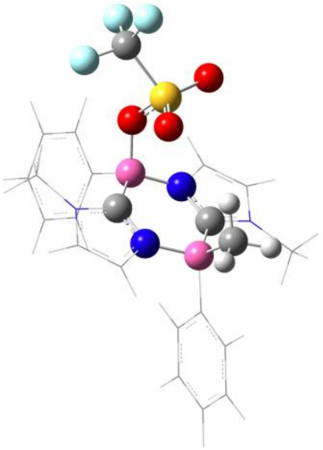

3B (0.2 kcal·mol^{-1})

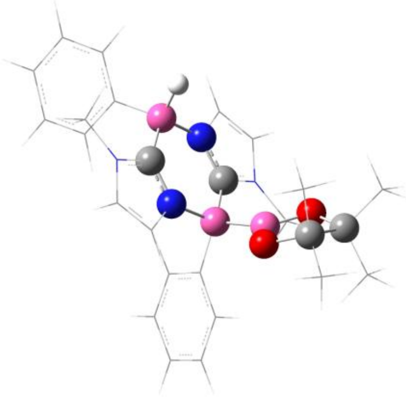

4 (0.0 kcal·mol^{-1})

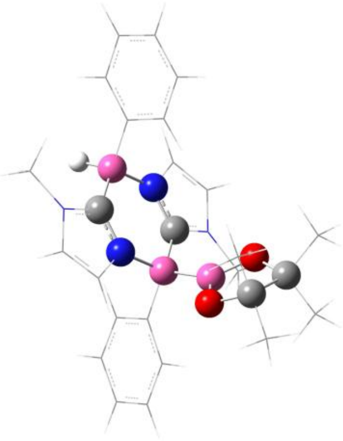

4* (0.4 kcal·mol^{-1})

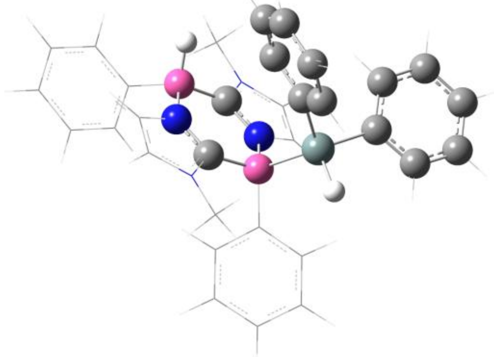

5a (0.0 kcal·mol^{-1})

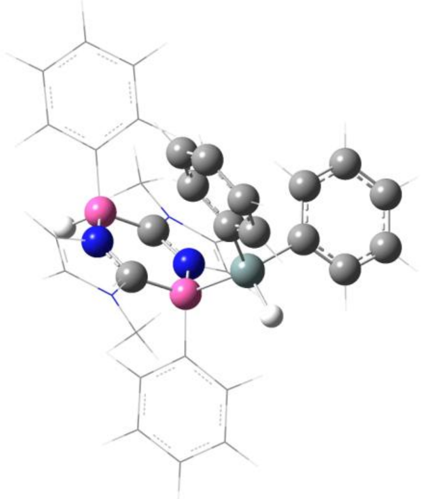

5a* (1.8 kcal·mol^{-1})

Supplementary Figure 57 | Structures and relative energies of **3A**, **3B**, **4**, **4***, **5** and **5*.**

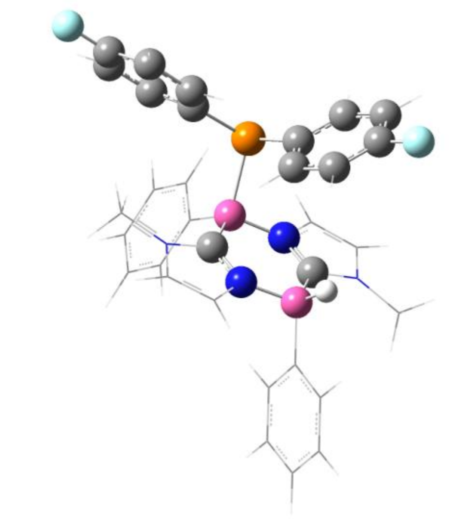

6 (0.0 kcal·mol^{-1})

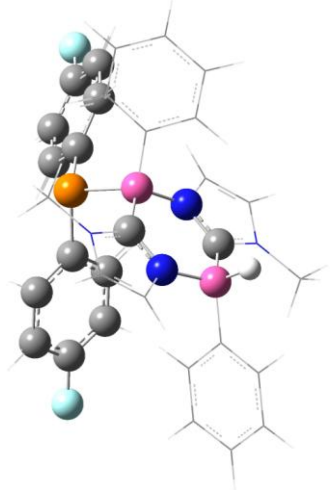

6* (–0.9 kcal·mol^{-1})

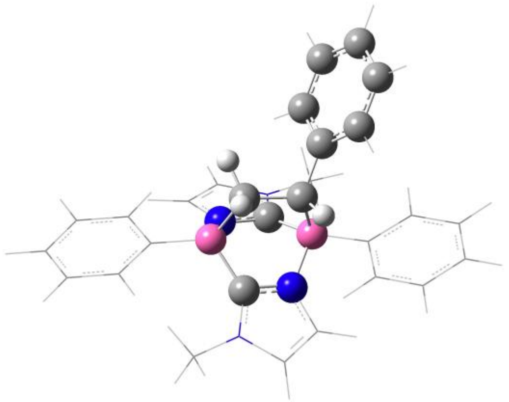

8 (0.0 kcal·mol^{-1})

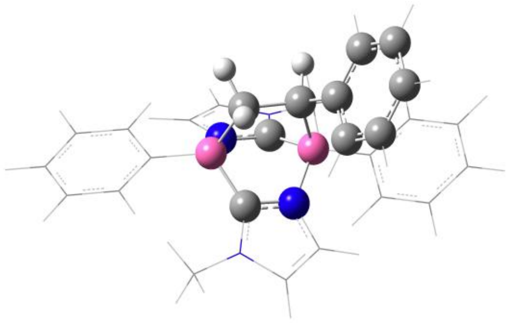

8* (0.6 kcal·mol^{-1})

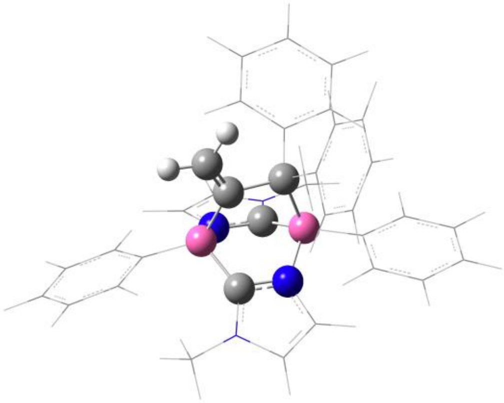

9 (0.0 kcal·mol^{-1})

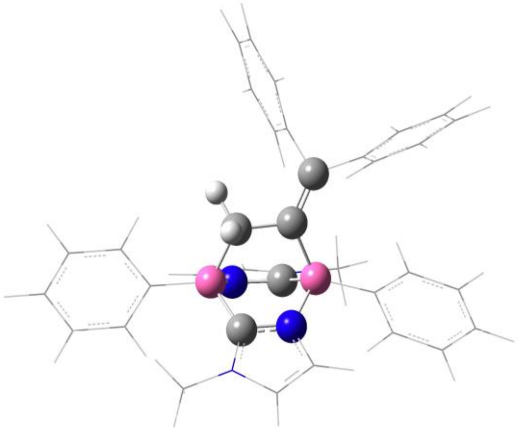

9* (–7.9 kcal·mol^{-1})

Supplementary Figure 58 | Structures and relative energies of **6**, **6***, **8**, **8***, **9** and **9***.

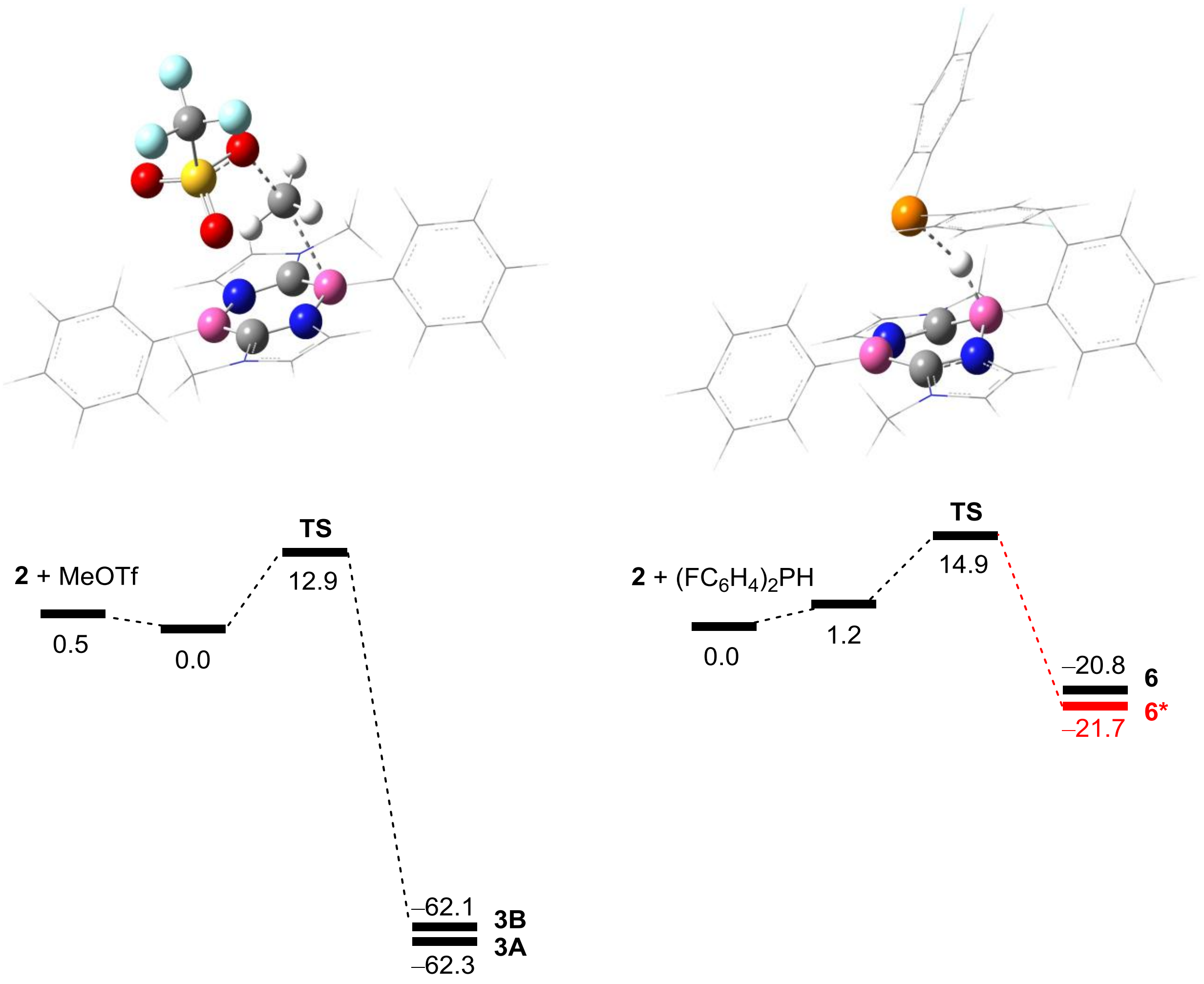

Supplementary Figure 59 | DFT-calculated free energy profiles of concerted mechanism for the formation of **3B** and **6*** from **2** with relative Gibbs free energies in kcal·mol^{-1}.

Supplementary Tables

Supplementary Table 1. Crystal Data and Structure Refinement of **1-4**.

Compounds	**1**	**2**	**3**	**4**
Formula	$C_{20}H_{20}B_2Cl_2N_4$	$C_{20}H_{20}B_2N_4$	$C_{22}H_{23}B_2F_3N_4O_3S$	$C_{26}H_{33}B_3N_4O_2$
Fw	408.92	338.02	502.12	465.99
Cryst syst	triclinic	monoclinic	monoclinic	monoclinic
Space group	P -1	C 1 2/c 1	P 1 21/c 1	C 1 2/c 1
Size (mm^3)	0.150 x 0.180 x 0.300	0.160 x 0.180 x 0.240	0.040 x 0.120 x 0.220	0.080 x 0.100 x 0.420
T, K	103(2)	103(2)	153(2)	103(2)
a, Å	6.7243(2)	28.875(4)	7.2251(7)	29.987(3)
b, Å	8.4506(4)	8.4730(15)	14.5333(12)	7.0147(7)
c, Å	9.7798(4)	19.549(3)	22.5468(19)	25.999(3)
α, deg	76.647(3)	90	90	90
β, deg	85.978(2)	132.202(4)	91.921(5)	110.164(4)
γ, deg	66.7788(19)	90	90	90
V, $Å^3$	496.77(3)	3543.0(10)	2366.2(4)	5133.7(9)
Z	1	8	4	8
d_{calcd} $g·cm^{-3}$	1.367	1.267	1.410	1.206
μ, mm^{-1}	0.340	0.075	0.193	0.075
Refl collected	14248	17195	23885	23680
T_{max}/T_{min}	0.9510/0.9050	0.988/0.9820	0.9920/0.9590	0.9940/0.9690
N_{measd}	3279	4769	4193	4547
[R_{int}]	0.0641	0.0649	0.1319	0.0959
R [I>2sigma(I)]	0.0475	0.0538	0.0757	0.0544
R_w[I>2sigma(I)]	0.1146	0.1094	0.1838	0.1129
GOF	1.025	1.015	1.016	1.019
Largest diff. peak/hole[$e·Å^{-3}$]	0.391/-0.367	0.340/-0.264	0.305/-0.501	0.271/-0.310

Supplementary Table 2. Crystal Data and Structure Refinement of **5a**, **6(C_6H_6)**, **7**, **8**.

Compounds	**5a**	**6**•$(C_6H_6)_1$	**7**	**8**
Formula	$C_{32}H_{32}B_2N_4Si$	$C_{35}H_{32}B_2F_2N_4P$	$C_{21}H_{20}B_2N_4O_2$	$C_{28}H_{28}B_2N_4$
Fw	522.32	599.23	382.03	442.16
Cryst syst	monoclinic	triclinic	orthorhombic	monoclinic
Space group	P 1 21/c 1	P -1	P n a 21	P 1 21/n 1
Size (mm^3)	0.020 x 0.180 x 0.340	0.280 x 0.320 x 0.400	0.060 x 0.220 x 0.230	0.100 x 0.220 x 0.400
T, K	103(2)	103(2)	103(2)	103(2)
a, Å	20.534(8)	8.1150(8)	15.071(5)	8.2613(14)
b, Å	18.455(7)	13.1620(13)	8.378(3)	23.616(4)
c, Å	7.417(3)	16.2320(16)	14.966(5)	12.724(3)
α, deg	90	106.603(3)	90	90
β, deg	94.979(9)	102.639(3)	90	106.778(5)
γ, deg	90	104.799(3)	90	90
V, $Å^3$	2800.1(19)	1524.6(3)	1889.7(11)	2376.8(8)
Z	4	2	4	4
d_{calcd} $g \cdot cm^{-3}$	1.239	1.305	1.343	1.236
μ, mm^{-1}	0.113	0.135	0.087	0.073
Refl collected	34178	43901	13022	20107
T_{max}/T_{min}	0.9980/0.9630	0.9630/0.9480	0.9950/0.9800	0.9930/0.9720
N_{measd}	5355	10572	3319	4672
[R_{int}]	0.2111	0.1015	0.1858	0.1015
R [I>2sigma(I)]	0.0752	0.0635	0.0847	0.0630
R_w[I>2sigma(I)]	0.1387	0.1222	0.1460	0.1307
GOF	0.989	1.037	1.013	1.008
Largest diff. peak/hole[$e \cdot Å^{-3}$]	0.275/-0.367	0.416/-0.391	0.255/-0.273	0.242/-0.262

Supplementary Table 3. Crystal Data and Structure Refinement of **9**, **10(C_7H_8)$_{0.5}$**, **11b(THF)**.

Compounds	**9**	**10**•$(C_7H_8)_{0.5}$	**11b**•$(THF)_1$
Formula	$C_{35}H_{32}B_2N_4$	$C_{31.50}H_{29}B_2BrN_4$	$C_{31}H_{32}B_2ClN_5O$
Fw	530.26	565.12	547.68
Cryst syst	monoclinic	monoclinic	monoclinic
Space group	P 1 21 1	P 1 21/n 1	P 1 21/c 1
Size (mm^3)	0.300 x 0.320 x 0.420	0.100 x 0.160 x 0.200	0.060 x 0.120 x 0.240
T, K	103(2)	103(2)	103(2)
a, Å	9.1900(10)	7.4966(7)	7.5717(11)
b, Å	30.098(3)	14.1674(14)	13.808(2)
c, Å	10.4179(11)	26.033(2)	27.350(4)
α, deg	90	90	90
β, deg	102.688(3)	95.734(3)	96.647(5)
γ, deg	90	90	90
V, $Å^3$	2811.2(5)	2751.1(5)	2840.2(8)
Z	4	4	4
d_{calcd} $g{\cdot}cm^{-3}$	1.253	1.364	1.281
μ, mm^{-1}	0.073	1.523	0.169
Refl collected	26341	39348	14028
T_{max}/T_{min}	0.9780/0.9700	0.8630/0.7500	0.9900/0.9610
N_{measd}	14158	6552	5258
[R_{int}]	0.0598	0.1068	0.0795
R [I>2sigma(I)]	0.0665	0.0546	0.0695
R_w[I>2sigma(I)]	0.1485	0.0985	0.1359
GOF	1.009	1.064	1.024
Largest diff. peak/hole[$e{\cdot}Å^{-3}$]	0.389/-0.284	0.567/-0.537	0.290/-0.435

Supplementary Table 4. Relative resonance stabilization energy calculation

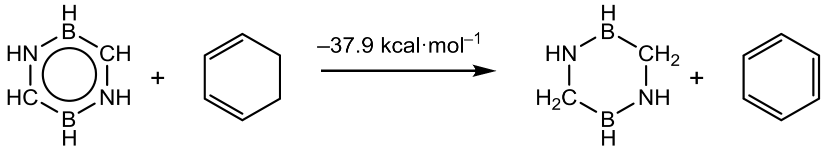

	E (au)	ZPE (au)	E+ZPE (au)
**2''**	-239.163239	0.095022	-239.068217
**C_6H_8**	-233.483882	0.121809	-233.362073
**2" + 2H**	-240.396242	0.116549	-240.279693
**C_6H_6**	-232.311245	0.100159	-232.211086
RSE (kcal/mol)	-37.9		-38.0

Supplementary Table 5. The NPA charges of **2**.

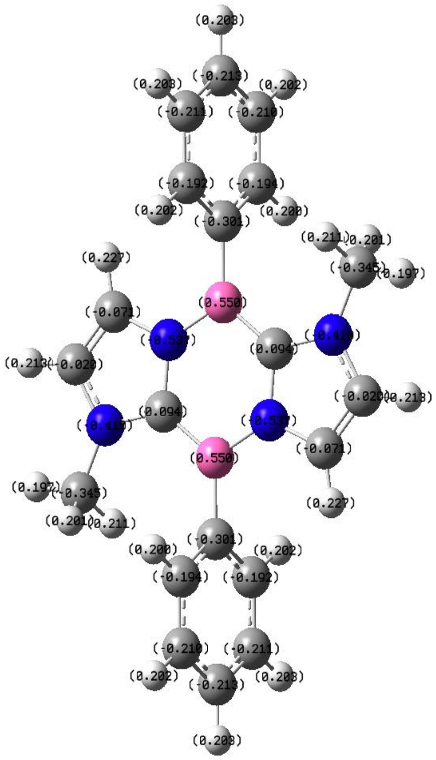

Atom	No	Natural Charge	Core	Valence	Rydberg	Total
B	1	0.55035	1.99880	2.43350	0.01735	4.44965
C	2	0.09396	1.99887	3.88105	0.02612	5.90604
C	3	-0.07061	1.99914	4.05032	0.02115	6.07061
H	4	0.22688	0.00000	0.77166	0.00146	0.77312

C	5	-0.01953	1.99914	4.00072	0.01967	6.01953
H	6	0.21273	0.00000	0.78584	0.00143	0.78727
C	7	-0.34466	1.99934	4.33291	0.01242	6.34466
H	8	0.19671	0.00000	0.80211	0.00118	0.80329
H	9	0.21089	0.00000	0.78713	0.00197	0.78911
H	10	0.20113	0.00000	0.79670	0.00218	0.79887
C	11	-0.30129	1.99900	4.28241	0.01988	6.30129
C	12	-0.19402	1.99908	4.17721	0.01773	6.19402
H	13	0.20040	0.00000	0.79790	0.00170	0.79960
C	14	-0.20986	1.99914	4.19196	0.01876	6.20986
H	15	0.20235	0.00000	0.79605	0.00160	0.79765
C	16	-0.21277	1.99915	4.19496	0.01866	6.21277
H	17	0.20296	0.00000	0.79559	0.00145	0.79704
C	18	-0.21061	1.99915	4.19258	0.01888	6.21061
H	19	0.20262	0.00000	0.79576	0.00162	0.79738
C	20	-0.19242	1.99908	4.17615	0.01718	6.19242
H	21	0.20150	0.00000	0.79695	0.00156	0.79850
N	22	-0.53693	1.99923	5.52196	0.01573	7.53693
N	23	-0.40978	1.99925	5.39554	0.01498	7.40978
C	24	0.09396	1.99887	3.88105	0.02612	5.90604
B	25	0.55035	1.99880	2.43350	0.01735	4.44965
N	26	-0.53693	1.99923	5.52196	0.01573	7.53693
N	27	-0.40978	1.99925	5.39554	0.01498	7.40978
C	28	-0.30129	1.99900	4.28241	0.01988	6.30129
C	29	-0.07061	1.99914	4.05032	0.02115	6.07061
C	30	-0.01953	1.99914	4.00072	0.01967	6.01953
C	31	-0.34466	1.99934	4.33291	0.01242	6.34466
C	32	-0.19401	1.99908	4.17720	0.01773	6.19401
C	33	-0.19242	1.99908	4.17615	0.01718	6.19242
H	34	0.22688	0.00000	0.77166	0.00146	0.77312
H	35	0.21273	0.00000	0.78584	0.00143	0.78727
H	36	0.19671	0.00000	0.80211	0.00118	0.80329
H	37	0.21089	0.00000	0.78713	0.00197	0.78911
H	38	0.20113	0.00000	0.79670	0.00218	0.79887
H	39	0.20040	0.00000	0.79790	0.00170	0.79960
C	40	-0.20986	1.99914	4.19196	0.01876	6.20986
C	41	-0.21061	1.99915	4.19258	0.01888	6.21061
H	42	0.20150	0.00000	0.79695	0.00156	0.79850
H	43	0.20235	0.00000	0.79605	0.00160	0.79765
C	44	-0.21277	1.99915	4.19496	0.01866	6.21277
H	45	0.20262	0.00000	0.79575	0.00162	0.79738
H	46	0.20296	0.00000	0.79559	0.00145	0.79704
* Total *		0.00000	51.97675	125.51388	0.50937	178.00000

Supplementary Table 6. Raw energy data.

(a) Formation of **3B**

	E [au]	ZPE [au]	Gcorr [au]	Edisp [au]	E(SCRF) [au]	ΔG (w/o Edisp) [kcal/mol]	ΔG (w/ Edisp) [kcal/mol]
**2**	-1043.229025	0.370255	0.316126	-0.108983	-1043.232870		
MeOTf	-1001.528510	0.066268	0.030242	-0.018546	-1001.531984		
**2** + MeOTf	-2044.757535	0.436523	0.346368	-0.127529	-2044.764854	0.0	0.5
RC	-2044.763433	0.437438	0.363227	-0.141308	-2044.768754	8.1	0.0
TS-**3B**	-2044.736154	0.435668	0.365274	-0.146309	-2044.745220	24.2	12.9
**3B**	-2044.859583	0.438709	0.372712	-0.152146	-2044.866313	-47.1	-62.1
**3A**	-2044.860686	0.438729	0.373132	-0.152292	-2044.867016	-47.3	-62.3

(b) Formation of **4**

	E [au]	ZPE [au]	Gcorr [au]	Edisp [au]	E(SCRF) [au]	ΔG (w/o Edisp) [kcal/mol]	ΔG (w/ Edisp) [kcal/mol]
**2**	-1043.229025	0.370255	0.316126	-0.108983	-1043.232870		
HBpin	-411.987602	0.189404	0.156227	-0.036377	-411.989922		
**2** + HBpin	-1455.216627	0.559659	0.472353	-0.145360	-1455.222792	0.0	0.0
RC	-1455.218219	0.560385	0.488846	-0.154543	-1455.223679	9.8	4.0
TS-**4**	-1455.181360	0.559012	0.495583	-0.169056	-1455.187213	36.9	22.0
**4**	-1455.241014	0.561751	0.494456	-0.164307	-1455.246579	-1.1	-12.9
**4***	-1455.241411	0.562143	0.496233	-0.164924	-1455.247033	-0.2	-12.5

(c) Formation of **5a**

	E [au]	ZPE [au]	Gcorr [au]	Edisp [au]	E(SCRF) [au]	ΔG (w/o Edisp) [kcal/mol]	ΔG (w/ Edisp) [kcal/mol]
**2**	-1043.229025	0.370255	0.316126	-0.108983	-1043.232870		
Ph_2SiH_2	-754.146726	0.197951	0.157549	-0.053425	-754.148637		
**2** + substrate	-1797.375751	0.568206	0.473675	-0.162408	-1797.381507	0.0	0.0
RC	-1797.377001	0.568738	0.489124	-0.173167	-1797.382180	9.3	2.5
TS-**5a**	-1797.340509	0.567725	0.497180	-0.194173	-1797.346572	36.7	16.7
**5a**	-1797.398588	0.572108	0.501486	-0.194300	-1797.404261	3.2	-16.8
**5a***	-1797.396976	0.572135	0.501889	-0.193273	-1797.402684	4.4	-15.0

(d) Formation of **6***

	E [au]	ZPE [au]	Gcorr [au]	Edisp [au]	E(SCRF) [au]	ΔG (w/o Edisp) [kcal/mol]	ΔG (w/ Edisp) [kcal/mol]
**2**	-1043.229025	0.370255	0.316126	-0.108983	-1043.232870		
$(FC_6H_4)_2PH$	-1003.926755	0.173859	0.131822	-0.054273	-1003.929661		
**2** + $(FC_6H_4)_2PH$	-2047.155780	0.544114	0.447948	-0.163256	-2047.162531	0.0	0.0
RC	-2047.158100	0.544862	0.464108	-0.175946	-2047.164115	9.1	1.2
TS-**6***	-2047.123454	0.542733	0.470198	-0.193611	-2047.130628	34.0	14.9
**6***	-2047.181374	0.547777	0.476204	-0.200318	-2047.188327	1.5	-21.7
**6**	-2047.183005	0.548143	0.478012	-0.199325	-2047.189700	1.8	-20.8

(e) Formation of **8** and **8***

	E [au]	ZPE [au]	Gcorr [au]	Edisp [au]	E(SCRF) [au]	ΔG (w/o Edisp) [kcal/mol]	ΔG (w/ Edisp) [kcal/mol]
**2**	-1043.229025	0.370255	0.316126	-0.108983	-1043.232870		
styrene	-309.730763	0.132766	0.101219	-0.027609	-309.732306		
**2** + styrene	-1352.959788	0.503021	0.417345	-0.136592	-1352.965176	0.0	0.0
RC	-1352.961548	0.503482	0.431967	-0.146593	-1352.966325	8.5	2.2
TS-**8**	-1352.940829	0.504400	0.442929	-0.167506	-1352.945744	28.2	8.8
TS-**8***	-1352.940651	0.504497	0.443736	-0.165048	-1352.946034	28.6	10.7
**8**	-1352.993330	0.507888	0.447886	-0.165025	-1352.998306	-1.6	-19.5
**8***	-1352.991823	0.507796	0.447185	-0.164672	-1352.996984	-1.2	-18.9

(f) Formation of **9** and **9***

	E [au]	ZPE [au]	Gcorr [au]	Edisp [au]	E(SCRF) [au]	ΔG (w/o Edisp) [kcal/mol]	ΔG (w/ Edisp) [kcal/mol]
**2**	-1043.229025	0.370255	0.316126	-0.108983	-1043.232870		
H_2CCCPh_2	-578.916410	0.217713	0.177842	-0.058059	-578.918521		
**2** + H_2CCCPh_2	-1622.145435	0.587968	0.493968	-0.167043	-1622.151391	0.0	0.0
RC	-1622.146926	0.588549	0.509678	-0.177508	-1622.152256	9.3	2.7
TS-**9**	-1622.108712	0.590454	0.524008	-0.210056	-1622.114327	42.1	15.1
TS-**9***	-1622.110573	0.589074	0.518893	-0.201598	-1622.116154	37.8	16.1
**9**	-1622.163174	0.593918	0.528919	-0.217056	-1622.168582	11.1	-20.2
**9***	-1622.186882	0.593054	0.525636	-0.202141	-1622.192790	-6.1	-28.1

Supplementary Table 7. Calculated geometries (atom, x-, y-, z-positions in Å) utilized for mechanism study.

=== **2** ===

N	-2.296552	-0.800973	-2.217042
C	-1.167135	-0.634504	-2.979503
C	-0.226824	-0.006209	-2.233746
N	-0.744345	1.456581	2.651785
C	-1.876517	1.297230	3.411693
C	-2.827611	0.697898	2.655874
C	-4.451253	-0.656601	0.410739
C	1.381342	1.389798	-0.000089
C	1.679555	2.753995	-0.166660
C	2.991270	3.207668	-0.310891
C	4.049715	2.300966	-0.298019
C	3.783834	0.940630	-0.144599
C	2.469274	0.497256	-0.000371
C	-5.530712	0.059712	-0.136564
C	-6.849478	-0.372676	0.008947
C	-7.128070	-1.540798	0.716371
C	-6.078726	-2.266884	1.278836
C	-4.764011	-1.826608	1.127324
C	0.487921	2.070735	3.107960
C	-3.523188	-1.430742	-2.666933
N	-0.767105	0.232312	-0.963490
B	-0.107277	0.875559	0.161209
C	-0.977755	0.956439	1.372381
N	-2.290228	0.467354	1.382978
B	-2.955454	-0.162134	0.253344
C	-2.079311	-0.257648	-0.952528
H	-1.118083	-0.979766	-3.999256
H	0.775432	0.289672	-2.485454
H	-1.917855	1.621573	4.438630
H	-3.832472	0.408810	2.905057
H	0.868065	3.475310	-0.188545
H	3.186509	4.267612	-0.437019
H	5.070728	2.649743	-0.408991
H	4.600323	0.225974	-0.133805
H	2.283795	-0.565774	0.122983
H	-5.335812	0.977627	-0.683225
H	-7.659024	0.204222	-0.426221
H	-8.151804	-1.880243	0.831072
H	-6.284267	-3.177046	1.832781
H	-3.960778	-2.408676	1.569650
H	1.340791	1.425065	2.891748
H	0.422254	2.223249	4.185522
H	0.656865	3.034967	2.620880
H	-3.349632	-1.865744	-3.651451
H	-3.825142	-2.221166	-1.977145
H	-4.339468	-0.706951	-2.735460

Formation of **3B**

=== **MeOTf** ===

C	-0.932551	-3.125756	1.445392
O	-0.264319	-4.340698	0.990227
C	1.288370	-3.969686	-1.170877
F	1.260242	-2.652727	-0.934411
F	1.406492	-4.178429	-2.478268
F	2.325581	-4.504104	-0.537023
S	-0.323865	-4.754902	-0.569446
O	-0.137999	-6.182562	-0.632916
O	-1.407246	-4.060456	-1.235995
H	-1.964472	-3.111958	1.099869
H	-0.888725	-3.191910	2.529743
H	-0.393952	-2.246783	1.092717

=== **RC** ===

N	-2.025675	0.956504	-3.042462
C	-1.017007	1.272382	-3.917853
C	0.069031	1.666913	-3.211181
N	0.416608	1.878981	1.900785
C	-0.588890	1.559478	2.777734
C	-1.677535	1.176114	2.069573
C	-3.712880	0.454153	-0.124093
C	2.116711	2.350507	-1.011602
C	2.404153	3.598187	-1.596838
C	3.712333	4.001942	-1.860911
C	4.780266	3.160717	-1.549008
C	4.525494	1.916095	-0.975894
C	3.213426	1.519978	-0.714840
C	-4.833069	1.255642	-0.409218
C	-6.133105	0.822027	-0.147090
C	-6.350093	-0.433447	0.417756
C	-5.257380	-1.244736	0.723520
C	-3.961403	-0.803657	0.458724

C	1.750493	2.299152	2.292210
C	-3.336935	0.468133	-3.424010
N	-0.252409	1.606722	-1.847085
B	0.629847	1.894488	-0.728211
C	-0.031796	1.682912	0.599624
N	-1.354900	1.242057	0.709922
B	-2.238128	0.945119	-0.412389
C	-1.578892	1.159704	-1.735084
C	0.694964	-1.814852	-1.059070
O	1.524877	-1.762940	0.148085
C	1.702141	-3.976529	1.639480
F	1.334576	-4.694135	0.572664
F	1.278756	-4.585606	2.744635
F	3.027646	-3.880412	1.665359
S	0.917139	-2.259515	1.551628
O	1.537222	-1.472978	2.591612
O	-0.514909	-2.459853	1.453140
H	-1.148687	1.190547	-4.984512
H	1.038710	1.983898	-3.549764
H	-0.450110	1.626705	3.843979
H	-2.649079	0.862632	2.405010
H	1.587738	4.271814	-1.840692
H	3.899270	4.974277	-2.304846
H	5.799209	3.472239	-1.751566
H	5.348606	1.251660	-0.733997
H	3.036747	0.545055	-0.271156
H	-4.684312	2.243107	-0.835928
H	-6.975336	1.466468	-0.377163
H	-7.359137	-0.774323	0.622883
H	-5.414778	-2.222427	1.167106
H	-3.125073	-1.450674	0.706425
H	2.477216	1.497217	2.142889
H	1.731658	2.566734	3.348799
H	2.060168	3.167592	1.710151
H	-3.333453	0.263868	-4.495038
H	-3.575667	-0.451600	-2.886222
H	-4.116391	1.202107	-3.204233
H	0.456731	-2.849966	-1.301068
H	-0.206964	-1.222387	-0.917879
H	1.326011	-1.373668	-1.825978

=== **TS-3B** ===

N	-2.588603	-0.523103	-2.415705
C	-1.547570	-0.201917	-3.266547
C	-0.514266	0.257897	-2.529826
N	-0.418049	0.681411	2.576680
C	-1.443939	0.378538	3.427400
C	-2.510823	-0.024601	2.686278
C	-4.440563	-0.859503	0.415458
C	1.362773	1.161357	-0.263054
C	1.581144	2.517393	-0.565222
C	2.866379	3.017323	-0.773866
C	3.966741	2.165582	-0.688943
C	3.772453	0.816433	-0.396826
C	2.486321	0.318463	-0.186479
C	-5.351062	0.180923	0.685980
C	-6.710896	-0.061374	0.873039
C	-7.205353	-1.363189	0.797835
C	-6.327382	-2.413323	0.539835
C	-4.967212	-2.160872	0.355926
C	0.950694	0.950849	3.009593
C	-3.864196	-1.078094	-2.837608
N	-0.901007	0.223991	-1.181191
B	-0.097209	0.615271	-0.037791
C	-0.827779	0.455722	1.280411
N	-2.135454	0.007622	1.350642
B	-2.907597	-0.516212	0.200421
C	-2.202541	-0.288128	-1.111298
C	-1.485902	-2.614323	0.397755
O	-0.200145	-3.963365	0.919766
C	1.551064	-5.013226	2.581469
F	0.579353	-5.458771	3.385223
F	2.633106	-4.752683	3.325620
F	1.856630	-5.972480	1.703724
S	1.010270	-3.437231	1.692544
O	2.130314	-3.064180	0.827727
O	0.638925	-2.492097	2.752848
H	-1.635613	-0.321780	-4.333490
H	0.458335	0.606396	-2.826533
H	-1.336083	0.459499	4.495911
H	-3.492820	-0.336837	2.992963
H	0.735882	3.196251	-0.634577
H	3.007817	4.068498	-1.001701
H	4.967752	2.550824	-0.849819
H	4.622353	0.146038	-0.330013
H	2.363743	-0.734937	0.045884
H	-4.988808	1.203246	0.743968
H	-7.384647	0.764859	1.074009

H	-8.262513	-1.556343	0.942620
H	-6.698583	-3.431290	0.487770
H	-4.306299	-2.999674	0.165458
H	1.539489	0.033257	2.954594
H	0.920279	1.303345	4.040297
H	1.399112	1.716875	2.380776
H	-3.937860	-0.991336	-3.921372
H	-3.948067	-2.131982	-2.558393
H	-4.688442	-0.532478	-2.378771
H	-1.833401	-3.229152	-0.416141
H	-1.997894	-2.651183	1.343064
H	-0.738609	-1.865694	0.210649

=== **3B** ===

N	-1.373771	-0.553120	-2.779940
C	-0.231724	-0.399474	-3.550925
C	0.750936	0.007563	-2.713661
N	0.316528	0.819677	2.358857
C	-0.826068	0.728816	3.124479
C	-1.828961	0.350783	2.286973
C	-3.400470	0.670891	-0.462005
C	1.759806	1.930014	-0.412253
C	0.997334	3.108181	-0.476804
C	1.585202	4.350197	-0.706816
C	2.965872	4.446814	-0.880040
C	3.744517	3.293703	-0.817784
C	3.146752	2.053728	-0.584267
C	-3.216247	1.992661	-0.907083
C	-4.281531	2.874669	-1.077756
C	-5.584156	2.455723	-0.805704
C	-5.801132	1.151951	-0.367072
C	-4.723237	0.278487	-0.201698
C	1.649056	1.111096	2.889242
C	-2.655454	-1.015084	-3.307303
N	0.207853	0.089784	-1.441901
B	1.022638	0.509046	-0.182143
C	0.015812	0.489988	1.075603
N	-1.295121	0.201854	1.025975
B	-2.129092	-0.335450	-0.234212
C	-1.095808	-0.258172	-1.487019
C	-2.532654	-1.884730	0.066953
O	0.987483	-2.731340	-0.198514
C	3.675646	-2.610240	-0.266341
F	3.823430	-3.904474	0.021010
F	4.707203	-1.931022	0.239123
F	3.663109	-2.458489	-1.593028
S	2.051586	-2.010938	0.480653
O	2.169996	-0.523070	0.011201
O	2.200010	-2.119992	1.924255
H	-0.228634	-0.587434	-4.610713
H	1.779861	0.252069	-2.910378
H	-0.824075	0.938558	4.180240
H	-2.873856	0.188159	2.484745
H	-0.079337	3.059104	-0.341840
H	0.968451	5.241713	-0.749721
H	3.428514	5.411174	-1.059326
H	4.819749	3.357375	-0.948173
H	3.770249	1.168808	-0.530085
H	-2.213051	2.345105	-1.131416
H	-4.098340	3.886903	-1.423765
H	-6.417837	3.137064	-0.936632
H	-6.809580	0.812056	-0.154072
H	-4.923632	-0.733239	0.135356
H	2.182152	0.179703	3.085248
H	1.536894	1.677921	3.812889
H	2.205714	1.707588	2.169516
H	-2.766097	-0.652145	-4.329303
H	-2.698901	-2.105794	-3.302373
H	-3.462660	-0.614090	-2.699476
H	-3.126874	-2.329630	-0.738826
H	-3.133538	-1.975367	0.979605
H	-1.647823	-2.513440	0.200671

=== **3A** ===

B	-2.182572	-1.203385	-0.052388
B	1.023351	-0.653655	0.008066
C	-2.571015	-2.781343	-0.215786
C	-3.462218	-0.206449	0.131098
C	-4.772667	-0.685771	0.289663
C	-5.857590	0.175555	0.472854
C	-5.657021	1.553343	0.501290
C	-4.365459	2.058259	0.346552
C	-3.289924	1.190384	0.165701
C	-1.228724	-0.761294	-1.294213
C	-2.947610	-0.743151	-3.132059
C	-0.481102	-0.337485	-3.365000
C	0.582641	-0.333226	-2.528095
C	0.068490	-0.859104	1.289700

C	1.798241	-0.652151	3.111738
C	-0.662872	-1.095603	3.380901
C	-1.723981	-1.207400	2.537527
C	2.172316	-1.779667	-0.145618
C	1.810448	-3.136059	-0.202889
C	2.762366	-4.142118	-0.352333
C	4.114974	-3.814736	-0.448699
C	4.499277	-2.477391	-0.394183
C	3.538307	-1.475696	-0.242945
C	2.549737	3.120163	-0.427845
F	2.548271	2.851113	-1.736060
F	3.755352	2.842867	0.071901
F	2.291822	4.416544	-0.250992
N	-1.598469	-0.603774	-2.588380
N	0.110235	-0.591625	-1.252070
N	0.449696	-0.887928	2.593328
N	-1.258736	-1.060508	1.249368
O	1.785904	0.703221	0.103545
O	-0.035729	2.403798	-0.228769
O	1.356309	2.408182	1.870668
S	1.217394	2.117336	0.451676
H	-1.684155	-3.414998	-0.327810
H	-3.207425	-2.968888	-1.087754
H	-3.118175	-3.155798	0.657116
H	-4.960061	-1.754572	0.268239
H	-6.856814	-0.230829	0.592992
H	-6.495685	2.226716	0.642561
H	-4.195206	3.129754	0.368080
H	-2.296264	1.612139	0.051722
H	-3.097747	-1.750476	-3.526288
H	-3.081011	-0.016933	-3.934228
H	-3.675027	-0.544074	-2.349656
H	-0.550092	-0.169089	-4.425848
H	1.625807	-0.159465	-2.725161
H	2.528872	-1.098896	2.440538
H	1.878308	-1.124512	4.090305
H	1.983831	0.418960	3.196842
H	-0.602552	-1.134973	4.454860
H	-2.767175	-1.363091	2.749779
H	0.763554	-3.416435	-0.130801
H	2.450409	-5.180437	-0.392509
H	4.859602	-4.594708	-0.564274
H	5.548362	-2.210607	-0.467938
H	3.857781	-0.440956	-0.197793

Formation of **4**

=== **HBpin** ===

B	1.040554	-2.152497	-1.180585
C	2.811280	-1.668760	0.181719
C	4.071888	-2.298765	-0.419908
C	3.183815	-0.458390	1.031666
C	1.853755	-2.713831	0.880484
C	2.542610	-3.955816	1.436892
C	0.942095	-2.094000	1.945197
O	1.999677	-1.208631	-0.945236
O	0.983640	-3.123671	-0.221547
H	0.326863	-2.130692	-2.125101
H	3.829815	-3.159527	-1.046636
H	4.769664	-2.618963	0.357557
H	4.571569	-1.556705	-1.045947
H	3.819523	0.212244	0.449590
H	3.740518	-0.765181	1.921780
H	2.303400	0.101345	1.346147
H	1.793843	-4.623666	1.868315
H	3.250710	-3.686150	2.225686
H	3.075756	-4.504290	0.660851
H	0.427082	-1.209918	1.564055
H	1.503488	-1.811664	2.839054
H	0.185884	-2.827803	2.231223

=== **RC** ===

B	-1.988350	0.148304	0.321433
B	0.347306	1.834073	-0.637200
B	2.605692	-2.484056	-1.782302
C	-3.186551	-0.686368	0.929962
C	-3.119203	-2.083327	1.081311
C	-4.175592	-2.817117	1.622020
C	-5.337793	-2.168652	2.035615
C	-5.430303	-0.782890	1.906867
C	-4.369347	-0.058590	1.364639
C	-1.511385	0.171689	-1.095711
C	-3.115410	-1.361373	-2.316624
C	-1.207500	-0.083369	-3.314817
C	-0.255347	0.776743	-2.882277
C	-0.127652	1.807294	0.779405
C	1.467687	3.354734	1.994329
C	-0.432033	2.067761	2.998905
C	-1.381112	1.202275	2.569034
C	1.524263	2.693446	-1.251966

C	1.406362	4.085180	-1.420753
C	2.435623	4.849043	-1.972038
C	3.619487	4.235626	-2.378075
C	3.760135	2.855731	-2.231876
C	2.727070	2.098633	-1.679064
C	3.737236	-2.112205	0.170139
C	5.119799	-2.763409	0.057646
C	3.781789	-0.950592	1.157472
C	2.566921	-3.147013	0.405341
C	2.972537	-4.432263	1.120058
C	1.332850	-2.535920	1.075529
N	-1.984272	-0.455936	-2.245571
N	-0.424464	0.954967	-1.503857
N	0.342497	2.441722	1.929014
N	-1.213062	1.020873	1.189801
O	3.421099	-1.581493	-1.157731
O	2.173025	-3.487144	-0.963377
H	2.308772	-2.405252	-2.925594
H	-2.218976	-2.606961	0.774331
H	-4.089623	-3.894043	1.724320
H	-6.161411	-2.735564	2.455865
H	-6.330757	-0.267432	2.224674
H	-4.465325	1.018925	1.267142
H	-2.893949	-2.316574	-1.833353
H	-3.350205	-1.541144	-3.366001
H	-3.986820	-0.923285	-1.827310
H	-1.396163	-0.457655	-4.307597
H	0.520474	1.284204	-3.426034
H	2.343022	2.918461	1.510022
H	1.699201	3.546057	3.042552
H	1.242513	4.303400	1.499902
H	-0.243848	2.443439	3.991417
H	-2.153009	0.692319	3.116213
H	0.486564	4.580127	-1.123037
H	2.310898	5.920595	-2.090034
H	4.421931	4.825414	-2.808042
H	4.676342	2.367002	-2.546966
H	2.864344	1.026708	-1.572045
H	5.117502	-3.587311	-0.658969
H	5.463051	-3.142760	1.023111
H	5.834427	-2.015149	-0.291285
H	4.601289	-0.279094	0.892105
H	3.957972	-1.314691	2.173797
H	2.856122	-0.376064	1.147033
H	2.104797	-5.090184	1.203415
H	3.332041	-4.217004	2.130372
H	3.751605	-4.968612	0.579006
H	1.006597	-1.625298	0.569722
H	1.521808	-2.301499	2.125721
H	0.515872	-3.258817	1.029173

=== **TS-4** ===

B	-1.764448	-0.381661	0.088384
B	0.539337	1.290564	-0.463364
B	1.179551	-0.624614	0.124555
C	-2.844618	-1.469924	0.475397
C	-2.579730	-2.842936	0.336016
C	-3.538894	-3.807727	0.642892
C	-4.797879	-3.423259	1.101375
C	-5.086796	-2.067659	1.251717
C	-4.121617	-1.109115	0.943590
C	-1.478006	0.079531	-1.352330
C	-3.169470	-1.099313	-2.809654
C	-1.268094	0.365819	-3.558248
C	-0.269681	1.059368	-2.949033
C	-0.237989	1.420867	0.865059
C	1.219561	2.854935	2.342947
C	-0.809495	1.552701	3.035274
C	-1.679899	0.704411	2.443824
C	1.661572	2.328429	-0.940037
C	1.428999	3.716070	-0.878202
C	2.356222	4.645286	-1.349655
C	3.554153	4.209816	-1.914563
C	3.803661	2.841868	-2.009829
C	2.869939	1.920541	-1.532666
C	2.981884	-2.070084	-0.088553
C	2.392133	-3.488481	-0.093801
C	4.321898	-2.069405	-0.821926
C	3.002422	-1.413933	1.347390
C	3.135628	-2.404172	2.504858
C	4.058987	-0.309796	1.496521
N	-2.019967	-0.237170	-2.570682
N	-0.404022	0.880403	-1.583070
N	0.076933	1.995597	2.067893
N	-1.339547	0.626078	1.093583
O	2.054837	-1.219869	-0.806770
O	1.700144	-0.792211	1.424178
H	-0.093421	-0.948820	0.023173

H	-1.601225	-3.158859	-0.010590
H	-3.301322	-4.860282	0.529498
H	-5.545107	-4.171513	1.342099
H	-6.063875	-1.756746	1.606670
H	-4.373853	-0.058716	1.058753
H	-2.891486	-2.153772	-2.743300
H	-3.558611	-0.892306	-3.806647
H	-3.946694	-0.898238	-2.074485
H	-1.515349	0.265350	-4.601485
H	0.515060	1.662767	-3.368893
H	2.112424	2.451927	1.868161
H	1.374667	2.883748	3.421438
H	1.054420	3.870227	1.977536
H	-0.742139	1.883397	4.057786
H	-2.495151	0.142210	2.861427
H	0.493010	4.078959	-0.462839
H	2.141124	5.707142	-1.283740
H	4.279140	4.927555	-2.283376
H	4.727245	2.490429	-2.458939
H	3.070963	0.859534	-1.627071
H	1.430772	-3.516175	0.423095
H	3.062756	-4.210644	0.379633
H	2.229768	-3.796695	-1.129293
H	4.193698	-2.506936	-1.815117
H	5.063386	-2.666513	-0.282368
H	4.714267	-1.060100	-0.945837
H	3.115141	-1.860614	3.452635
H	4.082488	-2.950226	2.448739
H	2.316383	-3.122704	2.513520
H	3.987806	0.424682	0.692840
H	5.073264	-0.718218	1.508309
H	3.891809	0.207822	2.444497

=== **4** ===

B	-1.963473	-1.355108	0.416908
B	0.802583	0.308832	-0.073117
B	2.326328	-0.489110	0.129519
C	-2.010549	-2.905435	0.905222
C	-0.844407	-3.679288	1.040027
C	-0.883188	-5.003717	1.472590
C	-2.105127	-5.601309	1.784821
C	-3.278284	-4.860313	1.659783
C	-3.223773	-3.533805	1.225757
C	-1.180872	-1.164876	-0.972192
C	-2.752801	-2.486569	-2.400427
C	-0.643051	-1.309752	-3.139073
C	0.308572	-0.574746	-2.508358
C	-0.074333	0.240411	1.285104
C	1.344093	1.743954	2.722925
C	-0.775022	0.560950	3.388607
C	-1.650547	-0.270644	2.769418
C	0.953203	1.858522	-0.588755
C	-0.155212	2.725989	-0.577868
C	-0.074138	4.038334	-1.039972
C	1.133691	4.531999	-1.534616
C	2.247935	3.697292	-1.563296
C	2.151974	2.383946	-1.096846
C	4.161818	-1.808424	-0.387131
C	3.713172	-3.274349	-0.423163
C	5.375794	-1.615783	-1.292545
C	4.314060	-1.232224	1.072877
C	4.635843	-2.266382	2.148867
C	5.295824	-0.058208	1.170743
N	-1.567636	-1.668287	-2.172900
N	-0.035303	-0.490850	-1.171782
N	0.200066	0.873659	2.458236
N	-1.210209	-0.457888	1.474275
O	3.058690	-1.018708	-0.917907
O	2.983189	-0.694831	1.332365
H	-3.095505	-0.925587	0.297457
H	0.119244	-3.234859	0.804912
H	0.036752	-5.572241	1.566628
H	-2.140368	-6.631824	2.121835
H	-4.234525	-5.313526	1.901585
H	-4.147780	-2.969482	1.134655
H	-2.582056	-3.515435	-2.078464
H	-2.991429	-2.470426	-3.463733
H	-3.591144	-2.077394	-1.837877
H	-0.748070	-1.602409	-4.169625
H	1.197068	-0.108500	-2.894867
H	2.268735	1.178691	2.615223
H	1.258567	2.118829	3.742364
H	1.346257	2.584038	2.028706
H	-0.754355	0.953308	4.390681
H	-2.541769	-0.746948	3.139229
H	-1.108896	2.365915	-0.200800
H	-0.951584	4.676865	-1.015641
H	1.202815	5.553081	-1.894185

H	3.192747	4.066591	-1.949613
H	3.033862	1.752132	-1.142092
H	2.835899	-3.437922	0.205593
H	4.508063	-3.948147	-0.093487
H	3.444593	-3.534821	-1.449041
H	5.154816	-2.014431	-2.285315
H	6.245278	-2.151009	-0.899725
H	5.635407	-0.563117	-1.402964
H	4.691046	-1.774677	3.122866
H	5.601992	-2.742115	1.956296
H	3.871038	-3.040296	2.206415
H	5.071651	0.715578	0.433995
H	6.329065	-0.384683	1.028990
H	5.214870	0.389643	2.163863

=== **4*** ===

B	-1.876241	-1.291987	0.638188
B	0.806264	0.338675	-0.257818
B	2.313370	-0.491937	-0.054730
C	-1.839730	-2.786460	1.275023
C	-0.676044	-3.575710	1.264310
C	-0.645866	-4.849843	1.830021
C	-1.790845	-5.378022	2.427777
C	-2.959813	-4.620175	2.450132
C	-2.976759	-3.345898	1.878793
C	-1.231983	-1.217436	-0.830664
C	-2.895208	-2.690667	-1.981141
C	-0.903828	-1.537822	-3.020287
C	0.084019	-0.734315	-2.549285
C	0.046181	0.410102	1.172037
C	1.511350	2.124056	2.298094
C	-0.494537	0.961406	3.274783
C	-1.399762	0.055985	2.824270
C	0.919526	1.832953	-0.920230
C	-0.241448	2.606872	-1.106823
C	-0.203197	3.873693	-1.685746
C	1.013810	4.414378	-2.103363
C	2.180348	3.673732	-1.932209
C	2.127084	2.405377	-1.347964
C	4.535311	-0.907644	0.479737
C	5.430910	-0.231199	-0.565562
C	5.291348	-1.040456	1.799323
C	3.848674	-2.229428	-0.037968
C	4.606526	-2.965601	-1.139798
C	3.482464	-3.208504	1.083466
N	-1.720322	-1.828426	-1.941234
N	-0.129742	-0.542846	-1.197361
N	0.403162	1.171344	2.243071
N	-1.057063	-0.275314	1.528455
O	3.390353	-0.028293	0.688056
O	2.595995	-1.732410	-0.592466
H	-3.024756	-0.893428	0.588030
H	0.226419	-3.188183	0.799376
H	0.269031	-5.433648	1.804595
H	-1.771381	-6.369300	2.868063
H	-3.857621	-5.020925	2.910194
H	-3.898133	-2.770205	1.899708
H	-2.638953	-3.710331	-1.687020
H	-3.297752	-2.692284	-2.994265
H	-3.648520	-2.305630	-1.295834
H	-1.100503	-1.918439	-4.007799
H	0.909772	-0.276474	-3.064771
H	2.403048	1.679683	1.861181
H	1.703935	2.362135	3.344314
H	1.256257	3.036833	1.757560
H	-0.404624	1.466724	4.220981
H	-2.249096	-0.385231	3.316187
H	-1.202501	2.206200	-0.794970
H	-1.120307	4.440328	-1.812430
H	1.049830	5.399962	-2.555114
H	3.133916	4.081907	-2.252243
H	3.053279	1.857038	-1.212378
H	4.915162	-0.115956	-1.520877
H	6.350217	-0.797521	-0.734498
H	5.703658	0.763455	-0.206092
H	5.704873	-0.069622	2.082751
H	6.122405	-1.745021	1.702555
H	4.640276	-1.378916	2.605007
H	4.030441	-3.838519	-1.454784
H	5.579023	-3.313942	-0.779418
H	4.764206	-2.333756	-2.013500
H	2.910836	-2.719120	1.874296
H	4.371724	-3.663443	1.527019
H	2.863667	-4.005692	0.666193

Formation of **5a**

=== **Ph_2SiH_2** ===

C	-3.690043	1.168288	0.524498

C	-4.920998	0.674614	0.983991
C	-4.983554	-0.424549	1.840609
C	-3.810912	-1.051736	2.255287
C	-2.576908	-0.575941	1.811610
C	-2.519496	0.522486	0.957140
C	-2.422472	3.967176	-0.068181
C	-2.378619	4.369349	1.277065
C	-1.517113	5.379746	1.698821
C	-0.676550	6.008921	0.780789
C	-0.701125	5.622172	-0.557492
C	-1.565289	4.610144	-0.974952
Si	-3.620658	2.635397	-0.656428
H	-3.177581	2.211390	-2.013345
H	-5.844069	1.153182	0.672089
H	-5.945746	-0.788287	2.184494
H	-3.856675	-1.906060	2.921710
H	-1.661019	-1.059935	2.132951
H	-1.549206	0.882269	0.628320
H	-3.019507	3.885421	2.007716
H	-1.499018	5.674643	2.742425
H	-0.004315	6.794391	1.108249
H	-0.046520	6.104881	-1.275031
H	-1.568314	4.317866	-2.020523
H	-5.004214	3.174820	-0.768428

=== **RC** ===

B	1.951705	-0.097201	0.242190
B	0.406606	-2.400300	-0.990627
C	2.786403	1.138413	0.771823
C	4.157233	1.028450	1.067588
C	4.899759	2.116190	1.528410
C	4.287405	3.356170	1.702904
C	2.931741	3.497568	1.408297
C	2.197720	2.404428	0.948618
C	1.489048	-1.289992	1.013262
C	2.258478	-0.828923	3.380492
C	1.014754	-2.849138	2.572182
C	0.504337	-3.296055	1.399133
C	0.850360	-1.197189	-1.757806
C	-0.002271	-1.620829	-4.101296
C	1.297982	0.372083	-3.311777
C	1.835654	0.805502	-2.146255
C	-0.381222	-3.661938	-1.533601
C	0.268404	-4.684450	-2.247870
C	-0.421023	-5.800255	-2.724120
C	-1.789767	-5.926475	-2.493007
C	-2.458547	-4.931986	-1.780111
C	-1.761005	-3.819974	-1.308000
C	-3.594156	1.101418	0.463309
C	-4.826616	0.868685	1.094720
C	-4.975937	-0.154259	2.031173
C	-3.890980	-0.967762	2.350979
C	-2.658641	-0.756753	1.731979
C	-2.513427	0.267721	0.798809
C	-2.351869	3.916538	-0.115916
C	-2.308014	4.214899	1.255211
C	-1.566838	5.294221	1.734129
C	-0.850863	6.097618	0.847899
C	-0.876033	5.814542	-0.516917
C	-1.617802	4.733523	-0.990752
N	1.610415	-1.630978	2.359758
N	0.778636	-2.339127	0.413084
N	0.690819	-0.839537	-3.093963
N	1.562786	-0.150311	-1.159752
Si	-3.404337	2.498014	-0.784079
H	-2.767046	2.011728	-2.038065
H	4.655011	0.073402	0.927600
H	5.955985	1.996780	1.746959
H	4.861125	4.203935	2.061723
H	2.442865	4.457173	1.538786
H	1.142608	2.539355	0.730721
H	2.067821	-1.283857	4.352810
H	1.860445	0.187525	3.379064
H	3.337888	-0.773680	3.217832
H	0.998342	-3.307524	3.547531
H	-0.026947	-4.202933	1.173117
H	0.526145	-2.553779	-4.313121
H	-0.064083	-1.030958	-5.015990
H	-1.012102	-1.867573	-3.768439
H	1.298835	0.836910	-4.284086
H	2.381978	1.704422	-1.925310
H	1.335934	-4.607466	-2.432113
H	0.110218	-6.571321	-3.272561
H	-2.329577	-6.791577	-2.862730
H	-3.524080	-5.020191	-1.594878
H	-2.301477	-3.054090	-0.759806
H	-5.683170	1.489969	0.851807
H	-5.937221	-0.316306	2.506825

H	-4.004770	-1.766035	3.076476
H	-1.812580	-1.391504	1.971256
H	-1.544843	0.410366	0.328914
H	-2.852190	3.594485	1.960321
H	-1.545803	5.506112	2.797716
H	-0.273928	6.937770	1.218903
H	-0.315290	6.431885	-1.210449
H	-1.619614	4.523827	-2.056259
H	-4.770777	2.989788	-1.116202

=== **TS-5a** ===

B	1.606689	0.227883	0.449497
B	-0.175973	-1.538135	-1.078360
C	2.552370	1.231951	1.237041
C	3.924238	1.314407	0.930993
C	4.786026	2.155232	1.634346
C	4.297324	2.946094	2.673642
C	2.943173	2.887249	2.996607
C	2.089106	2.043336	2.285964
C	1.282046	-1.192261	0.945022
C	2.419678	-1.416372	3.188834
C	0.880393	-3.072576	2.092609
C	0.180764	-3.143103	0.932948
C	0.657027	-0.448990	-1.757022
C	-0.166138	-0.393423	-4.152960
C	1.511724	1.141845	-3.096083
C	2.066362	1.303358	-1.872884
C	-1.035258	-2.644197	-1.833219
C	-0.420540	-3.462278	-2.800747
C	-1.105626	-4.496022	-3.438030
C	-2.438644	-4.749412	-3.118055
C	-3.069753	-3.965159	-2.154537
C	-2.375633	-2.932217	-1.523227
C	-2.531639	0.320593	1.554023
C	-3.914085	0.067550	1.573766
C	-4.619826	-0.038523	2.771499
C	-3.954831	0.103383	3.988879
C	-2.582621	0.348365	3.996941
C	-1.885553	0.451249	2.793255
C	-1.586659	2.279240	-0.757737
C	-0.778615	3.280123	-0.194828
C	-0.795440	4.588107	-0.676065
C	-1.619895	4.928145	-1.748289
C	-2.428374	3.952064	-2.328160
C	-2.411135	2.647481	-1.833913
N	1.563491	-1.872661	2.102042
N	0.426414	-1.973670	0.220196
N	0.642296	0.068713	-3.033791
N	1.553044	0.312807	-1.038099
Si	-1.602624	0.482813	-0.107281
H	-0.045879	0.512363	0.533978
H	4.331709	0.699602	0.133534
H	5.838707	2.190987	1.373629
H	4.963787	3.602306	3.222534
H	2.549120	3.502265	3.798856
H	1.037459	2.021351	2.551374
H	2.664513	-2.272030	3.817985
H	1.918151	-0.658156	3.794317
H	3.339451	-0.990693	2.790988
H	0.962355	-3.778163	2.901916
H	-0.459450	-3.917373	0.550475
H	0.297490	-1.243210	-4.660269
H	-0.278067	0.428932	-4.859856
H	-1.152020	-0.692506	-3.802800
H	1.650421	1.705152	-4.003232
H	2.758604	2.045267	-1.518913
H	0.622375	-3.293762	-3.053500
H	-0.598638	-5.106491	-4.178198
H	-2.977271	-5.551712	-3.610670
H	-4.105389	-4.156060	-1.893148
H	-2.891718	-2.335172	-0.780690
H	-4.447844	-0.043660	0.634767
H	-5.687711	-0.230730	2.755863
H	-4.500924	0.022148	4.922594
H	-2.057069	0.457928	4.940234
H	-0.816290	0.634710	2.819649
H	-0.118376	3.038065	0.631018
H	-0.162944	5.341274	-0.217477
H	-1.633384	5.944848	-2.126416
H	-3.076278	4.207434	-3.160611
H	-3.055117	1.901827	-2.290072
H	-2.632477	-0.090538	-1.044226

=== **5a** ===

B	1.680262	0.427147	1.519154
B	-0.572632	-0.601185	-0.597733
C	3.270615	0.094093	1.578370
C	3.850862	-0.934549	0.815260

C	5.218448	-1.203110	0.857623
C	6.055079	-0.440888	1.673748
C	5.508808	0.584264	2.443221
C	4.136780	0.840164	2.392961
C	0.767178	-0.887520	1.637703
C	1.608880	-1.666576	3.862272
C	-0.112109	-2.796756	2.403066
C	-0.669430	-2.523544	1.196003
C	0.411128	0.665743	-0.774486
C	-0.280071	1.461584	-3.082421
C	1.428880	2.494462	-1.577702
C	1.942976	2.224280	-0.351926
C	-0.447749	-1.655326	-1.840241
C	0.809792	-1.898016	-2.426530
C	0.987068	-2.827590	-3.449142
C	-0.101277	-3.558224	-3.926083
C	-1.357431	-3.347032	-3.364702
C	-1.520891	-2.410953	-2.340690
C	-2.723596	0.836915	1.527588
C	-3.789607	0.446814	2.357108
C	-3.981096	1.015827	3.615450
C	-3.104961	1.996030	4.078957
C	-2.037676	2.397107	3.277727
C	-1.851109	1.821760	2.020819
C	-3.306370	1.237652	-1.473590
C	-3.334201	2.628471	-1.279049
C	-3.870147	3.487487	-2.239270
C	-4.399897	2.972881	-3.421557
C	-4.395496	1.594084	-3.631637
C	-3.855737	0.742447	-2.668895
N	0.774997	-1.769852	2.669152
N	-0.121645	-1.338665	0.733653
N	0.481945	1.519884	-1.835572
N	1.313009	1.095408	0.131961
Si	-2.532128	0.052767	-0.194249
H	1.388565	1.205713	2.404810
H	3.220533	-1.545261	0.173738
H	5.633014	-2.006037	0.256368
H	7.119598	-0.646162	1.709931
H	6.149454	1.182450	3.083409
H	3.725185	1.637771	3.005209
H	1.148303	-2.245972	4.662237
H	1.674920	-0.623659	4.167475
H	2.614578	-2.042370	3.666656
H	-0.267172	-3.613053	3.087357
H	-1.403910	-3.070148	0.631428
H	0.406717	1.580992	-3.922201
H	-1.034046	2.248897	-3.104689
H	-0.767764	0.496124	-3.165681
H	1.650494	3.274417	-2.285884
H	2.714884	2.724576	0.206429
H	1.676324	-1.345050	-2.074860
H	1.973618	-2.983561	-3.873729
H	0.030078	-4.281842	-4.723237
H	-2.213742	-3.909605	-3.722495
H	-2.512440	-2.276582	-1.922028
H	-4.484333	-0.313398	2.013868
H	-4.813853	0.695115	4.232696
H	-3.251449	2.441680	5.057009
H	-1.345621	3.153810	3.631829
H	-1.002177	2.145420	1.427952
H	-2.945023	3.051602	-0.358692
H	-3.882489	4.557516	-2.059420
H	-4.821537	3.638057	-4.167280
H	-4.816921	1.182129	-4.542678
H	-3.866060	-0.326855	-2.855878
H	-3.458567	-1.125000	-0.157516

=== **5a*** ===

B	1.716160	0.233601	1.610786
B	-0.471763	-0.634803	-0.647250
C	1.465393	1.154154	2.925019
C	2.537899	1.558561	3.735923
C	2.350566	2.352471	4.869557
C	1.068774	2.765916	5.226148
C	-0.016916	2.381056	4.438620
C	0.184481	1.588898	3.309103
C	0.847631	-1.113446	1.572472
C	1.801618	-2.152540	3.636843
C	0.102721	-3.164913	2.064388
C	-0.503476	-2.753741	0.922215
C	0.529991	0.635755	-0.703479
C	-0.050741	1.598157	-2.980187
C	1.575708	2.520391	-1.325319
C	2.022061	2.171774	-0.093302
C	-0.357825	-1.598327	-1.954982
C	0.889263	-1.811670	-2.573127
C	1.042866	-2.674616	-3.656583

C	-0.057884	-3.367290	-4.160711
C	-1.303211	-3.189254	-3.563737
C	-1.443119	-2.318592	-2.480639
C	-3.163565	1.293147	-1.503087
C	-3.451216	2.630416	-1.178219
C	-4.026645	3.500388	-2.104871
C	-4.337321	3.050431	-3.386669
C	-4.065076	1.727334	-3.733428
C	-3.483391	0.865833	-2.804157
C	-3.689996	-1.090798	0.399157
C	-4.725046	-1.564956	-0.423614
C	-5.667403	-2.480573	0.044507
C	-5.601818	-2.943422	1.357318
C	-4.591217	-2.478763	2.197727
C	-3.651398	-1.565798	1.721807
N	0.940612	-2.136439	2.458745
N	-0.039215	-1.481929	0.627509
N	0.652636	1.560561	-1.699662
N	1.376351	1.010817	0.277895
Si	-2.371995	0.159616	-0.191246
H	2.891246	-0.082245	1.557847
H	3.543796	1.243095	3.473001
H	3.203549	2.647853	5.472374
H	0.916224	3.381908	6.105947
H	-1.019769	2.699217	4.705337
H	-0.676768	1.309041	2.709819
H	1.842110	-3.169968	4.024308
H	1.415830	-1.481965	4.405791
H	2.806015	-1.830897	3.361951
H	0.015156	-4.080610	2.623234
H	-1.217288	-3.260249	0.298214
H	0.669608	1.810583	-3.772106
H	-0.823957	2.366825	-2.968307
H	-0.507336	0.633394	-3.173051
H	1.825555	3.349245	-1.965178
H	2.743139	2.647000	0.548805
H	1.765692	-1.291618	-2.196616
H	2.021361	-2.809983	-4.106135
H	0.055853	-4.038984	-5.004636
H	-2.167545	-3.727915	-3.938545
H	-2.424384	-2.214920	-2.028839
H	-3.224041	2.999536	-0.183237
H	-4.236486	4.527017	-1.823407
H	-4.788650	3.722801	-4.108152
H	-4.302220	1.367187	-4.729096
H	-3.263268	-0.151640	-3.110900
H	-4.816808	-1.202225	-1.442090
H	-6.458315	-2.824700	-0.613762
H	-6.336857	-3.651207	1.724867
H	-4.539046	-2.821479	3.225932
H	-2.881402	-1.210239	2.399351
H	-2.179150	1.059438	0.988480

Formation of **6***

=== **(*p*-FC$_6$H$_4$)$_2$PH** ===

P	2.049465	0.042054	1.200829
C	1.962385	1.885435	1.114068
C	2.917501	-0.401531	-0.381652
C	3.092014	2.632798	1.479575
C	3.068680	4.024053	1.478019
C	1.890328	4.665386	1.126006
C	0.746581	3.968447	0.774700
C	0.793025	2.574568	0.766198
C	2.778507	0.310866	-1.580655
C	3.444177	-0.090741	-2.734889
C	4.253027	-1.216807	-2.676010
C	4.423122	-1.946191	-1.511878
C	3.752022	-1.525480	-0.363803
F	1.856995	6.019835	1.130445
F	4.902766	-1.611345	-3.797207
H	0.716507	-0.189227	0.760839
H	4.004877	2.124668	1.771908
H	3.938284	4.607745	1.753637
H	-0.154140	4.508259	0.509552
H	-0.097576	2.023934	0.484753
H	2.150427	1.193496	-1.617333
H	3.344371	0.451740	-3.667065
H	5.069719	-2.814877	-1.507832
H	3.883562	-2.081770	0.558002

=== **RC** ===

N	-2.545198	-1.222345	2.945686
C	-2.067479	-2.353838	3.559808
C	-1.273939	-3.019960	2.688197
N	-0.265087	-1.626168	-2.141923
C	-0.742757	-0.496655	-2.756628
C	-1.535076	0.172918	-1.885387
C	-3.453379	-0.269056	3.555013

N	-1.241040	-2.296444	1.489591
B	-0.553352	-2.671014	0.262487
C	-0.769063	-1.688820	-0.843841
N	-1.565359	-0.546767	-0.683195
B	-2.251531	-0.171806	0.544689
C	-2.036325	-1.155180	1.650860
P	2.731339	0.223858	0.968691
C	2.597344	2.064630	1.041358
C	3.660854	-0.055250	-0.619256
C	3.669186	2.799975	1.569170
C	3.606648	4.185677	1.684386
C	2.445886	4.830085	1.283132
C	1.357312	4.143402	0.772264
C	1.443182	2.756579	0.649775
C	3.637459	0.823890	-1.709982
C	4.345689	0.541494	-2.875192
C	5.076506	-0.635951	-2.938192
C	5.131823	-1.530127	-1.882722
C	4.424135	-1.225068	-0.720122
C	-3.106578	1.157663	0.579617
C	-2.721069	2.272265	1.346924
C	-3.475078	3.446265	1.371635
C	-4.643059	3.543033	0.617574
C	-5.045995	2.457750	-0.160560
C	-4.286827	1.288531	-0.177204
C	0.295283	-4.004300	0.228153
C	1.484938	-4.135869	0.969493
C	2.233359	-5.312258	0.955574
C	1.809613	-6.402417	0.195677
C	0.630352	-6.304824	-0.540556
C	-0.113046	-5.124293	-0.518548
F	2.374127	6.179022	1.400410
F	5.765028	-0.916410	-4.072530
C	0.650818	-2.576563	-2.745886
H	-2.337027	-2.603071	4.573015
H	-0.741192	-3.946145	2.804005
H	-0.476992	-0.251243	-3.771822
H	-2.073513	1.095231	-2.006515
H	-3.813856	-0.686353	4.495513
H	-2.957851	0.684323	3.755922
H	-4.305683	-0.080829	2.900040
H	1.423373	-0.010710	0.465872
H	4.566019	2.286259	1.899106
H	4.431198	4.761127	2.086772
H	0.467325	4.683712	0.473786
H	0.596292	2.214229	0.245161
H	3.065655	1.742638	-1.652865
H	4.337783	1.216075	-3.722793
H	5.721858	-2.434109	-1.969638
H	4.469806	-1.910854	0.118949
H	-1.809643	2.221940	1.934957
H	-3.148661	4.285986	1.976597
H	-5.232066	4.453532	0.634310
H	-5.954660	2.520947	-0.750292
H	-4.624613	0.453135	-0.783561
H	1.835698	-3.298178	1.565488
H	3.147824	-5.378619	1.536156
H	2.389469	-7.318873	0.181935
H	0.285199	-7.150319	-1.126652
H	-1.037255	-5.074578	-1.086478
H	0.992497	-2.171000	-3.698153
H	1.515307	-2.738350	-2.100104
H	0.166382	-3.540305	-2.921465

=== **TS-6*** ===

N	-2.009497	-0.636289	2.643358
C	-1.543493	-1.601424	3.520681
C	-0.751905	-2.448842	2.824005
N	-0.097014	-2.431946	-2.255176
C	-0.722753	-1.589532	-3.145621
C	-1.374509	-0.635896	-2.427336
C	-2.834207	0.500067	3.038844
N	-0.726207	-2.005639	1.499249
B	0.046551	-2.562379	0.379647
C	-0.366015	-2.001688	-0.974565
N	-1.167440	-0.895410	-1.087515
B	-1.421824	0.005953	0.113472
C	-1.499914	-0.869312	1.398043
P	1.858320	0.155726	0.380572
C	1.800194	1.623848	1.490389
C	2.021359	0.852500	-1.317795
C	1.306968	1.427360	2.794990
C	1.312715	2.446583	3.743848
C	1.821132	3.684666	3.384319
C	2.327700	3.925982	2.117966
C	2.316505	2.894460	1.180313
C	1.287241	1.941300	-1.821515
C	1.406754	2.350752	-3.146712

C	2.261582	1.650337	-3.984217
C	3.007506	0.571463	-3.539285
C	2.883551	0.185682	-2.205790
C	-2.392965	1.253920	-0.112337
C	-2.015696	2.554347	0.263195
C	-2.873011	3.643963	0.103962
C	-4.140177	3.462911	-0.447352
C	-4.540557	2.184247	-0.834792
C	-3.677606	1.101356	-0.666463
C	0.984725	-3.803699	0.633507
C	2.384509	-3.685200	0.598787
C	3.210406	-4.788893	0.813362
C	2.656181	-6.041973	1.068972
C	1.269754	-6.183079	1.115808
C	0.448518	-5.076109	0.903501
F	1.826208	4.689302	4.303948
F	2.375503	2.038951	-5.282858
C	0.667030	-3.615143	-2.628323
H	-1.831917	-1.609458	4.558199
H	-0.215775	-3.325227	3.140471
H	-0.650154	-1.732135	-4.210407
H	-1.950017	0.208234	-2.762491
H	-3.323491	0.259672	3.982650
H	-2.222854	1.396865	3.164606
H	-3.589389	0.694507	2.280636
H	-0.095241	0.347182	0.334683
H	0.922749	0.452975	3.081061
H	0.937436	2.289609	4.748278
H	2.727721	4.903400	1.875380
H	2.726349	3.085107	0.196083
H	0.597214	2.470415	-1.176474
H	0.839084	3.188272	-3.534526
H	3.677272	0.060178	-4.220374
H	3.476818	-0.646723	-1.842143
H	-1.034390	2.715978	0.697929
H	-2.549884	4.634155	0.408015
H	-4.808382	4.307654	-0.574672
H	-5.526061	2.030443	-1.262387
H	-4.017039	0.112941	-0.964142
H	2.829102	-2.711413	0.421846
H	4.288230	-4.667917	0.786116
H	3.297946	-6.900476	1.234161
H	0.828505	-7.153715	1.316198
H	-0.629332	-5.208571	0.940027
H	0.707089	-3.667490	-3.715674
H	1.682175	-3.558782	-2.235823
H	0.193840	-4.519968	-2.242797

=== **6*** ===

N	-1.515326	0.008774	2.991500
C	-0.874854	-0.995052	3.697478
C	-0.055986	-1.627619	2.818841
N	-0.030331	-0.997018	-2.259279
C	-0.870366	-0.174948	-2.987462
C	-1.508278	0.623593	-2.093419
C	-2.474694	0.949625	3.565606
N	-0.200281	-1.007628	1.589236
B	0.644011	-1.342573	0.306044
C	-0.150615	-0.700675	-0.933724
N	-1.062047	0.287959	-0.832330
B	-1.616689	0.940011	0.505152
C	-1.102606	-0.011204	1.699611
P	2.509662	-0.449936	0.618226
C	2.205498	1.261544	1.269024
C	3.246806	-0.167454	-1.064677
C	1.792168	1.358744	2.610167
C	1.611330	2.587351	3.237939
C	1.873641	3.742975	2.520513
C	2.320331	3.701423	1.212360
C	2.483123	2.460749	0.596094
C	2.689166	0.624245	-2.083079
C	3.318120	0.775693	-3.317257
C	4.516071	0.113016	-3.536792
C	5.106142	-0.681404	-2.568874
C	4.466770	-0.806906	-1.335794
C	-3.236412	1.034741	0.427608
C	-3.890315	2.276073	0.392787
C	-5.281426	2.376030	0.316635
C	-6.062475	1.223024	0.273844
C	-5.440478	-0.025980	0.304339
C	-4.050932	-0.110569	0.379038
C	0.801168	-2.959340	0.190923
C	2.002281	-3.662741	0.361499
C	2.063013	-5.055339	0.264527
C	0.910992	-5.791023	0.001311
C	-0.302328	-5.120829	-0.160373
C	-0.348060	-3.731774	-0.066712
F	1.705833	4.948575	3.126773

F	5.129298	0.251112	-4.739450
C	0.778130	-2.053178	-2.867860
H	-1.058380	-1.165221	4.744419
H	0.608477	-2.461854	2.959795
H	-0.937546	-0.235102	-4.060146
H	-2.251928	1.385917	-2.247133
H	-2.388337	0.909911	4.651000
H	-2.245610	1.958390	3.225291
H	-3.491899	0.695950	3.266696
H	-1.134841	2.044261	0.635300
H	1.626550	0.457185	3.188363
H	1.294492	2.653695	4.271927
H	2.537115	4.623391	0.686406
H	2.846779	2.446178	-0.421786
H	1.749169	1.136334	-1.920891
H	2.893651	1.394098	-4.099135
H	6.046901	-1.176864	-2.775379
H	4.929639	-1.410028	-0.562852
H	-3.295392	3.184146	0.428651
H	-5.754347	3.352528	0.290883
H	-7.143255	1.294750	0.216523
H	-6.039346	-0.930505	0.269847
H	-3.589732	-1.095023	0.399635
H	2.910998	-3.114490	0.585507
H	3.012048	-5.563771	0.400305
H	0.954135	-6.872276	-0.072158
H	-1.210357	-5.681194	-0.358356
H	-1.306094	-3.234563	-0.195337
H	1.060078	-1.736170	-3.871624
H	1.678908	-2.217588	-2.285535
H	0.212791	-2.985225	-2.918693

=== **6** ===

N	-0.848540	-0.326128	2.758381
C	0.006125	-1.210024	3.393314
C	0.899828	-1.623439	2.461103
N	1.012652	-0.083605	-2.507372
C	0.121412	0.786781	-3.106937
C	-0.762999	1.162545	-2.150447
C	-1.986628	0.337209	3.389858
N	0.594563	-0.988857	1.266457
B	1.356501	-1.229534	-0.093709
C	0.672307	-0.244240	-1.194866
N	-0.415143	0.521147	-0.980870
B	-1.283677	0.619129	0.334305
C	-0.482897	-0.199649	1.458002
P	3.337746	-0.682210	0.018226
C	3.282459	1.049093	0.683576
C	4.190210	-1.607704	1.385309
C	3.629559	2.095738	-0.182989
C	3.621117	3.426790	0.235075
C	3.278308	3.700256	1.547548
C	2.935932	2.700439	2.445363
C	2.931340	1.380095	2.002471
C	4.006058	-2.988106	1.577571
C	4.760420	-3.706273	2.504836
C	5.719241	-3.037145	3.245460
C	5.950855	-1.681769	3.080871
C	5.190055	-0.979735	2.148904
C	-1.611301	2.163012	0.700949
C	-2.929738	2.645299	0.685031
C	-3.234294	3.973275	0.991565
C	-2.214469	4.862825	1.323971
C	-0.893529	4.413426	1.342113
C	-0.603782	3.084680	1.033750
C	1.165590	-2.781716	-0.571076
C	2.129191	-3.507635	-1.291518
C	1.899427	-4.811897	-1.732152
C	0.682773	-5.436340	-1.467453
C	-0.296059	-4.740532	-0.759693
C	-0.054060	-3.438770	-0.323961
F	3.277893	4.989789	1.972705
F	6.455661	-3.728291	4.154834
C	2.059089	-0.777184	-3.258517
H	-0.097645	-1.461080	4.434924
H	1.723590	-2.306840	2.552739
H	0.205604	1.060592	-4.144443
H	-1.599249	1.837990	-2.196663
H	-2.138957	-0.103693	4.374345
H	-1.802389	1.406777	3.489676
H	-2.882421	0.190044	2.787161
H	-2.324172	0.017744	0.132340
H	3.926994	1.867287	-1.200422
H	3.887968	4.235553	-0.434074
H	2.670576	2.959162	3.463317
H	2.662909	0.597758	2.702072
H	3.267553	-3.524738	0.996550
H	4.608964	-4.769174	2.649804

H	6.716371	-1.189326	3.668394
H	5.386881	0.076935	2.017940
H	-3.735948	1.964193	0.427128
H	-4.264904	4.312915	0.968735
H	-2.444317	5.895491	1.563642
H	-0.090671	5.098089	1.595860
H	0.431455	2.759474	1.051801
H	3.091700	-3.054219	-1.506465
H	2.674622	-5.340717	-2.277181
H	0.500334	-6.450511	-1.805539
H	-1.249947	-5.211648	-0.546095
H	-0.837991	-2.923600	0.221819
H	2.302051	-0.177937	-4.136052
H	2.948727	-0.883085	-2.641558
H	1.711368	-1.762078	-3.573949

Formation of **8** and **8***

=== **PhHC=CH$_2$** ===

C	1.150351	-1.421605	-0.046717
C	0.229686	-2.390143	-0.065610
H	1.506317	-1.013274	0.891375
H	1.582133	-1.001607	-0.948330
H	-0.146740	-2.752858	0.888492
C	-0.357358	-3.049944	-1.242592
C	-1.303983	-4.067120	-1.047437
C	-0.017441	-2.712079	-2.563267
C	-1.890379	-4.725540	-2.125305
H	-1.580935	-4.342700	-0.034931
C	-0.601332	-3.367484	-3.639981
H	0.708467	-1.929710	-2.750817
C	-1.541047	-4.378305	-3.427636
H	-2.619045	-5.508377	-1.947004
H	-0.324150	-3.090156	-4.651004
H	-1.994633	-4.887437	-4.270304

=== **RC** ===

B	-2.789078	0.431973	0.119740
B	-0.151035	1.775643	-0.549461
C	-4.140165	-0.249431	0.584111
C	-4.201615	-1.616324	0.912287
C	-5.388541	-2.218007	1.330363
C	-6.555963	-1.462857	1.436608
C	-6.523066	-0.104540	1.126037
C	-5.330881	0.488677	0.708906
C	-2.393804	0.836974	-1.262827
C	-4.319307	0.048274	-2.701144
C	-2.243427	1.218941	-3.479070
C	-1.101927	1.688338	-2.919494
C	-0.543771	1.366403	0.832879
C	1.408954	2.099766	2.264685
C	-0.696860	0.989513	3.049937
C	-1.839687	0.523399	2.490827
C	1.187805	2.482083	-1.011580
C	1.219256	3.854096	-1.322617
C	2.391445	4.486842	-1.736031
C	3.574171	3.757622	-1.854998
C	3.570405	2.394779	-1.563153
C	2.392528	1.770006	-1.151174
N	-3.038845	0.697829	-2.489701
N	-1.169447	1.459964	-1.538701
N	0.099762	1.508254	2.060271
N	-1.769631	0.746181	1.109399
H	-3.305980	-2.224914	0.830760
H	-5.402874	-3.276228	1.569737
H	-7.480892	-1.927927	1.760134
H	-7.424215	0.493875	1.211741
H	-5.323817	1.550348	0.480143
H	-4.303217	-0.971070	-2.309376
H	-4.518266	0.014141	-3.772407
H	-5.125558	0.593752	-2.205635
H	-2.550534	1.213489	-4.512137
H	-0.249790	2.162186	-3.372297
H	2.194218	1.489922	1.810979
H	1.591988	2.175631	3.336706
H	1.452586	3.097674	1.824583
H	-0.390286	0.995820	4.083121
H	-2.698361	0.063500	2.945564
H	0.309505	4.440485	-1.231786
H	2.383015	5.548035	-1.962490
H	4.488181	4.246255	-2.174821
H	4.483304	1.816364	-1.660307
H	2.408488	0.705072	-0.939888
C	-0.050161	-3.153285	1.731549
C	0.472613	-3.935726	0.781697
H	-0.381043	-2.137862	1.545296
H	-0.159975	-3.516087	2.746675
H	0.786555	-4.938999	1.063022
C	0.691900	-3.604432	-0.635542

C	1.334800	-4.543151	-1.457465
C	0.287744	-2.388343	-1.212636
C	1.573792	-4.280702	-2.804225
H	1.651321	-5.489536	-1.030282
C	0.524404	-2.125363	-2.556947
H	-0.219699	-1.641530	-0.613037
C	1.169907	-3.068818	-3.359531
H	2.074206	-5.021919	-3.417620
H	0.202708	-1.180149	-2.980077
H	1.353582	-2.859359	-4.407430

=== **TS-8** ===

B	-2.232057	-0.487956	0.088228
B	0.274612	1.021541	-0.473279
C	-3.540269	-1.265618	0.519030
C	-3.499205	-2.453514	1.270620
C	-4.660950	-3.096314	1.696487
C	-5.910450	-2.561624	1.386971
C	-5.983784	-1.377223	0.655745
C	-4.815840	-0.743261	0.233435
C	-1.845698	-0.039097	-1.309101
C	-3.519954	-1.178008	-2.833598
C	-1.639007	0.328487	-3.515932
C	-0.620972	0.976973	-2.900281
C	-0.361389	0.971117	0.919199
C	1.226384	2.269464	2.393841
C	-0.797008	0.969213	3.122131
C	-1.762217	0.236330	2.518335
C	1.475043	2.003752	-0.849186
C	1.192761	3.306490	-1.304998
C	2.202376	4.214086	-1.622085
C	3.540666	3.843319	-1.492415
C	3.850920	2.560647	-1.046880
C	2.831956	1.659219	-0.733496
N	-2.383789	-0.313343	-2.550500
N	-0.736292	0.748797	-1.531699
N	0.065348	1.426431	2.143566
N	-1.497134	0.231336	1.147341
H	-2.539081	-2.887872	1.526146
H	-4.590762	-4.015208	2.269185
H	-6.816114	-3.059106	1.716301
H	-6.950068	-0.943497	0.420019
H	-4.897246	0.188573	-0.317954
H	-3.470264	-2.071099	-2.213543
H	-3.470262	-1.478456	-3.879778
H	-4.468542	-0.667921	-2.649260
H	-1.884913	0.253330	-4.561706
H	0.179631	1.563280	-3.313783
H	2.155372	1.729965	2.199718
H	1.206356	2.579729	3.438380
H	1.201521	3.153469	1.757003
H	-0.660729	1.221832	4.160144
H	-2.615228	-0.269217	2.933388
H	0.157904	3.620558	-1.407474
H	1.945618	5.210644	-1.966375
H	4.330356	4.545190	-1.737456
H	4.888004	2.257593	-0.946286
H	3.107007	0.666963	-0.394278
C	1.034800	-1.055331	-0.277107
C	0.039341	-2.035551	-0.211643
H	1.604424	-0.867124	0.626139
H	1.618505	-0.967686	-1.188573
H	-0.254608	-2.379155	0.775457
C	-0.359259	-2.919645	-1.305326
C	-1.070837	-4.106713	-1.025525
C	-0.030981	-2.680347	-2.655785
C	-1.440316	-4.993110	-2.029898
H	-1.326204	-4.335672	0.003429
C	-0.399365	-3.568637	-3.660784
H	0.517910	-1.785817	-2.923882
C	-1.111507	-4.731179	-3.361601
H	-1.982695	-5.897089	-1.772654
H	-0.124344	-3.352828	-4.688520
H	-1.395117	-5.422940	-4.146642

=== **TS-8*** ===

B	-2.207403	-0.352149	0.190368
B	0.363470	1.057171	-0.419587
C	-3.543819	-1.089998	0.596135
C	-3.706273	-2.481375	0.488656
C	-4.907983	-3.107683	0.818938
C	-5.990155	-2.355095	1.270203
C	-5.857131	-0.971977	1.392804
C	-4.651724	-0.354944	1.062768
C	-1.726465	-0.097169	-1.222820
C	-3.466292	-1.220150	-2.683862
C	-1.412287	-0.004000	-3.447131
C	-0.390604	0.669664	-2.861232

C	-0.353056	1.177078	0.931304
C	1.163626	2.647278	2.315243
C	-0.903210	1.449924	3.091723
C	-1.830458	0.632382	2.540227
C	1.529955	2.056365	-0.867281
C	1.185422	3.301741	-1.428975
C	2.149574	4.231406	-1.815676
C	3.504007	3.940872	-1.653052
C	3.875614	2.714972	-1.106408
C	2.901372	1.790777	-0.723601
N	-2.245070	-0.466299	-2.451381
N	-0.588390	0.634329	-1.486351
N	0.006190	1.787598	2.108795
N	-1.507235	0.469791	1.191051
H	-2.871804	-3.087601	0.157480
H	-4.996220	-4.185599	0.731415
H	-6.925483	-2.839910	1.527952
H	-6.693135	-0.374377	1.741169
H	-4.575484	0.724635	1.156034
H	-3.349097	-2.265671	-2.389579
H	-3.703227	-1.173333	-3.746804
H	-4.292268	-0.793840	-2.114777
H	-1.624470	-0.177129	-4.488868
H	0.447919	1.177576	-3.302881
H	2.093927	2.085118	2.213379
H	1.107322	3.061933	3.321457
H	1.171957	3.462167	1.591636
H	-0.811304	1.818402	4.099445
H	-2.683171	0.150779	2.982110
H	0.136468	3.551430	-1.561424
H	1.844943	5.182355	-2.240535
H	4.258849	4.660333	-1.951227
H	4.925977	2.473803	-0.979715
H	3.225926	0.844332	-0.306605
C	1.210468	-0.897359	-0.074048
C	0.300168	-1.935670	0.135501
H	1.890623	-0.650127	0.735337
H	1.661617	-0.831923	-1.059661
H	-0.083638	-2.457610	-0.734981
C	0.056812	-2.592533	1.411397
C	-0.536840	-3.874012	1.443341
C	0.437440	-2.036043	2.651788
C	-0.737540	-4.556954	2.635718
H	-0.819836	-4.342787	0.505660
C	0.233104	-2.720104	3.845082
H	0.898592	-1.056084	2.679678
C	-0.355790	-3.985246	3.851326
H	-1.188188	-5.543923	2.618916
H	0.537270	-2.262003	4.780856
H	-0.511458	-4.516473	4.783293

=== **8** ===

B	-1.866487	-0.844614	-0.076453
B	0.420002	0.742810	-0.515507
C	-3.296982	-1.500695	0.302198
C	-3.521248	-2.877162	0.458863
C	-4.776304	-3.388701	0.799571
C	-5.855043	-2.531963	1.000780
C	-5.665440	-1.156974	0.857696
C	-4.409449	-0.660844	0.514332
C	-1.842164	0.066012	-1.420764
C	-3.852844	-0.532186	-2.828156
C	-1.973796	1.022755	-3.435414
C	-0.826150	1.445262	-2.836728
C	-0.353689	0.953820	0.898425
C	1.003139	2.489474	2.375729
C	-1.065471	1.211203	3.001696
C	-1.908043	0.351225	2.368700
C	1.613500	1.771402	-0.869073
C	1.374063	3.151880	-1.012458
C	2.391257	4.055107	-1.317149
C	3.699212	3.599777	-1.486339
C	3.968835	2.239695	-1.352063
C	2.938661	1.345269	-1.052182
N	-2.596690	0.160409	-2.546322
N	-0.762406	0.850257	-1.595723
N	-0.096444	1.575698	2.077291
N	-1.458202	0.205348	1.073433
H	-2.707241	-3.572894	0.301713
H	-4.908068	-4.460562	0.906748
H	-6.829885	-2.927100	1.265743
H	-6.495252	-0.474676	1.013206
H	-4.290334	0.415053	0.410042
H	-3.872444	-1.482305	-2.301858
H	-3.915878	-0.714479	-3.901290
H	-4.707738	0.067661	-2.510545
H	-2.396763	1.251164	-4.398797
H	-0.058264	2.106632	-3.198158

H	1.789022	2.365221	1.636994
H	1.397991	2.259179	3.366377
H	0.658990	3.526100	2.357118
H	-1.064217	1.591535	4.009001
H	-2.778477	-0.163490	2.735710
H	0.364742	3.533278	-0.877318
H	2.166969	5.112061	-1.420552
H	4.496261	4.296884	-1.721443
H	4.981372	1.872367	-1.484547
H	3.175278	0.290665	-0.959813
C	0.769648	-0.870483	-0.508767
C	-0.468435	-1.783128	-0.172266
H	1.582514	-1.091022	0.193190
H	1.154524	-1.133701	-1.500387
H	-0.332867	-2.159320	0.849140
C	-0.519052	-3.003078	-1.069328
C	-0.350080	-4.290238	-0.536660
C	-0.702797	-2.908048	-2.458632
C	-0.384023	-5.428992	-1.340731
H	-0.185042	-4.398747	0.531295
C	-0.739311	-4.041359	-3.268212
H	-0.803843	-1.932427	-2.920980
C	-0.584918	-5.311837	-2.714226
H	-0.248025	-6.407732	-0.892335
H	-0.882610	-3.931525	-4.338396
H	-0.611220	-6.193754	-3.344458

=== **8*** ===

B	-1.820339	-0.709672	0.279843
B	0.467837	0.809331	-0.332539
C	-3.261429	-1.350231	0.640263
C	-3.476448	-2.725789	0.820690
C	-4.746953	-3.250229	1.070306
C	-5.851908	-2.406977	1.156150
C	-5.672675	-1.034053	0.985224
C	-4.400636	-0.525177	0.728824
C	-1.723881	-0.068537	-1.220688
C	-3.655388	-0.938253	-2.608230
C	-1.720533	0.447902	-3.396033
C	-0.615409	0.999908	-2.825601
C	-0.406370	1.279581	0.958082
C	0.800013	3.156563	2.145342
C	-1.276528	1.966002	2.900641
C	-2.053790	0.959392	2.419021

C	1.677959	1.775647	-0.791304
C	1.442994	3.079093	-1.270050
C	2.479231	3.924422	-1.664315
C	3.802262	3.488392	-1.586215
C	4.068322	2.204752	-1.115278
C	3.019885	1.366276	-0.729872
N	-2.400637	-0.220991	-2.389100
N	-0.635239	0.676165	-1.486898
N	-0.248960	2.152231	1.986161
N	-1.504828	0.545192	1.223463
H	-2.637547	-3.408544	0.773802
H	-4.869779	-4.319990	1.205240
H	-6.838479	-2.811389	1.355462
H	-6.523513	-0.362936	1.048020
H	-4.294239	0.546876	0.583679
H	-3.786875	-1.692987	-1.839047
H	-3.617178	-1.419627	-3.586238
H	-4.503230	-0.250718	-2.576710
H	-2.080287	0.467221	-4.410658
H	0.175958	1.580988	-3.265574
H	1.645766	2.902618	1.513924
H	1.119323	3.174280	3.188341
H	0.431235	4.145679	1.864371
H	-1.355505	2.556470	3.797538
H	-2.933399	0.502353	2.836887
H	0.422037	3.448062	-1.332247
H	2.257454	4.922278	-2.029346
H	4.613537	4.141305	-1.889603
H	5.092815	1.852558	-1.051231
H	3.255569	0.369498	-0.372667
C	0.838425	-0.753477	0.041559
C	-0.426126	-1.655774	0.306116
H	1.485674	-0.773839	0.924797
H	1.431856	-1.191976	-0.770290
H	-0.523825	-2.331196	-0.552100
C	-0.222291	-2.554307	1.511717
C	-0.073884	-3.939654	1.345791
C	-0.132683	-2.055604	2.821243
C	0.137386	-4.791806	2.429137
H	-0.119636	-4.356439	0.343699
C	0.076406	-2.901087	3.908601
H	-0.227058	-0.991209	2.998800
C	0.210003	-4.276498	3.721187
H	0.249217	-5.858041	2.261230

H	0.135104	-2.483148	4.908448
H	0.373319	-4.933824	4.567965

Formation of **9** and **9***

=== **Ph_2C=C=CH_2** ===

C	-0.258434	-2.527360	-1.131898
C	-1.169005	-3.432902	-0.841145
C	-2.329868	-3.646702	-1.758401
C	-3.608685	-3.902810	-1.243079
C	-2.174351	-3.564695	-3.148903
C	-4.699320	-4.062622	-2.094178
H	-3.750299	-3.967461	-0.170705
C	-3.264821	-3.726132	-3.999018
H	-1.189401	-3.377293	-3.560917
C	-4.532684	-3.975942	-3.475500
H	-5.681784	-4.252449	-1.676130
H	-3.122534	-3.664057	-5.072299
H	-5.381604	-4.104755	-4.137393
C	0.642048	-1.631501	-1.419031
H	0.560449	-0.607231	-1.063345
H	1.511553	-1.871471	-2.026226
C	-1.041035	-4.246576	0.406394
C	-0.549161	-3.678320	1.589377
C	-1.381003	-5.607036	0.409565
C	-0.393455	-4.447754	2.738984
H	-0.292401	-2.625299	1.603259
C	-1.223432	-6.376434	1.559592
H	-1.761220	-6.065639	-0.495620
C	-0.729911	-5.800742	2.729421
H	-0.015492	-3.988007	3.645523
H	-1.484639	-7.428778	1.540011
H	-0.611661	-6.399665	3.625336

=== **RC** ===

B	-2.573043	0.548923	0.650000
B	-0.089630	1.980403	-0.347670
C	-3.842588	-0.151441	1.281592
C	-3.766030	-1.429376	1.865675
C	-4.880072	-2.045745	2.434381
C	-6.113379	-1.394650	2.438180
C	-6.217958	-0.124601	1.873577
C	-5.097117	0.484225	1.308025
C	-2.296480	0.815894	-0.795599
C	-4.226140	-0.292086	-2.004465
C	-2.307180	0.935942	-3.044381
C	-1.178035	1.553963	-2.621389
C	-0.360095	1.701851	1.094869
C	1.588625	2.771516	2.306334
C	-0.353267	1.579422	3.346937
C	-1.485574	0.970152	2.922548
C	1.153214	2.728750	-0.979147
C	1.018656	4.009787	-1.547641
C	2.101539	4.681550	-2.113404
C	3.362472	4.085659	-2.131342
C	3.524818	2.814241	-1.584233
C	2.434364	2.149771	-1.021481
N	-2.997017	0.479417	-1.950232
N	-1.144674	1.487442	-1.223156
N	0.341352	2.033674	2.252994
N	-1.516502	1.034578	1.523088
H	-2.817994	-1.958576	1.865484
H	-4.786591	-3.035190	2.869888
H	-6.982596	-1.871074	2.878592
H	-7.170901	0.394510	1.878250
H	-5.196639	1.479204	0.884372
H	-4.112349	-1.234398	-1.465892
H	-4.451384	-0.509693	-3.048275
H	-5.060108	0.260027	-1.565065
H	-2.669834	0.777580	-4.046684
H	-0.391115	2.022336	-3.184074
H	2.412114	2.194183	1.878357
H	1.815733	2.994169	3.349155
H	1.505042	3.708851	1.752685
H	0.010762	1.731049	4.349943
H	-2.277109	0.508020	3.483960
H	0.047016	4.495044	-1.538503
H	1.963266	5.671533	-2.535700
H	4.207674	4.605473	-2.569232
H	4.499351	2.337145	-1.600227
H	2.581195	1.154978	-0.611431
C	-0.287986	-3.117423	-1.109361
C	-1.115369	-4.138117	-1.016344
C	-2.158728	-4.340724	-2.068397
C	-3.457735	-4.739539	-1.720868
C	-1.873251	-4.103348	-3.419968
C	-4.440874	-4.889441	-2.695990
H	-3.700475	-4.923883	-0.681033
C	-2.856209	-4.254468	-4.394563
H	-0.870932	-3.802762	-3.702683

C	-4.145050	-4.649006	-4.037253
H	-5.440997	-5.192331	-2.405792
H	-2.611896	-4.073771	-5.435751
H	-4.909387	-4.773166	-4.796230
C	0.519287	-2.100659	-1.201622
H	0.281130	-1.140004	-0.750112
H	1.460469	-2.171282	-1.741859
C	-1.015163	-5.086171	0.134184
C	-0.626182	-4.637803	1.404643
C	-1.273480	-6.453741	-0.038910
C	-0.493638	-5.527828	2.466338
H	-0.426455	-3.582925	1.554223
C	-1.140058	-7.344055	1.023974
H	-1.570412	-6.823118	-1.013251
C	-0.750678	-6.886072	2.281471
H	-0.195044	-5.159040	3.441555
H	-1.337495	-8.398819	0.866890
H	-0.650414	-7.579228	3.109061

=== **TS-9** ===

B	-2.185700	-0.684257	0.138728
B	0.375033	0.815552	-0.425392
C	-3.492164	-1.454478	0.573613
C	-3.437414	-2.579467	1.418003
C	-4.594792	-3.228904	1.845870
C	-5.847802	-2.761667	1.451661
C	-5.931885	-1.638719	0.629882
C	-4.770149	-0.998907	0.200024
C	-1.706150	-0.368316	-1.263621
C	-3.386159	-1.521163	-2.787332
C	-1.393880	-0.190165	-3.478265
C	-0.403071	0.504722	-2.868985
C	-0.291316	0.761036	0.961632
C	1.101951	2.274820	2.459086
C	-0.894523	0.940219	3.122268
C	-1.785327	0.117169	2.528487
C	1.392546	1.995426	-0.788509
C	0.941306	3.076227	-1.573869
C	1.749918	4.172561	-1.869185
C	3.054979	4.231379	-1.383086
C	3.528677	3.186697	-0.592700
C	2.709539	2.095700	-0.302833
N	-2.190907	-0.741808	-2.502576
N	-0.585864	0.402487	-1.496241
N	0.016990	1.347587	2.169412
N	-1.431881	0.012891	1.181820
H	-2.474058	-2.955815	1.746825
H	-4.516839	-4.098443	2.489869
H	-6.749253	-3.262993	1.786655
H	-6.901995	-1.257428	0.328758
H	-4.860465	-0.117723	-0.427847
H	-3.531583	-2.272888	-2.017835
H	-3.250296	-2.025726	-3.743952
H	-4.273568	-0.883088	-2.837395
H	-1.583604	-0.356667	-4.525168
H	0.435622	1.024200	-3.294390
H	2.070100	1.771523	2.432999
H	0.941381	2.679944	3.457917
H	1.112483	3.090994	1.739338
H	-0.831773	1.276710	4.143146
H	-2.642901	-0.388674	2.931527
H	-0.075792	3.072185	-1.951911
H	1.357520	4.983919	-2.473538
H	3.690414	5.079597	-1.612987
H	4.539906	3.218483	-0.200259
H	3.107568	1.308363	0.325628
C	1.382956	-1.249784	-0.460461
C	0.581689	-2.361338	-0.192911
C	0.063429	-3.161600	-1.342475
C	-1.123004	-3.920408	-1.269142
C	0.770044	-3.220235	-2.559386
C	-1.553915	-4.714347	-2.326326
H	-1.729846	-3.872339	-0.375273
C	0.336598	-4.008205	-3.622433
H	1.675211	-2.639464	-2.672274
C	-0.825171	-4.770086	-3.515363
H	-2.470332	-5.286603	-2.222835
H	0.919306	-4.032239	-4.537631
H	-1.157558	-5.392448	-4.338782
C	2.620859	-1.085326	-0.903751
H	3.057152	-0.131308	-1.164941
H	3.270809	-1.952725	-1.032912
C	0.533828	-2.879643	1.185148
C	0.189793	-4.216662	1.497512
C	0.960747	-2.076409	2.268306
C	0.234720	-4.696374	2.802276
H	-0.076401	-4.902631	0.706156
C	0.997715	-2.558178	3.570765

H	1.283760	-1.064022	2.070031
C	0.625413	-3.871592	3.856933
H	-0.025294	-5.732753	2.992267
H	1.328153	-1.903012	4.370466
H	0.656782	-4.248354	4.872892

=== **TS-9*** ===

B	-2.314278	-0.269335	0.498105
B	-0.215988	1.601225	-0.671772
C	-3.666577	-0.669797	1.264197
C	-4.155685	-1.968387	1.459979
C	-5.362020	-2.209199	2.121533
C	-6.121184	-1.149397	2.610406
C	-5.660036	0.155310	2.433404
C	-4.454349	0.383569	1.773198
C	-2.354417	0.292922	-0.936881
C	-4.549037	-0.561406	-1.863211
C	-2.825339	0.790342	-3.074982
C	-1.624675	1.348007	-2.795101
C	-0.351465	1.327807	0.803543
C	1.511462	2.699696	1.834738
C	-0.184701	1.301865	3.044849
C	-1.246381	0.528188	2.705695
C	0.919751	2.431602	-1.385723
C	0.687178	3.721608	-1.897763
C	1.696424	4.454566	-2.521297
C	2.973529	3.911118	-2.656570
C	3.229026	2.631509	-2.166916
C	2.214805	1.905519	-1.542402
N	-3.285545	0.163156	-1.937646
N	-1.335930	1.078194	-1.453796
N	0.376836	1.792361	1.888204
N	-1.359680	0.532040	1.320864
H	-3.587994	-2.811470	1.092163
H	-5.704790	-3.229844	2.256323
H	-7.058283	-1.335172	3.124182
H	-6.239416	0.993076	2.807940
H	-4.116572	1.408329	1.645687
H	-4.376617	-1.636064	-1.793647
H	-5.113690	-0.349952	-2.770674
H	-5.121950	-0.233150	-0.997675
H	-3.394941	0.789397	-3.988672
H	-0.963714	1.929652	-3.411566
H	2.366997	2.232404	1.343267
H	1.787965	2.965734	2.854722
H	1.252941	3.607440	1.286730
H	0.213636	1.545225	4.015699
H	-1.925036	-0.026993	3.327722
H	-0.298305	4.167150	-1.797211
H	1.487471	5.450267	-2.898180
H	3.760237	4.478553	-3.141539
H	4.216755	2.196054	-2.275596
H	2.428861	0.905778	-1.177425
C	-0.982736	-2.053490	0.135377
C	-1.568984	-3.237089	-0.287910
C	-2.211387	-3.336509	-1.610399
C	-3.360265	-4.122220	-1.839798
C	-1.665589	-2.650100	-2.713345
C	-3.923863	-4.226161	-3.107706
H	-3.814070	-4.652521	-1.011076
C	-2.227227	-2.757543	-3.981467
H	-0.777656	-2.046879	-2.564768
C	-3.358798	-3.547776	-4.189225
H	-4.809305	-4.836237	-3.252087
H	-1.772757	-2.231297	-4.814280
H	-3.790999	-3.638764	-5.179607
C	0.269618	-1.733968	0.458812
H	0.564245	-0.860972	1.019958
H	1.080144	-2.397090	0.158327
C	-1.486187	-4.424182	0.585621
C	-1.339985	-4.303442	1.984779
C	-1.484254	-5.733173	0.054455
C	-1.198059	-5.419452	2.799881
H	-1.345604	-3.315730	2.428188
C	-1.338952	-6.848139	0.873604
H	-1.562250	-5.874808	-1.015891
C	-1.198260	-6.703902	2.253375
H	-1.095424	-5.286642	3.871856
H	-1.325185	-7.836948	0.427051
H	-1.090049	-7.574318	2.890486

=== **9** ===

B	-1.698613	-0.961021	0.138767
B	0.488811	0.726825	-0.488081
C	-3.101373	-1.605506	0.649614
C	-3.220756	-2.759108	1.442866
C	-4.450472	-3.191911	1.943939
C	-5.612912	-2.472503	1.678914

C	-5.528455	-1.309564	0.914543
C	-4.294539	-0.891829	0.418537
C	-1.693850	-0.265463	-1.344099
C	-3.525141	-1.316340	-2.748919
C	-1.719363	0.222879	-3.523927
C	-0.643422	0.835573	-2.965629
C	-0.381371	1.090671	0.837692
C	0.678234	3.066810	2.015017
C	-1.325749	1.744001	2.752010
C	-2.014210	0.668451	2.285593
C	1.526533	1.916531	-0.875823
C	1.055947	3.066073	-1.544475
C	1.868263	4.167248	-1.814032
C	3.201865	4.168181	-1.409412
C	3.694134	3.061986	-0.722291
C	2.869018	1.966546	-0.460182
N	-2.364221	-0.462143	-2.508366
N	-0.647299	0.540409	-1.618079
N	-0.311009	2.002282	1.840941
N	-1.411814	0.274981	1.108824
H	-2.337353	-3.332336	1.693776
H	-4.494972	-4.091057	2.549728
H	-6.568606	-2.806928	2.067912
H	-6.421000	-0.727692	0.707746
H	-4.259233	0.027209	-0.160638
H	-3.692989	-1.953606	-1.888549
H	-3.327299	-1.941786	-3.619690
H	-4.414778	-0.707643	-2.926680
H	-2.075515	0.202894	-4.539709
H	0.118892	1.439051	-3.423857
H	1.650022	2.648276	2.277995
H	0.336861	3.716270	2.820459
H	0.779293	3.646507	1.100642
H	-1.464266	2.345825	3.633673
H	-2.867788	0.158154	2.694549
H	0.015747	3.120520	-1.847658
H	1.456776	5.026381	-2.334231
H	3.841477	5.018882	-1.617762
H	4.724782	3.049036	-0.382573
H	3.288121	1.149638	0.110368
C	0.945826	-0.831781	-0.181967
C	-0.223492	-1.865783	0.022943
C	-0.371652	-2.765758	-1.234460
C	-1.364955	-3.762886	-1.282622
C	0.421240	-2.622862	-2.382391
C	-1.548439	-4.571410	-2.400943
H	-2.002899	-3.923421	-0.425641
C	0.235005	-3.423373	-3.511882
H	1.205531	-1.880367	-2.398539
C	-0.748445	-4.406385	-3.531275
H	-2.318857	-5.335580	-2.384642
H	0.874346	-3.275741	-4.376247
H	-0.885814	-5.036518	-4.403270
C	2.206137	-1.271880	-0.106152
H	3.061983	-0.639642	-0.297557
H	2.441404	-2.308493	0.121953
C	0.104828	-2.709739	1.274154
C	0.452011	-4.067447	1.208025
C	0.147326	-2.114531	2.548087
C	0.787686	-4.799027	2.349821
H	0.467716	-4.573535	0.252539
C	0.475161	-2.838900	3.689028
H	-0.068955	-1.062019	2.657933
C	0.793537	-4.194300	3.601354
H	1.051109	-5.846899	2.249440
H	0.487175	-2.339024	4.652133
H	1.050755	-4.760705	4.489517

=== **9*** ===

B	-2.634419	-0.262608	0.065243
B	-0.002967	0.661958	-0.370800
C	-4.164812	-0.454701	0.555396
C	-4.544029	-1.474607	1.442930
C	-5.823276	-1.540387	1.995172
C	-6.773712	-0.570001	1.683790
C	-6.421913	0.473316	0.829406
C	-5.137307	0.526113	0.287471
C	-2.331712	0.461402	-1.377700
C	-4.350207	0.267968	-2.902141
C	-2.073684	1.105327	-3.507315
C	-0.890296	1.283773	-2.862799
C	-0.762363	1.269390	0.926365
C	0.804824	2.743221	2.250963
C	-1.514973	2.024234	2.889157
C	-2.482959	1.249771	2.330949
C	1.440167	1.282306	-0.744813
C	1.585760	2.620358	-1.159793
C	2.828010	3.171378	-1.471339

C	3.981026	2.392343	-1.369754
C	3.871045	1.065915	-0.959057
C	2.619424	0.524831	-0.656867
N	-2.963758	0.589930	-2.575713
N	-1.064474	0.891354	-1.554126
N	-0.442906	2.025084	2.005921
N	-2.001092	0.788148	1.123345
H	-3.822476	-2.234781	1.713041
H	-6.077160	-2.351432	2.669741
H	-7.770972	-0.620453	2.107563
H	-7.142662	1.249651	0.592837
H	-4.882488	1.373006	-0.343568
H	-4.772939	-0.364201	-2.128509
H	-4.377521	-0.260903	-3.856433
H	-4.946360	1.180004	-2.982491
H	-2.363390	1.294862	-4.526828
H	0.052255	1.647472	-3.232311
H	1.558244	2.410170	1.544037
H	1.145478	2.536587	3.267005
H	0.655960	3.819176	2.133609
H	-1.488860	2.568139	3.818024
H	-3.461794	0.993475	2.695544
H	0.705263	3.253842	-1.235091
H	2.898618	4.206567	-1.789937
H	4.950971	2.814795	-1.608938
H	4.759633	0.448334	-0.877421
H	2.563718	-0.512691	-0.345545
C	-1.515451	-1.492141	-0.026176
C	-1.711598	-2.822855	-0.211060
C	-3.015439	-3.493654	-0.504953
C	-3.444198	-4.599212	0.248091
C	-3.801149	-3.106712	-1.597070
C	-4.635038	-5.254255	-0.047804
H	-2.836717	-4.942101	1.078658
C	-4.989965	-3.767003	-1.905379
H	-3.454283	-2.295566	-2.224402
C	-5.417787	-4.838583	-1.125851
H	-4.951705	-6.095668	0.559350
H	-5.578199	-3.450050	-2.760430
H	-6.342823	-5.352930	-1.361409
C	-0.074242	-0.953081	-0.045798
H	0.420873	-1.133967	0.916678
H	0.506653	-1.515014	-0.784916
C	-0.564211	-3.797551	-0.197803
C	0.198831	-4.007733	0.958584
C	-0.257545	-4.558584	-1.334928
C	1.239293	-4.935102	0.976681
H	-0.033236	-3.440146	1.853075
C	0.790509	-5.476401	-1.325541
H	-0.848261	-4.427581	-2.235227
C	1.543284	-5.670340	-0.167908
H	1.811016	-5.085350	1.886457
H	1.015827	-6.045357	-2.221328
H	2.354096	-6.390351	-0.156709

Supplementary Table 8. Optimized geometries of **2'**, **annulated indole**, **2"**, **2"+2H**, **C_6H_8** and **C_6H_6** (atom, x-, y-, z-positions in Å).

=== **2'** ===

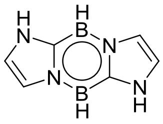

N	2.559492	-1.08072	0.00013	B	-0.02856	-1.50445	-0.000084
C	3.364642	0.032211	0.00028	C	1.22241	-0.70273	-0.000273
C	2.562223	1.124436	-0.000077	H	2.870168	-2.03622	-0.000189
N	-2.55947	1.080729	0.000216	H	4.439693	-0.0343	0.000588
C	-3.36466	-0.03221	0.000034	H	2.814608	2.170133	-0.000067
C	-2.56221	-1.12443	-0.000004	H	-2.8701	2.03625	-0.000316
N	1.227745	0.694109	-0.00026	H	-4.43971	0.034311	0.000126
B	0.028538	1.504437	-0.000048	H	-2.81459	-2.17013	0.00007
C	-1.22241	0.702705	0.000032	H	0.107236	2.691279	-0.000004
N	-1.22775	-0.69411	-0.000015	H	-0.10731	-2.69128	-0.000003

=== **annulated indole** ===

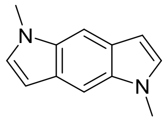

C	-1.08775	-0.83546	0.002968	C	2.603226	2.843835	0.014261
C	-1.27504	0.585469	0.045156	H	-0.28572	2.515027	0.073006
C	-0.1652	1.43814	0.042049	H	0.285721	-2.51503	-0.07301
C	1.087745	0.835456	-0.00297	H	-4.34553	-0.67158	0.08321
C	1.275043	-0.58547	-0.04516	H	-3.20095	1.761324	0.121541
C	0.165197	-1.43814	-0.04205	H	4.345532	0.671582	-0.08321
N	-2.34518	-1.42079	0.01657	H	3.200951	-1.76132	-0.12154
C	-3.29626	-0.41826	0.06549	H	-3.6801	-3.01114	0.004967
C	-2.69383	0.809886	0.084204	H	-2.19809	-3.29849	-0.92432
N	2.34518	1.420787	-0.01657	H	-2.15921	-3.34406	0.85274
C	3.296263	0.418258	-0.06549	H	2.159209	3.344058	-0.85274
C	2.693834	-0.80989	-0.0842	H	2.198094	3.298488	0.924317
C	-2.60323	-2.84384	-0.01426	H	3.680101	3.011144	-0.00497

=== **2"** ===

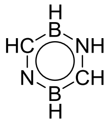

C	1.871378	0.929715	-0.564181	C	-0.992731	1.003206	-0.565137

H	-0.799606	-1.386854	-0.563563	H	-2.070842	1.129136	-0.565559
H	1.687537	-1.079710	-0.562964	B	-0.287715	-0.314444	-0.564081
H	2.949490	0.803792	-0.563759	B	1.166357	2.247370	-0.565237
H	1.678264	3.319772	-0.565755	N	1.150349	-0.219530	-0.563683
H	-0.808892	3.012633	-0.566353	N	-0.271699	2.152456	-0.565634

=== **2” + 2H** ===

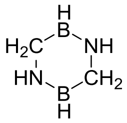

C	1.967489	0.920195	-0.564000	H	-1.759224	1.012069	-1.437035
C	-1.088840	1.012626	-0.564959	B	-0.248161	-0.325865	-0.564062
H	-0.806583	-1.383410	-0.563663	B	1.126807	2.258700	-0.565273
H	1.657540	-1.161895	-0.563062	N	1.139706	-0.294023	-0.563718
H	2.638392	0.919694	-1.435679	N	-0.261058	2.226855	-0.565678
H	1.685236	3.316240	-0.565913	H	2.637408	0.920764	0.308443
H	-0.778898	3.094725	-0.566489	H	-1.759281	1.013128	0.307085

=== **C_6H_8** ===

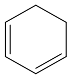

C	0.009680	0.161994	-0.234330	H	1.457997	-0.147668	1.296978
C	1.448056	0.082218	0.219899	H	3.148196	1.400852	0.510724
C	2.199644	1.399089	-0.033279	H	1.867622	3.518107	0.630404
C	1.364850	2.601053	0.340296	H	-0.573597	3.431455	0.459137
C	0.028080	2.555814	0.238028	H	-1.693910	1.385499	-0.482549
C	-0.646887	1.331282	-0.202453	H	1.965433	-0.746248	-0.271712
H	-0.499006	-0.751580	-0.525165	H	2.459656	1.477101	-1.100685

=== **C_6H_6** ===

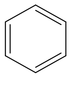

C	-0.101682	0.116230	0.000066	H	-0.643928	-0.823046	0.000435
C	1.292703	0.116199	0.000561	H	1.834822	-0.823163	0.001366
C	1.989899	1.323534	-0.000094	H	3.074451	1.323633	0.000189
C	1.292722	2.531162	-0.001029	H	1.835208	3.470296	-0.001439
C	-0.101450	2.531187	-0.001426	H	-0.643797	3.470414	-0.002263
C	-0.798750	1.323669	-0.000975				

Supplementary Methods

Crystallographic Methods: X-ray data collection and structural refinement. Intensity data for compounds **1-4, 5a, 6-10** and **11b** were collected using a Bruker APEX II diffractometer. The crystals of **1-4, 5a, 6, 7, 9, 10** and **11b** were measured at 103(2) K and the crystals of **8** were measured at 153(2) K. The structure was solved by direct phase determination (SHELXS-2014) and refined for all data by full-matrix least squares methods on F^2. All non-hydrogen atoms were subjected to anisotropic refinement. The hydrogen atoms were generated geometrically and allowed to ride in their respective parent atoms; they were assigned appropriate isotropic thermal parameters and included in the structure-factor calculations. CCDC: 1442910-1442920 contains the supplementary crystallographic data for this paper. The data can be obtained free of charge from the Cambridge Crystallography Data Center via www.ccdc.cam.ac.uk/data_request/cif.

Computational Methods

Gaussian 09 was used for all density functional theory (DFT) calculations including geometry optimization, frequency calculations, Natural bond orbital (NBO) analysis, nucleus-independent chemical-shift (NICS) calculations, estimation of the resonance stabilization energy (RSE), and mechanistic study.

Optimization, frequency calculations, NBO analysis, NICS calculation and RSE estimation were performed at the B3LYP/6-311+G(d,p) level of theory.

Mechanistic study for the formation of **3B**, **4**, **5a**, **6***, **8**, **9** from **2** were performed at the B3LYP-D3(BJ)(SCRF)/6-311+G(d,p)//B3LYP/6-311+G(d,p) level (solvent = benzene) with and without dispersion force.

Supplementary References

1. Sheldrick, G. M. SHELXL-97, Program for Crystal Structure Refinement; University of Göttingen: Göttingen, Germany, **1997**.
2. Bruker AXS SHELXTL, Madison, WI; SHELX-97G. M. Sheldrick, *Acta Crystallogr. A*, **2008**, *64*, 112–122, *SHELX-2013*, http://shelx.uni-ac.gwdg.de/SHELX/index.php.
